# Modeling Time-On-Stream Catalyst Reactivity in the Selective Hydrogenation of Concentrated Acetylene Streams under Industrial Conditions via Experiments and AI

**DOI:** 10.1021/acscatal.5c02226

**Published:** 2025-07-11

**Authors:** Jonathan M. Mauß, Klara S. Kley, Rohini Khobragade, Nguyen-Khang Tran, Jacopo de Bellis, Ferdi Schüth, Matthias Scheffler, Lucas Foppa

**Affiliations:** † 28314 Max-Planck-Institut für Kohlenforschung, Kaiser-Wilhelm-Platz 1, Mülheim an der Ruhr 45470, Germany; ‡ 28259 The NOMAD Laboratory at the Fritz Haber Institute of the Max Planck Society, Faradayweg 4-6, Berlin 14195, Germany

**Keywords:** artificial intelligence, subgroup discovery, symbolic regression, SISSO, acetylene, selective hydrogenation, mechanochemistry

## Abstract

Describing heterogeneous catalysis is complicated by the intricate interplay of processes that govern catalyst performance. The evolving chemical environment and the kinetics of catalyst’s structural changes during reactions often lead to unknown local geometries and chemistry, which can shift reactivity over time. Here, we perform systematic experiments and apply a focused artificial-intelligence (AI) approach to model the measured time-on-stream-dependent reactivity of palladium-based bimetallic catalysts. These materials are synthesized via mechanochemistry and applied in the selective hydrogenation of concentrated acetylene streams(>14.0 vol %)under industrially relevant pressures (10 bar), resulting from a hypothetical electric plasma-assisted methane-to-ethylene process. Unlike the well-established hydrogenation of diluted acetylene (0.1 to 2.0 vol %) streams of naphtha steam cracking, the hydrogenation of concentrated acetylene streams remains largely underexplored due to the harsh reaction conditions and the explosive nature of acetylene. This precludes *operando* characterization or atomistic simulations to investigate catalyst time-on-stream behavior under realistic conditions. Our AI approach first uses subgroup discovery to identify descriptions of materials and reaction conditions resulting in noticeable acetylene conversion. Then, it models time-dependent selectivity focused on high acetylene conversion via the sure-independence-screening-and-sparsifying operator symbolic-regression approach. AI identifies key experimental and theoretical physicochemical descriptive parameters correlated with the reactivity, which highlight the critical interplay between the material structure and the chemical potential of the reaction mixture. The AI models enable the design of bimetallic and trimetallic catalysts, which are experimentally validated.

## Graphical abstract

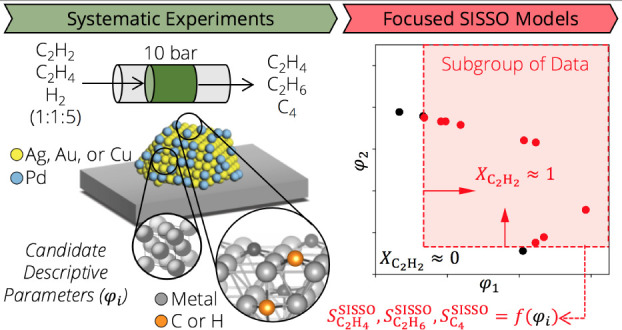

## 1 Introduction

Heterogeneous catalysis is governed by a concerted and intricate interplay of several processes that take place at multiple time and length scales.^1–4^ The chemical environment that exists during catalyst operation as well as the kinetics of catalyst’s structural changes create configurations of typically unknown local geometries and chemistry. While surface reactions that break and make chemical bonds occur at time scales of picoseconds, they produce species such as carbon, hydrogen, or oxygen, that interact with the material initially placed in the reactor and may induce its local restructuring along with changes in the reactivity in the scale of hours, days, or even longer. Indeed, the catalytic performance of a material, i.e., its ability to more or less selectively convert reactants, typically changes in the beginning of the reaction during the so-called *induction period*. During this induction period, the activity and/or the selectivity can drastically change until the surface chemistry reaches a steady state under the applied reaction conditions. At the steady state, a constant catalytic performance is observed with respect to time. At longer reaction times, the catalytic performance might change again, e.g., the activity might decrease. This latter process is known as *deactivation*. The period of time during which the catalytic process is operated is referred to as *time on stream* (*t*_OS_).

Modeling the evolution of the reactivity with *t*_OS_ is crucial for the design of catalytic materials and processes, as it describes the time span during which the material can effectively perform the desired chemical reaction. However, modeling the full catalytic progression at realistic temperatures, pressures and at the long time scales required to capture the *t*_OS_ evolution of the reactivity from first-principles is an inappropriate concept. In spite of recent descriptor-based catalyst design approaches,^5–10^ systematic strategies for modeling the *t*_OS_-dependent catalytic behavior and for the design of improved materials are not well established.

In order to model the intricacy of heterogeneous catalysis, we put forward a data-centric approach based on consistent experimental data and artificial intelligence (AI).^11,12^ This strategy may capture the multidimensional catalytic progression better than previous computational methods because it targets correlations and does not assume a single underlying physical model. The goal of our approach is to identify the key experimental or calculated physical descriptive parameters that correlate with the reactivity rather than explicitly describe all the underlying physical processes that trigger, just facilitate, or hinder the activity or selectivity. Crucially, the key descriptive parameters are identified from many offered *candidate descriptive parameters*, also called *primary features*. In analogy to genes in biology, the selected key parameters might be referred to as ″materials genes″ of catalysis,^12^ since they correlate with the catalytic function of interest. The concept has been previously applied to describe the steady-state reactivity in alkane oxidation^12–14^ CO oxidation,^15^ and CO_2_ hydrogenation^16^ by using global models. However, the relevance and weights of the different underlying physical processes may be very different for different materials or reaction conditions. This questions the suitability of a single, global AI model to describe all situations simultaneously^17^ and calls for approaches that can focus on specific materials or reaction conditions of interest.

Here, we introduce a *focused* AI approach based on subgroup discovery (SGD)^18–21^ and on the sure-independence-screening-and-sparsifying-operator (SISSO) approach^22,23^ to model the evolution of catalyst reactivity with *t*_OS_ in the selective hydrogenation of highly concentrated acetylene (>14 vol %) in ethylene streams under industrially relevant pressures of 10 bar (Figure 1). By *focused*, we mean that the AI models describe in greater detail the situations resulting in noticeable acetylene conversion. Equimolar acetylene-ethylene mixtures would result from a hypothetical electric plasma-assisted methane-to-ethylene conversion plant. The mentioned plasma-assisted process can enable the production of ethylene, one of the most frequently used platform molecules in the chemical industry, from natural gas, biogas or hydrogenated CO_2_ using the short-term surpluses in electricity from renewable sources.^24–26^ However, acetylene formed as a byproduct in equimolar concentrations during plasma activation needs to be selectively converted to ethylene in a dedicated, separate downstream process.^27^ New, more robust catalyst materials are required for this purpose. However, investigating catalyst reactivity at such high acetylene concentrations and industrially relevant pressures requires sophisticated safety measures due to the inherent explosive nature of acetylene, which has a Gibbs free-energy of formation of Δ*G*_f_^0^ = +209 kJ/mol.^28^ Besides, the acetylene hydrogenation reactions to ethylene or ethane are highly exothermic, with reaction enthalpy of Δ*H*_r_^0^ = −175 kJ/mol or Δ*H*_r_^0^ = −312 kJ/mol, respectively.^28^ Difficulties to implement those safety measures into analytical techniques impede the use of *in situ* or *operando* spectroscopy for catalyst characterization under the applied reaction conditions. Thus, AI approaches that identify correlations between basic materials parameters and the reactivity under such challenging conditions are essential to elucidate catalyst behavior and to enable the design of improved or new materials.

**Figure 1:**
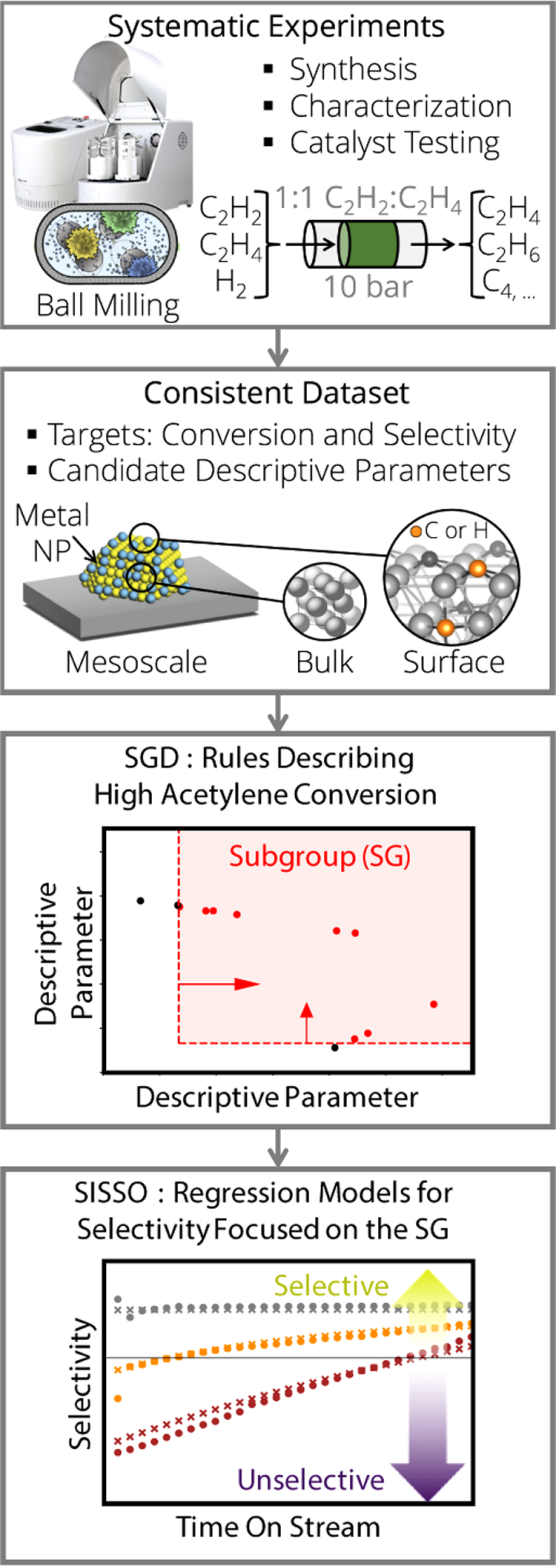
AI approach for modeling the time-on-stream dependent reactivity of palladium-based alloys for the selective hydrogenation of concentrated acetylene streams. Systematic experiments and atomistic simulations are used to create a consistent data set containing the measured reactivity and many candidate descriptive parameters. Then, the subgroup-discovery (SGD) and the sure-independence-screening-and-sparsifying-operator (SISSO) approaches are applied to describe acetylene conversion and selectivity, respectively.

We stress that the catalytic hydrogenation of concentrated acetylene streams discussed in this paper is drastically different from the academically and industrially well studied process for selective hydrogenation of acetylene traces (between 0.1 and 2 vol %) formed alongside ethylene during the steam-cracking of naphtha.^24,29–31^ As highly reactive compound, acetylene is prone to poison catalysts in the downstream processing of ethylene, e.g., polymerization to polyethylene. Therefore, traces of acetylene are removed almost completely from the ethylene streams (down to <5 ppm).^32–39^ For such process, efficient catalysts have been developed. However, this is not the case for the selective hydrogenation of concentrated acetylene. Indeed, this latter process remains largely unexplored and only few studies are reported.^40,41^ Thus, there are opportunities for tuning known catalyst materials and for discovering new ones.

Recently, we took advantage of a mechanochemical procedure^42–44^ to synthesize palladium–silver alloy nanoparticles (NPs) supported on high-surface-area α-Al_2_O_3_ (HSA-α-Al_2_O_3_) as catalysts for the selective hydrogenation of highly concentrated acetylene streams.^40^ The mechanochemical (dry) synthesis of catalytic materials^42–44^ offers a higher atom efficiency and lower environmental impact compared to wet methods.^45^ Furthermore, the high-energy conditions provided by the mechanochemical treatment can provide materials with improved properties compared to materials obtained by traditional methods, e.g., materials with higher surface area.^46^ The synthesized palladium–silver materials display a similar high selectivity towards ethylene but significantly lower deactivation with *t*_OS_ under the harsh reaction conditions compared to materials synthesized by wet impregnation.^40^ Thus, mechanochemistry is promising for obtaining high-performant catalysts whose reactivity is maintained at large *t*_OS_. However, systematic approaches to navigate the immense space of possible materials that can be made mechanochemically are not yet available. Here, we combine the advantages of the mechanochemical synthesis with focused AI and establish an approach for the design of materials for the selective hydrogenation of concentrated acetylene streams. The AI models suggest modifications of bimetallic Pd–Ag materials as well as a trimetallic Pd–Ag–Cu system, which were tested experimentally and present overall comparable ethylene selectivities as the best performing catalysts in the training set. Moreover, the AI models indicate Pd–Ru as new systems that could overperform the known catalysts.

## 2 Methods

### 2.1 Material Synthesis and Catalytic Testing

The materials were synthesized mechanochemically via ball milling in yttria-stabilized zirconia-lined equipment according to previously published procedures.^42–44,47^ Metal powders and γ-AlOOH (bohemite) precursors were subjected to ball-milling procedures, leading to the formation of metal NPs supported on HSA-α-Al_2_O_3_ with a relative amount of metal with respect to support, in weight, of ca. 1%, noted 1 wt %. The synthesized materials were subjected to a thermal activation procedure up to 600^◦^C to ensure a high thermal stability of the NPs under reaction conditions. Further synthesis details are available in the Electronic Supporting Information (ESI).

The catalytic tests were performed in a steel-lined continuous-flow fixed-bed reactor at 10 bar pressure. The oven surrounding the reactor was heated to a set temperature (*T*_oven_) of 50, 100, or 150 °C. The applied feed contained a C_2_H_2_:C_2_H_4_:H_2_ ratio of 1:1:5 at a weight hourly space velocity of 90,000 cm^3^ g_cat_^–1^ h^–1^. The acetylene conversion (*X*_C_2_H_2__) was evaluated as

1 $$X_{C_{2}H_{2}}=1-\frac{n_{C_{2}H_{2},\mathrm{out}}}{n_{C_{2}H_{2},\mathrm{in}}}$$

Here, *n*_C_2_H_2_, out_ and *n*_C_2_H_2_, in_ are molar flow rates of acetylene leaving and entering the reactor, respectively. The values of *X*_C_2_H_2__ are in the range [0,1]. The values of zero and one correspond, respectively, to inactive materials and reaction conditions, and to 100% or full acetylene conversion. The selectivity toward the formation of ethylene (*S*_C_2_H_4__) was evaluated as

2 $$S_{C_{2}H_{4}}=\frac{n_{C_{2}H_{4},\mathrm{out}}-n_{C_{2}H_{4},\mathrm{in}}}{n_{C_{2}H_{2},\mathrm{in}}-n_{C_{2}H_{2},\mathrm{out}}}$$

In eq 2, *n*_C_2_H_4_, out_ and *n*_C_2_H_4_, in_ are molar flow rates of ethylene leaving and entering the reactor, respectively. Note that if *X*_C_2_H_2__ = 0, the denominator of eq 2 is equal to zero. In this case, *S*_C_2_H_4__ = 0 by convention. The values of *S*_C_2_H_4__ are in the range [−1,1]. Ethylene is both a product of the acetylene selective hydrogenation reaction and part of the reaction feed. *S*_C_2_H_4__ < 0 indicates that more ethylene from the feed was consumed than formed from acetylene, with *S*_C_2_H_4__ = −1 (or, equivalently −100%) corresponding to the full consumption of the ethylene of the feed. *S*_C_2_H_4__ > 0 indicate selective materials and conditions. The ideal, desired behavior corresponds to *S*_C_2_H_4__ = 1 (or 100%). In addition to the formation of the desired ethylene, the undesirable formation of the total-hydrogenation product ethane and the formation of oligomeric species (e.g., C_4_) were quantified. The selectivities toward the formation of these products are denoted *S*_C_2_H_6__ and *S*_C_4__, respectively. The product gas composition was measured at each 13.5 min during *t*_OS_ via an online gas chromatograph. We focus our analysis on the initial stages of the reaction with *t*_OS_< 405 min.Additional details on the catalytic tests are provided in ESI and elsewhere.^40,41,48–50^

### 2.2 Focused AI Approach

The two-step focused AI modeling approach takes into account two different materials design criteria or targets, (i) the acetylene conversion and (ii) the selectivity toward ethylene, ethane or C_4_ products. AI could be in principle used to model rate constants of of micro-, meso-, and macroscopic processes, which are then used to create a kinetic model. However, this strategy would require a comprehensive kinetic model and reliable measurements of all rate constants, which are both not available. Therefore, the most viable approach is to directly model well-defined and experimentally measurable performance quantity such as the conversion or the selectivity. In the first step of our focused AI, subgroup discovery (SGD),^18,19,21^ is applied to identify descriptions of subsets of materials and reaction conditions that exhibit noticeable acetylene conversion. The descriptions identified by SGD are, in this work, inequalities constraining the values of few, key parameters, out of many offered candidate descriptive parameters or primary features. Crucially, we note that SGD is not a global approach, but it rather identifies partitions of the data space that present an outstanding distribution of a given target of interest, in our case *X*_C_2_H_2__. In the second step, the symbolic-regression SISSO approach^22,51^ is used to model the *t*_OS_-dependent selectivity for the subset of materials and conditions identified by SGD in the first step. SISSO identifies (nonlinear) analytical expressions that correlate with the selectivity. These expressions also depend on few, key parameters, from the many offered primary features. Thus, both SGD and SISSO are able to identify important primary features.

The outcome of the AI analysis is a SGD model that indicates whether a given material and reaction condition are associated with noticeable conversion. This classification is then subsequently quantified by a SISSO analysis which predicts the selectivity for the materials and reaction conditions that provide acetylene conversion. We note that global AI and machine-learning approaches are likely to fail in the search for the exceptional. SISSO, by design, is a global approach designed to minimize the average error. This means that it primarily optimizes for the irrelevant majority of data rather than the critical ones. The SISSO models for selectivity (e.g., toward ethylene or ethane) obtained in this paper do not attempt to describe all materials and reaction conditions simultaneously, but they focus on the situations of interest that were first identified by SGD. Therefore, in this *focused AI approach*, SGD identifies a description of a specific data space in which the SISSO model is trained. Approaches combining SISSO and SGD were previously applied to catalyst discovery.^52–54^ In some of these works, SISSO models were fit to the entire data set and SGD was then used to obtain key parameters describing high values of the target modeled by SISSO.^53,54^ Besides, SGD was used to identify subsets of materials associated with appropriate adsorption energies, which were then used to train a SISSO model for the same target quantity.^52^ However, the integration of SGD and SISSO for obtaining models for selectivity focused on materials and reaction conditions providing noticeable reactant conversion, i.e., utilizing two different target quantities, is a methodological novelty. Further details on SGD and SISSO are given in the ESI.

As candidate descriptive parameters characterizing the materials and reaction conditions, we collected four parameters from the experimental characterization of the materials by energy dispersive X-ray analysis in a scanning electron microscope (EDX-SEM), high-resolution transmission-electron microscopy (TEM), and N_2_ physisorption, such as the mean particle diameter (*D*_μ_) and the specific surface area (*s*_BET_). Additionally, we included three experimental elemental (free-atom) properties and three experimental bulk properties as parameters reflecting the chemistry of the bulk of the metal NPs, such as the ionization potential (*IP*) and the closest interatomic distance (*d*_closest_). Finally, we utilized nine parameters reflecting the properties of the surfaces of the metal NPs and the interaction of carbon and hydrogen with the surface and subsurface. These parameters were calculated by density functional theory under the generalized gradient approximation (DFT-GGA) on low-index model surfaces. Examples of such parameters are the energy of the *d*-band center­(ϵ_*d*_),^55^ and the binding energy of subsurface hydrogen and carbon (*E*_b,H_^sub^ and *E*_b,C_^sub^, respectively).^56,57^ Carbon and hydrogen (sub)­surface species were suggested as crucial in the selective hydrogenation of diluted acetylene streams on palladium-based catalysts.^58–60^ The elemental, bulk, and surface-related properties were converted into materials-specific parameters by taking the composition average, indicated by the bar in $\overset{-}{\phi }$ , where ϕ is an elemental, bulk, or surface parameter. Finally, *t*_OS_ and *T*_oven_ were used as parameters related to the applied reaction conditions. In total, 21 candidate descriptive parameters were collected. A full list of these parameters is provided in Table S1. Details on the experimental or theoretical methods used to obtain the candidate descriptive parameters are also available in the ESI.

## 3 Results and Discussion

### 3.1 Reactivity of Mechanochemically Synthesized Alloys towards the Selective Hydrogenation of Concentrated Acetylene Streams

Twelve materials constituted of HSA-α-Al_2_O_3_-supported metal NPs were synthesized via the mechanochemical approach.^40,42–44^ These are three monometallic systems (Ag, Au, and Cu) and nine bimetallic alloys with nominal molar ratios 1:1, 1:5, and 1:9, namely Pd_1_Ag_1_, Pd_1_Ag_5_, Pd_1_Ag_9_, Pd_1_Au_1_, Pd_1_Au_5_, Pd_1_Au_9_, Pd_1_Cu_1_, Pd_1_Cu_5_, Pd_1_Cu_9_. We choose the Pd–Ag systems as benchmarks, since they were extensively studied in the context of the selective hydrogenation of diluted acetylene streams. The choice of Pd–Au and Pd–Cu systems, in turn, is based on the structural similarities with the Pd–Ag systems (face-center-cubic solid solutions), which enable an efficient identification of correlations focusing on the composition. These materials were tested in the selective hydrogenation of concentrated acetylene streams (>14.0 vol %) in the presence of equimolar amounts of ethylene and a 5-fold excess of hydrogen at 10 bar pressure and temperatures of 50, 100, or 150 ^◦^C.

In terms of activity, two extreme situations are observed. Either the materials and reaction conditions applied led to the complete conversion of acetylene, or hardly any conversion of acetylene was observed. Online temperature measurements in the catalyst bed during the reaction show the formation of pronounced hot spots. In the case of an active and nonselective catalyst, the temperature of these hot spots could deviate from the set furnace temperature by up to +160 °C. Thus, it is likely that the high exothermicity of the hydrogenation reaction combined with the high acetylene concentration prevents active catalysts from stabilizing at acetylene conversions below 100% without sophisticated cooling systems. We refer at this point to the work from Van Heerden on stationary states in autothermic processes.^61,62^ Figure 2 shows a summary of the reactivity measured for the 12 synthesized materials under different *t*_OS_ and *T*_oven_. Materials and reaction conditions that do not significantly convert acetylene are displayed as gray cells in Figure 2. The monometallic Cu, Ag and Au catalysts at all three *T*_oven_ and the Pd_1_Au_5_, Pd_1_Au_9_ and Pd_1_Ag_9_ alloys at 50 °C hardly convert acetylene under the tested reaction conditions. In the remaining cases, displayed as colored cells in Figure 2, acetylene is fully converted (*X*_C_2_H_2__ ≥ 0.99) at least for a certain period of time on stream. In general, we observe minor changes of activity with *t*_OS_. However, in few situations these changes are remarkable, for instance due to the deactivation in the case of the Pd_1_Cu_9_ material at *T*_oven_ = 50 °C at ca. *t*_OS_ = 160 min (Figure 2A).

**Figure 2:**
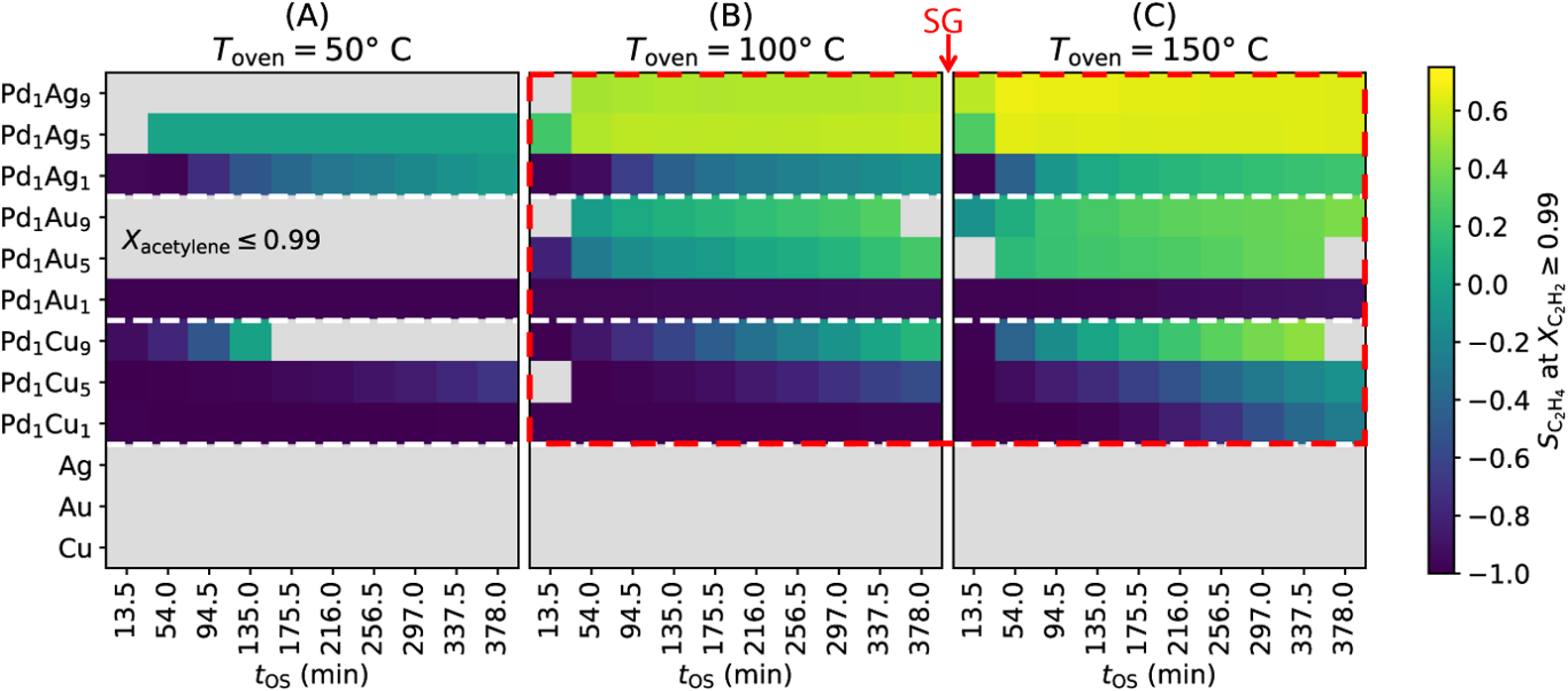
Summary of selectivity towards ethylene (*S*_C_2_H_4__) measured on 12 materials (monometallics and Pd–Ag, Pd–Au, and Pd–Cu alloys) under different reaction temperatures (*T*_oven_ = 50, 100, and 150^◦^C) and times on stream (*t*_OS_) during the selective hydrogenation of concentrated acetylene streams. Panels (A), (B), and (C) show results for the three temperatures of the oven surrounding the reactor (*T*_oven_). The colored cells reflect the *S*_C_2_H_4__ values for situations corresponding to full acetylene conversion (*X*_C_2_H_2__ ≥ 0.99). The gray cells indicate situations corresponding to lower acetylene conversion (*X*_C_2_H_2__ ≤ 0.99). In most cases, the gray cells correspond to *X*_C_2_H_2__≈ 0. The materials and *T*_oven_ corresponding to the identified SG (vide infra) are indicated by the red box. Reaction conditions: applied feed of C_2_H_2_:C_2_H_4_:H_2_ 1:1:5, weight hourly space velocity of 90,000 cm^3^ h^–1^ g_cat_^–1^, 10 bar pressure.

The catalytic behavior of the active materials in terms of *S*_C_2_H_4__ ranges from nonselective to highly selective. In some cases even the cofed ethylene is fully hydrogenated to ethane, resulting in negative *S*_C_2_H_4__ values of up to −1.00 (dark blue cells in Figure 2). The highly selective situations present *S*_C_2_H_4__ values of up to 0.75 (lime cells Figure 2). *S*_C_2_H_4__ increases with the dilution of palladium in the second metal in the order: copper < gold < silver. This trend can be understood based on (geometric) site-isolation and electronic-perturbation effects. These effects lead to weaker adsorption of ethylene compared to the pure palladium system, thus favoring its desorption over further hydrogenation to ethane.^59,63–65^ While our results are in line with reports on the selective hydrogenation of acetylene at lower concentrations, much higher ethylene selectivities (e.g., *S*_C_2_H_4__ > 0.90) at full acetylene conversion have been reported for similar bimetallic catalysts at significantly lower acetylene concentrations (≤1 vol %), inert gas dilution (>40 vol %) and atmospheric pressure.^39,66,67^ These milder conditions provide overall better reaction control with respect to the ones used in our study. The ethylene selectivity at the lower acetylene concentrations is mainly reduced by the full hydrogenation side reaction (selectivity toward C_2+_ products <0.03).^39,66,67^ Under our reaction conditions, however, the higher surface coverage of acetylene significantly reduces the ethylene selectivity due to the hydrooligomerisation side reaction (selectivity toward C_2+_ products up to 0.23).^41^ Therefore, high exothermicity and increased hydrooligomerization tendency at higher acetylene concentrations make it more challenging to achieve the desired selective behavior.

**Table 1:**
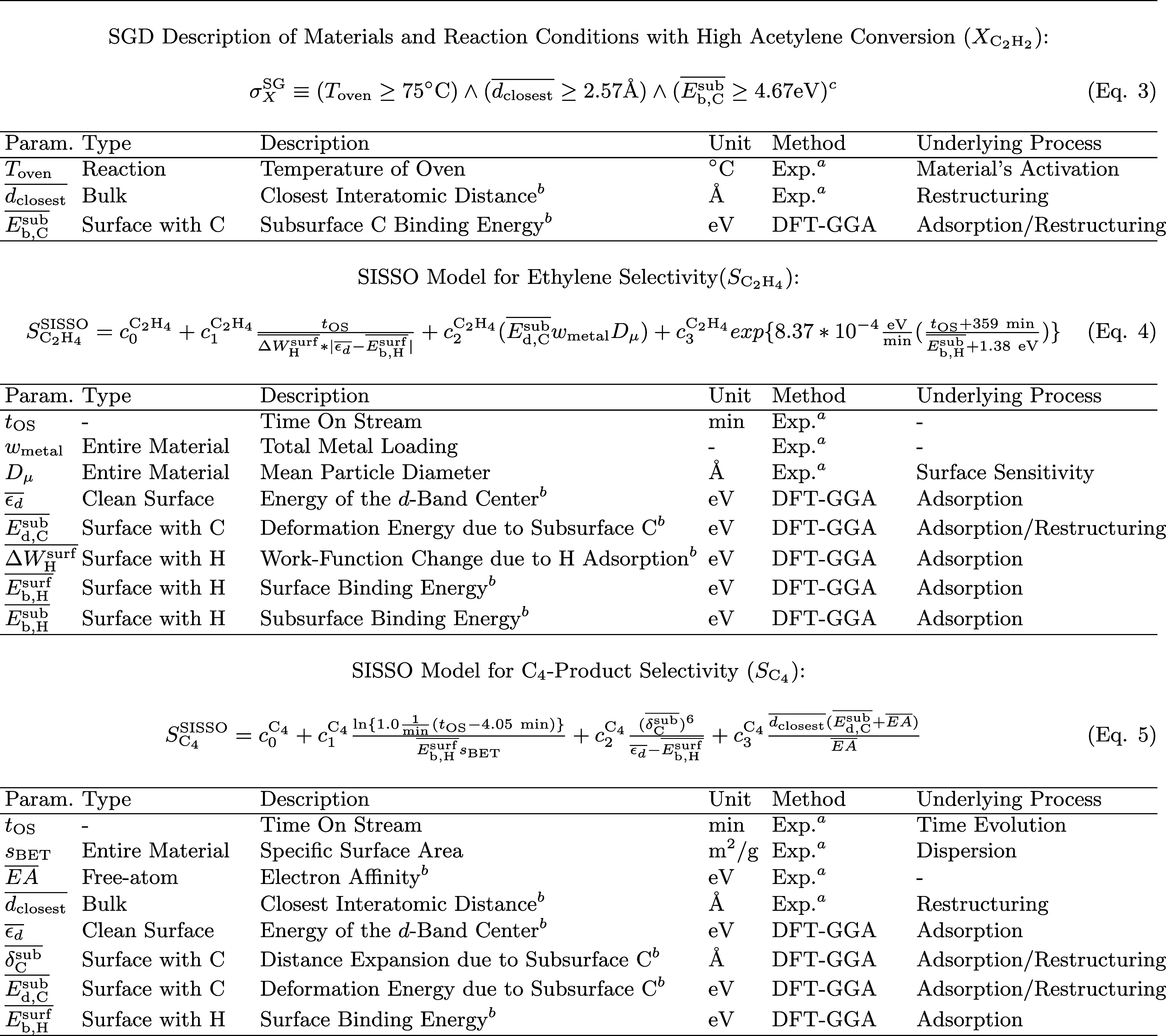
AI Analysis of the Selective Hydrogenation of Concentrate Acetylene Streams: Identified Models, Key Parameters and Associated Underlying Processes

a Measured experimental parameter.

b Composition-average.

c The symbol ∧ corresponds to the ”AND” operator.

We also analyzed the influence of reaction temperature and time-on-stream on the ethylene selectivity, highlighted in Figure 2. *S*_C_2_H_4__ increases significantly with *T*_oven_ for most of the materials. While this observation seems counterintuitive, it is most likely due to accelerated desorption phenomena and lowered surface coverage of all involved species (hydrogen, acetylene, ethylene)^68,69^ with increasing *T*_oven_. Regarding the time-on-stream evolution of the selectivity toward ethylene, we observe different behaviors depending on the composition of the catalyst material. Most of the Pd–Ag alloys are selective from the start of the reaction. For these materials, the selectivity reaches a stable level and then slightly decreases at longer *t*_OS_. The Pd_1_Ag_9_ alloy, for instance, presents *S*_C_2_H_4__ = 0.75 (*S*_C_2_H_6__ = 0.20 and *S*_C_4__ = 0.07)at *T*_oven_= 150 °C, leveling off to $S_{C_{2}H_{4}}=0.65$ (*S*_C_2_H_6__ = 0.14 and *S*_C_4__ = 0.08) after 365 min on stream at constant full acetylene conversion. The loss of ethylene selectivity with time on stream for the Pd–Ag alloys is most likely linked to the surface segregation of palladium, a common issue of alloyed hydrogenation catalysts, especially under the quite harsh reaction conditions (7 bar, H_2_ partial pressure) applied in this study.^26,40,38,70,71^ In contrast, Pd–Au and especially Pd–Cu alloys present constantly increasing *S*_C_2_H_4__ with *t*_OS_. The Pd_1_Au_9_ and Pd_1_Cu_9_ alloys, for example, are initially nonselective with *S*_C_2_H_4__ = −0.12­(*S*_C_2_H_6__ = 0.94 and *S*_C_4__ = 0.11) and *S*_C_2_H_4__ = −0.89(*S*_C_2_H_6__ = 1.73 and *S*_C_4__ = 0.10), respectively, at *T*_oven_= 150 °C. Then, the ethylene selectivity gradually increases with time on stream to *S*_C_2_H_4__ = 0.40 (*S*_C_2_H_6__ = 0.21 and *S*_C_4__ = 0.14) and *S*_C_2_H_4__ = 0.47 (*S*_C_2_H_6__ = 0.21 and *S*_C_4__ = 0.13), respectively, after 365 min at constant full acetylene conversion. While ethane selectivity behaves inversely with respect to the ethylene selectivity, the selectivity toward C_4_ products increases with the addition of a second metal (silver < copper < gold) and with *t*_OS_, but it decreases with increasing temperature. Overall, similar selectivity trends with respect to *t*_OS_ for similar metal elements in the alloy suggest a element-specific (sub)­surface modification during *t*_OS_.

In order to identify physical parameters correlated with the underlying processes controlling the *t*_OS_-dependent selectivity under comparable conversions, we will focus our AI approach, described in the next sections, on materials and reaction conditions providing stable full acetylene conversion levels. We note that this situation is not ideal in sequential hydrogenation reactions such as the acetylene hydrogenation reaction.

### 3.2 Identification of Subgroups of Materials and Reaction Conditions Resulting in Noticeable Acetylene Conversion

We begin our AI analysis using SGD to identify materials descriptions and reaction conditions that provide significant acetylene conversion. These SGs are designed to pinpoint a narrow distribution of the target variable *X*_C_2_H_2__. The data set used in the SGD analysis contains 1,076 data points, corresponding to the measured *X*_C_2_H_2__ of the 12 materials tested under three *T*_oven_ and associated with *t*_OS_ values between 0 and 405 min. The identified SG contains 539 data points, ca. 50% of the data set, corresponding to most of the materials and reaction conditions associated with high, close-to-one *X*_C_2_H_2__ values (see the details of SGD analysis in ESI). Such a narrow, nonzero conversion range will enable a proper comparison of selectivity across materials and reaction conditions (see next section). The SG contains all the bimetallic Pd–Ag, Pd–Au, and Pd–Cu alloys. The inactive monometallic systems Ag, Au, and Cu are not part of the SG. This SG is described by the rules σ^SG^_*X*_, displayed in eq 3 of Table 1. The identified rules constrain the values of three descriptive parameters: *T*_oven_, the temperature of the oven in which the reactor is placed, $\overset{―}{d_{\mathrm{closest}}}$ , the composition-averaged closest interatomic bulk distance, and $\overset{―}{E_{b,C}^{\mathrm{sub}}}$ , the composition-averaged binding energy of subsurface carbon.

The constraint in *T*_oven_ reflects that a minimum reaction temperature is required to activate the material for the catalytic reaction. The relevance of the parameters $\overset{―}{d_{\mathrm{closest}}}$ and $\overset{―}{E_{b,C}^{\mathrm{sub}}}$ indicates that bulk as well as surface properties of the NPs, in particular the subsurface interaction with carbon, are related to conversion. The direct correlation between $\overset{―}{E_{b,C}^{\mathrm{sub}}}$ and activity is somehow unexpected as the adsorption energies of acetylene, ethylene and hydrogen (dissociative) on clean surfaces of noble metals like palladium and on Pd–Ag alloys are highly exothermic (−82 to −172 kJ/mol) and almost barrierless.^68,72,73,74,75^ Thus, such metallic surfaces are expected to be highly active for acetylene hydrogenation in the absence of surface or subsurface carbon. It is possible, however, that the presence of subsurface carbon reduces the binding strength of surface species that otherwise block the materials’ surface and hinder the reaction. Indeed, surfaces of palladium and Pd–Ag alloys are expected to be covered by species formed from activated acetylene during its hydrogenation, e.g., vinyl and ethylidyne, due to the higher binding strength of acetylene (adsorption energy of −172 kJ/mol) compared to that of hydrogen (adsorption energy of −82 kJ/mol). Thus, there is a competitive adsorption of acetylene that blocks active sites for the dissociative hydrogen adsorption, which is reflected by negative exponents of acetylene partial pressures in kinetic models for hydrogenation activity.^68,72–75^ Besides, subsurface carbide structures adsorb acetylene (and ethylene) less strongly^26,59,60,76–78^ than the metallic counterparts, which would lead to an increased availability of palladium (stabilized low-coordinated sites) and thus increased hydrogenation activity. Particularly under the concentrations (and surface coverage) of acetylene applied in this study, it is likely that a pronounced restructuring of the surface and subsurface relying on bulk properties (e.g., sufficiently sized interstitial sites for carbon) takes place. The formation of stable active surface sites for the dissociative adsorption of hydrogen via the on-stream interaction of the subsurface with (hydro-)­carbons might then play a more important role for the hydrogenation activity then the actual interaction properties of the clean surface towards hydrogen.

The 12 materials of the data set are represented in the coordinates of the key materials-dependent parameters identified by SGD $(\overset{―}{d_{\mathrm{closest}}}$ and $\overset{―}{E_{b,C}^{\mathrm{sub}}})$ in Figure 3A. Note that the parameters in the axes of this graph were selected by SGD out of the 21 offered candidate descriptive parameters. In this plot, the bimetallic alloys, which are part of the SG, are shown as red circles. The monometallic systems, which are not part of the SG, are shown as black circles. The SG rules are represented by the dashed red lines and arrows, and the SG region is highlighted in red.

**Figure 3:**
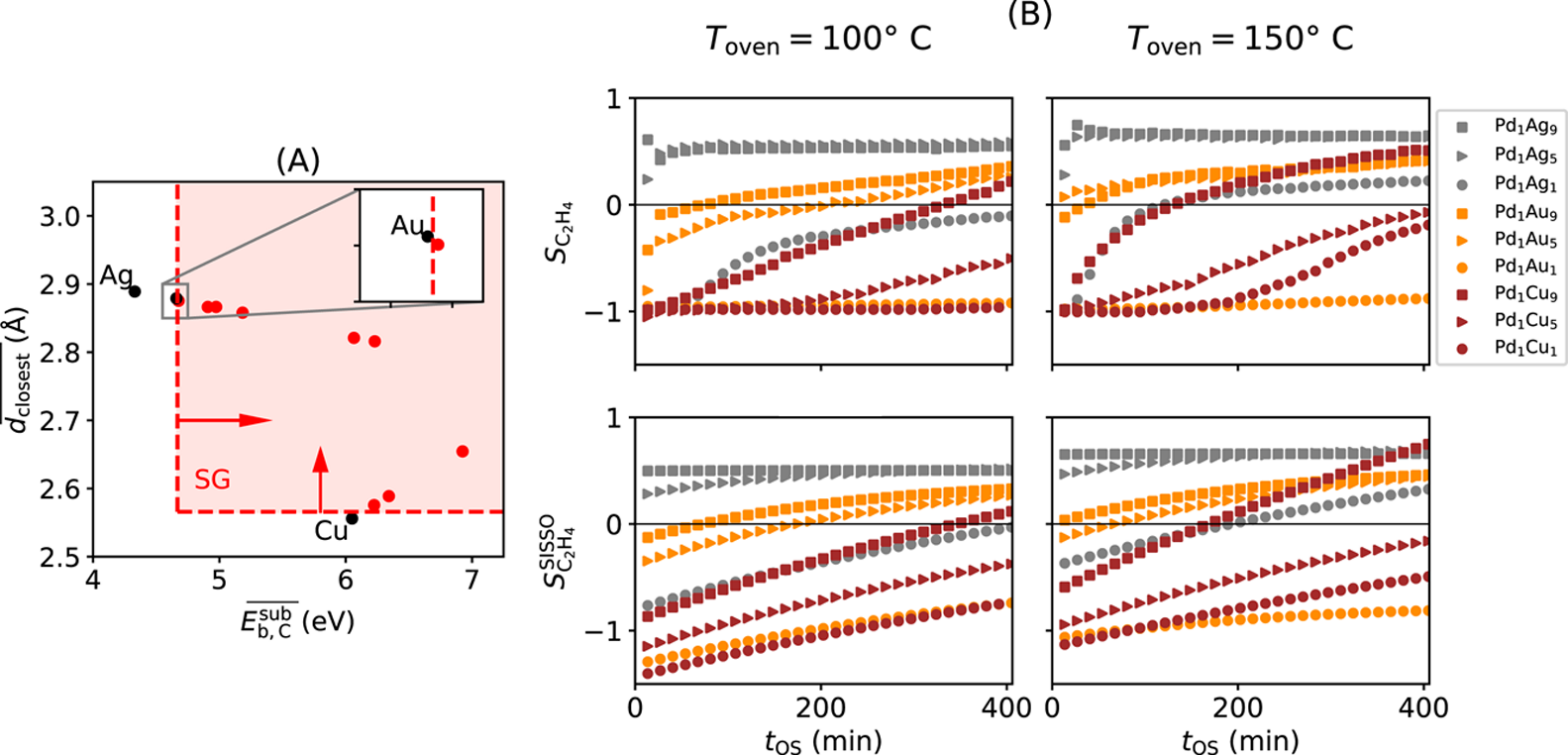
AI modeling of ethylene selectivity during the selective hydrogenation of concentrated acetylene streams. (A): Identification of subgroup (SG) rules describing materials and reaction conditions resulting in high acetylene conversion. (B): Measured (top row) and SISSO-modeled (bottom row) selectivity towards ethylene formation (*S*_C_2___H_4__, eq 4). The two columns display results at different oven temperatures *T*_oven_ 100 and 150 °C, respectively. *S*_C_2___H_4__ < 0 indicates that more ethylene (from the feed) was consumed than formed from acetylene. *S*_C_2___H_4__ > 0 indicate that more ethylene was formed from acetylene than consumed (selective materials and conditions).

In this application, our data set contains either situations resulting in near-zero conversion or situations resulting in near-one conversions, and the identified SG corresponds to the latter. However, SGD can be used to identify descriptions of materials and reaction conditions associated with moderate or low activity. This is important because highly active materials and conditions are not necessarily those displaying the desired selectivity, e.g., in consecutive reactions.^12,14^

Finally, we stress that by manually selecting data points associated with *X*_C_2_H_2__ ≈ 1, one could obtain similar data sets compared to the data sets identified by SGD. However, and importantly, the manual approach would not provide the rules describing such data sets. Thus, SGD not only identifies the subsets of data, but it provides the rules of eq 3 in Table 1. These rules, in particular the constraints $\overset{―}{d_{\mathrm{closest}}}\geq 2.57Å$ and $\overset{―}{E_{b,C}^{\mathrm{sub}}}\geq 4.67\mathrm{eV}$ , can be used to indicate whether a new material, i.e., a material that was not synthesized or tested yet, provides noticeable acetylene conversion. The application of these rules and their usefulness will be illustrated in Figure 4A.

**Figure 4:**
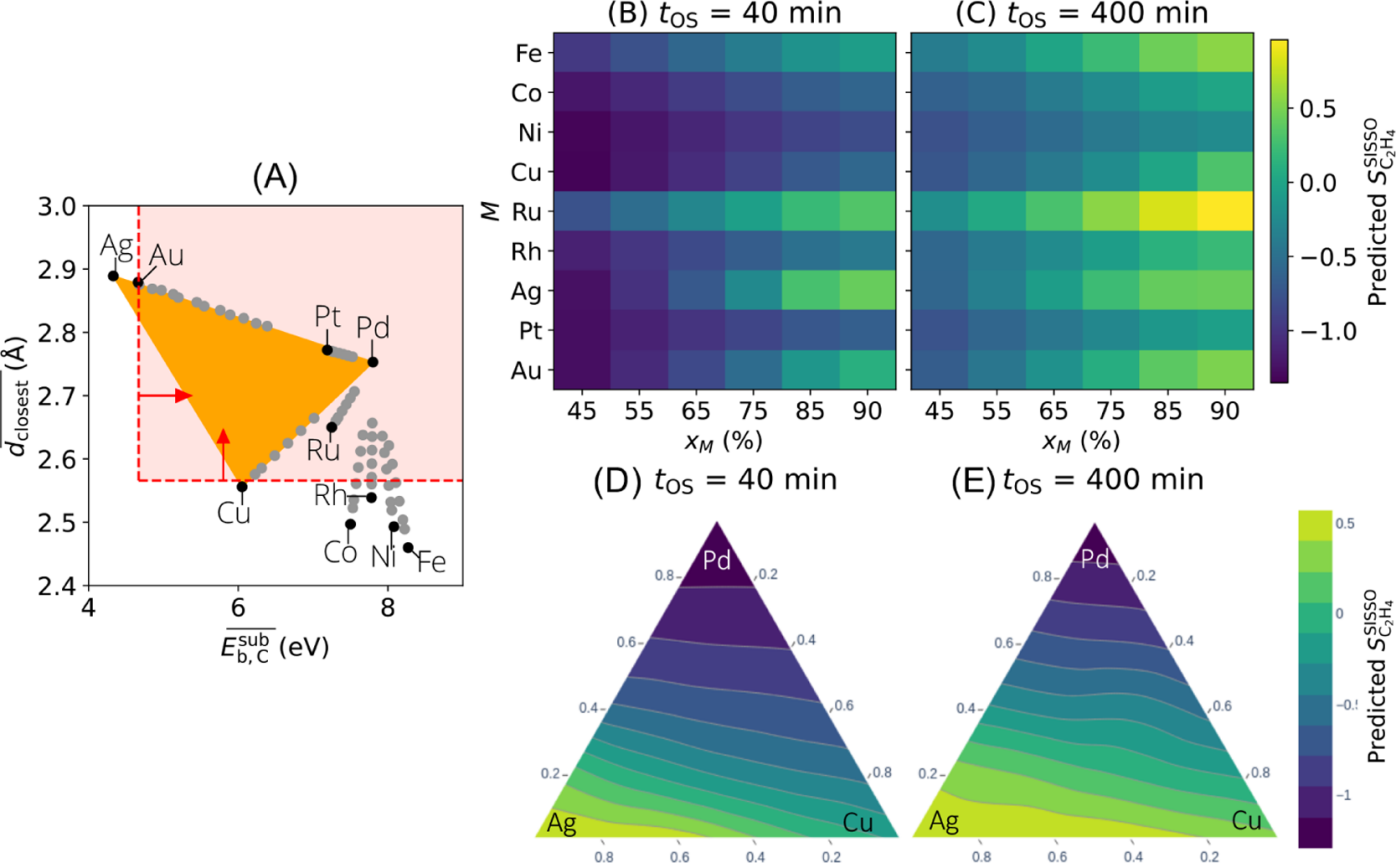
SG rules and SISSO models derived based on palladium-, silver-, gold-, and copper-based materials synthesized by ball milling are used to design bi- and trimetallic palladium-based systems for the catalytic hydrogenation of concentrated acetylene. (A): The SG rules on composition-averaged closest interatomic distance $\left(\overset{―}{d_{\mathrm{closest}}}\right)$ and subsurface carbon binding energy $\left(\overset{―}{E_{b,C}^{\mathrm{sub}}}\right)$ identify alloy compositions that fully convert acetylene. (B) and (C): Ethylene selectivity predicted by the SISSO model $\left(S_{C_{2}H_{4}}^{\mathrm{SISSO}}\right)$ for bimetallic systems (Pd_(1–x_M_)_ M_x_M__, with *M* = Fe, Co, Ni, Cu, Ru, Rh, Ag, Pt, Au) at two *t*_OS_s. (D) and (E): Ethylene selectivity predicted quantitatively by the SISSO model $\left(S_{C_{2}H_{4}}^{\mathrm{SISSO}}\right)$ for trimetallic systems Pd_*x*_Ag_*y*_Cu_1‑*x*‑*y*_ at two *t*_OS_’s. The selectivity predictions are made for *T*_oven_ = 150 ^◦^C.

### 3.3 Identification of Symbolic-Regression Models for Time-Dependent Selectivity Focused on Noticeable Acetylene Conversion

Let us now analyze the selectivity of the materials and reaction conditions that present high activity and belong to the identified SG. The evolution of measured *S*_C_2_H_4__ values with *t*_OS_ for the materials in the SG are shown in Figure 3B (upper panels) for *T*_oven_= 100 and 150 °C. This figure shows that the induction period might last, in the case of some materials such as the Pd–Cu alloys, up to hours. This suggests that the compound initially placed in the reactor is undergoing significant modifications at this time scale.

By using only the data points belonging to the SG associated with high acetylene conversion (539 data points), we trained SISSO models for *S*_C_2_H_4__. The cross-validation analysis (see details in ESI) shows that the identified models describe unseen data with good accuracy. The fit of the best SISSO models to the data, shown in Figure 3B (bottom panels) indicates that the identified expression (eq 4 in Table 1) is able to capture the selectivity trends both across materials and with *t*_OS_. In particular, the selectivity shift of the Pd_1_Cu_9_ material (Figure 3B, square brown markers) with *t*_OS_ is well captured by the SISSO model.

In eq 4, the coefficients correspond to different fitted values for the two different oven temperatures (see ESI for details). Thus, the temperature dependency of the selectivity is captured by these coefficients.

First, we note that the model expression contains the key parameter *t*_OS_, required to describe the ethylene selectivity time dependency. The total metal loading (*w*_metal_) and mean value of particle size (*D*_μ_), measured prior to the catalytic test, are identified as key experimental parameters. Finally, the following materials-dependent theoretical key parameters appear in eq 4: the *d*-band center $\left(\overset{―}{\epsilon_{d}}\right)$ , the deformation energy of subsurface carbon $\left(\overset{―}{E_{d,C}^{\mathrm{sub}}}\right)$ , the work-function shift with hydrogen adsorption $\left(\overset{―}{\Delta W_{H}^{\mathrm{surf}}}\right)$ , the binding energy of hydrogen $\left(\overset{―}{E_{b,H}^{\mathrm{surf}}}\right)$ , and the binding energy of subsurface hydrogen $\left(\overset{―}{E_{b,H}^{\mathrm{sub}}}\right)$ .

The presence of the microscopic parameters $\overset{―}{\epsilon_{d}}$ , $\overset{―}{E_{d,C}^{\mathrm{sub}}}$ , $\overset{―}{\Delta W_{H}^{\mathrm{surf}}}$ , $\overset{―}{E_{b,H}^{\mathrm{surf}}}$ , and $\overset{―}{E_{b,H}^{\mathrm{sub}}}$ highlights the importance of surface properties. These parameters reflect the interaction of surface and subsurface with carbon and hydrogen and its crucial role for the selectivity towards ethylene. In particular $\overset{―}{\epsilon_{d}}$ , $\overset{―}{\Delta W_{H}^{\mathrm{surf}}}$ and $\overset{―}{E_{b,H}^{\mathrm{surf}}}$ can be related to the adsorption behavior of the alloy surface towards acetylene, ethylene and hydrogen. A downshift of $\overset{―}{\epsilon_{d}}$ away from the Fermi level due to alloy formation in palladium weakens, e.g., the adsorption of π-bonded species such as ethylene, thus favoring its desorption over overhydrogenation.^79,80^ $\overset{―}{E_{b,H}^{\mathrm{surf}}}$ describes the interaction of the surface with hydrogen in terms of bond strength, whereas $\overset{―}{\Delta W_{H}^{\mathrm{surf}}}$ reflects the electronic effect of hydrogen adsorption on palladium states and also correlates with adsorption properties. Thus, the presence of the parameters $\overset{―}{\epsilon_{d}}$ , $\overset{―}{E_{b,H}^{\mathrm{surf}}}$ , and $\overset{―}{\Delta W_{H}^{\mathrm{surf}}}$ in eq 4 could be associated with the balance between the adsorption of acetylene/ethylene and hydrogen that controls ethylene selectivity.^81^ The relevance of the parameter $\overset{―}{E_{d,C}^{\mathrm{sub}}}$ suggests the formation of metal carbide structures in the subsurface. Indeed, carbide formation can enhance the ethylene selectivity of palladium-based catalysts by weakening the adsorption of ethylene and thereby lowering the risk of overhydrogenation.^26,59,76,78,82,83^ Finally, the parameters *w*_metal_ and *D*_μ_ could be related to a particle size effect on the ethylene selectivity. For instance, the size of palladium or copper NPs can significantly affect their selectivity in the hydrogenation of unsaturated bonds by controlling the relative amount of corner and edge sites, which are coordinatively less saturated and can thus provide stronger adsorption geometries than the surface sites on closely packed flat surfaces.^60,84,85^ Thus, *w*_metal_ and *D*_μ_ could be associated with surface-sensitivity. Interestingly, the *t*_OS_-independent term of eq 4 contains the parameters $\overset{―}{E_{d,C}^{\mathrm{sub}}}$ , *w*_metal_, and *D*_μ_, whereas the *t*_OS_-dependent terms of eq 4 contain the parameters $\overset{―}{\epsilon_{d}}$ , $\overset{―}{\Delta W_{H}^{\mathrm{surf}}}$ , $\overset{―}{E_{b,H}^{\mathrm{surf}}}$ , and $\overset{―}{E_{b,H}^{\mathrm{sub}}}$ . This suggests that the time-on-stream behavior of the investigated alloys is mainly determined by the interaction of the materials’ surface with hydrogen and likely to the availability of surface hydrogen. The higher impact of time on the reactivity, which increases as Pd–Ag < Pd–Au < Pd–Cu can thus be rationalized based on the affinity of the metal surfaces for hydrogen, which increases in the order silver < gold < copper. This results in higher importance of the time-dependent terms of eq 4 for the Pd–Cu alloys. Surface hydrides, if present in our systems, are expected to form on a short time scale.^86^ In particular, such time scale is likely shorter compared to that for the formation of surface carbides. Thus, the change of availability with time indicated by the SISSO model of eq 4 are not likely related with the formation of surface hydrides.

We have also trained SISSO models for the selectivity towards ethane. These results are discussed in the ESI. In addition to ethylene and ethane, C_4_ products such as butane, 1-butene, 2-cis-butene, 2-trans-butene, and 1,3-butadiene are formed during the acetylene hydrogenation in particular at elevated acetylene concentrations. The selectivity toward C_4_ products is lower than the selectivity toward ethylene or ethane *S*_C_4__ < 0.2. However, the formation of C_4_ products indicates the tendency of carbon–carbon bond formation, and thus the tendency of forming acetylene oligomers, often referred to as *green oil*. These oligomers can accumulate on the surface of the material leading to the loss of activity (deactivation) typically at long *t*_OS_.^40^ In order to get insights on the processes that lead to oligomer formation and thus to catalyst deactivation, we also trained a SISSO model for *S*_C_4__ by using the 539 data points belonging to the SG associated with high acetylene conversion. This model is noted in eq 5 of Table 1 (see further details in ESI).

The key parameters identified in eq 5 are *t*_OS_, specific surface area (*s*_BET_), composition-averaged electron affinity $\left(\overset{―}{EA}\right)$ , $\overset{―}{d_{\mathrm{closest}}}$ , $\overset{―}{\epsilon_{d}}$ , composition-averaged distance expansion due to subsurface carbon $\left(\overset{―}{\delta_{C}^{\mathrm{sub}}}\right)$ , $\overset{―}{E_{d,C}^{\mathrm{sub}}}$ , and $\overset{―}{E_{b,H}^{\mathrm{surf}}}$ . The relevance of the parameters $\overset{―}{\epsilon_{d}}$ , $\overset{―}{E_{b,H}^{\mathrm{surf}}}$ , $\overset{―}{E_{d,C}^{\mathrm{sub}}}$ and $\overset{―}{\delta_{C}^{\mathrm{sub}}}$ points at the role of adsorption properties, in particular related to acetylene/ethylene and hydrogen (dissociation), in controlling the selectivity to oligomeric species. Indeed, surface sites that provide strong palladium–carbon binding are more prone to oligomerize acetylene and other surface intermediates during the reaction.^71^ The parameters $\overset{―}{E_{d,C}^{\mathrm{sub}}}$ and $\overset{―}{\delta_{C}^{\mathrm{sub}}}$ might also correlate with surface restructuring. The relevance of the bulk parameters $\overset{―}{d_{\mathrm{closest}}}$ and $\overset{―}{EA}$ , in turn, could be related to the materials’ restructuring under the reaction, e.g., via dealloying.^38,70^ The dealloying of palladium-based materials results in the creation of palladium-rich surface sites, which could more efficiently oligomerize acetylene compared to sites containing both palladium and other metals such as silver.^71^ Indeed, the X-ray diffraction (XRD) analysis of some of the palladium–silver alloys after the reaction^40^ indicates the segregation of palladium to the surface. This shows that the structure of the material initially placed inside the reactor is likely modified during the reaction. In case of a fixed particle size, *s*_BET_ is correlated with the distances between NPs. The relevance of this parameter could point at the importance of average distances between particles in the oligomerization side reaction. However, the average particle sizes in the investigated data set vary between 3 to 9 nm. Thus, this interpretation has to be taken with care. Interestingly, the time-dependent term of eq 5, which captures the *S*_C_4__ increase with *t*_OS_, contains the parameters $\overset{―}{E_{b,H}^{\mathrm{surf}}}$ and *s*_BET_. Thus, the availability of hydrogen as well as underlying processes related to the specific surface area modulate the evolution of the formation of C_4_ molecules over time and are likely associated with catalyst deactivation.

When comparing the SISSO models for ethylene and C_4_ selectivities (eqs 4 and 5 of Table 1, respectively) we note that the theoretical surface-related parameters *t*_OS_, $\overset{―}{\epsilon_{d}}$ , $\overset{―}{E_{b,H}^{\mathrm{surf}}}$ , and $\overset{―}{E_{d,C}^{\mathrm{sub}}}$ appear in both equations. This is consistent with the fact that the surface intermediates to ethylene formation (acetylene, vinyl and vinylidene) are also precursors for the carbon–carbon coupling to oligomeric species that similarly undergo a hydrogenation of their unsaturated bonds (e.g., vinylacetylene, 1,3-butadiene, butene, and butane). Additionally, the parameter $\overset{―}{d_{\mathrm{closest}}}$ is present both in the description of active materials and reaction conditions (eq 3 and eq 5). This suggests that structure sensitivity of oligomer formation might orginate from the fact that carbon–carbon couplings of the above-mentioned surface species necessitates several palladium atoms (or metals with strong carbon affinity) in close proximity to each other on the surface in order to allow for palladium–carbon binding throughout the coupling process.^64,71^ The affinity to carbon is indeed increasing in the order Ag < Au < Cu < Pd, explaining the observed selectivity differences to C_4_ compounds.

We like to stress that the AI models derived in this work depend on a combination of several parameters in a nonlinear fashion. Therefore, by assigning a too specific, chemical meaning to each parameter individually, one might overlook the possibly intricate interplay of many processes governing catalysis. Moreover, the discussion on the relevance of the identified key parameters is based on the current knowledge about the systems. It is possible that other, so far not well understood or unknown underlying processes, e.g., the dynamic restructuring of the catalyst during the reaction, play an important role. The correlations derived by AI might capture these processes. In fact, the AI description does not necessarily reflect causality. Thus, the physical relationships between the identified key parameters and the underlying chemistry might be indirect.

### 3.4 Exploitation of the AI Models to Design Bimetallic and Trimetallic Systems and Experimental Verification

Let us now exploit the SGD and SISSO models trained using a data set containing palladium alloys with silver, gold, and copper to design bimetallic systems containing a wider range of stoichiometries as well as other chemical elements. We focus on the hypothetical materials Pd_(1–*x_M_*)_ *M*_*x_M_*_, where *M* = Fe, Co, Ni, Cu, Ru, Rh, Ag, Pt, Au. Note that not all of these hypothetical stoichiometries might result in solid solutions. Besides, not all of these materials might be synthesizable via the mechanochemical approach. Values of *x*_*M*_in the range [50,90] % are analyzed. The bimetallic materials with *x*_*M*_ 45, 55, 65, 75, 85, 90% are represented as gray circles in the $\overset{―}{d_{\mathrm{closest}}}$ vs $\overset{―}{E_{b,C}^{\mathrm{sub}}}$ plot of Figure 4A. In this plot, the monometallic systems are represented as black circles and the SG rules describing materials associated with high acetylene conversion are displayed as red dashed lines. The red shaded area corresponds to the materials predicted to have high acetylene conversion. The systems containing platinum and ruthenium are predicted to fully convert acetylene irrespective of *x*_*M*_. This is consistent with the fact that these metals are traditional hydrogenation catalysts.^38,87^ Conversely, according to the SG rules, the systems based on iron, cobalt, nickel, copper, rhodium, silver, and gold only present high acetylene conversion below a certain *x_M_* value, i.e., the amount of palladium has to be higher than a certain threshold. While nickel and rhodium are reported to be active for the hydrogenation of diluted acetylene,^32,39^ they have not yet been tested in the high-concentration, high-pressure conditions in order to verify these predictions.

The ethylene selectivity values for the bimetallic systems Pd_(1–*x_M_*)_ *M_x_M__* were predicted based on eq 4. The color scale in the (*x*_*M*_,*M*) composition matrix of Figure 4B,C reflects the $S_{C_{2}H_{4}}^{\mathrm{SISSO}}$ values at *T*_oven_ = 150°C for *t*_OS_ 40 and 400 min, respectively. For *t*_OS_ = 40 min, the materials containing high amounts of ruthenium, silver, and gold present $S_{C_{2}H_{4}}^{\mathrm{SISSO}}>0$ . For *t*_OS_ = 400 min, the ethylene selectivity increases, and some compositions with high *x*_*M*_ based on iron, cobalt, and copper also reach $S_{C_{2}H_{4}}^{\mathrm{SISSO}}>0$ . However, iron-, cobalt-, copper-, silver-, and gold-based materials with high *x*_*M*_ do not satisfy the SG rules for high *X*_C_2_H_2__ shown in Figure 4A. Thus, the AI models indicate that bimetallic systems based on these metals could be selective at the expense of a lower (possibly close-to-zero) activity. Among the considered bimetallic systems, the ruthenium-based materials with high *x_M_* are predicted to be the most selective ones. For instance, the bimetallic material with *x*_Ru_ = 90% is associated with $S_{C_{2}H_{4}}^{\mathrm{SISSO}}=0.95$ for *t*_OS_ = 400 min. This material is also predicted to be highly active according to the SG rules shown in Figure 4A. However, Ru–Pd systems are not expected to be miscible at any composition according to the phase diagram.^88^ Alternatively, the SISSO model indicates that silver-based materials with *x*_*M*_ higher than 90% might be even more selective than the material with the highest selectivity in the training set, Pd_1_Ag_9_.

In order to test the predictions of the AI models, we synthesized, characterized by TEM and SEM-EDX, and tested in catalysis the new Pd_1_Ag_12_ and Pd_1_Ag_15_ bimetallic materials. The acetylene conversion $\left(X_{C_{2}H_{2}}^{\mathrm{measured}}\right)$ measured at *T*_oven_ = 150 °C at different *t*_OS_, shown in Figure 5A,B, indicates full conversion of acetylene and is consistent with the analysis of the SG rules in Figure 4A, which indicates that the activity for these systems only drops at very low amounts of palladium. The measured and predicted ethylene selectivity ( $S_{C_{2}H_{4}}^{\mathrm{measured}}$ and $S_{C_{2}H_{4}}^{\mathrm{SISSO}}$ , respectively) are shown in Figure 5A,B as blue and red squares. The values of *D*_μ_ are 6.03 and 7.04 nm and the values of *w*_metal_ are 0.0153 and 0.0161 for the synthesized Pd_1_Ag_12_ and Pd_1_Ag_15_ materials, respectively. Pd_1_Ag_12_ and Pd_1_Ag_15_ present an initial measured selectivity of 67 and 68%, respectively, at 27 min. These selectivities slightly drop to 61% after 405 min on stream. These values are comparable to the selectivity of the material Pd_1_Ag_9_ (65% at 405 min), which was the material with the best performance among those used to train the model. The SISSO model predicts the ethylene selectivity for Pd_1_Ag_12_ and Pd_1_Ag_15_ with good accuracy, in spite of the deviations observed in particular for Pd_1_Ag_15_. Additionally, the predictions correctly indicate that Pd_1_Ag_15_ is more selective than Pd_1_Ag_12_. Such good predictive performance is expected, since the compositions of these materials are only slightly different from that of the Pd_1_Ag_9_ material, which was included in the training set. The SISSO model is also correct in that no significant change in selectivity occurs with *t*_OS_. Indeed, only minor decreases in selectivity are observed in experiment.

**Figure 5:**
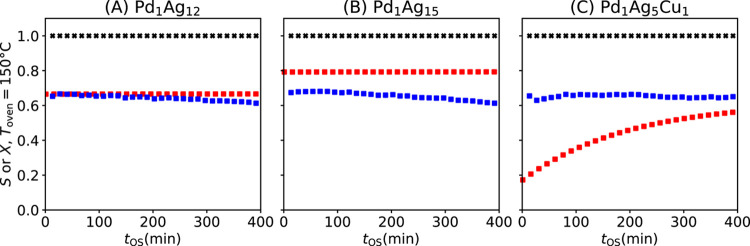
Reactivity of two bimetallic materials (A) Pd_1_Ag_12_ and (B) Pd_1_Ag_15_ and one trimetallic material (C) Pd_1_Ag_5_Cu_1_ suggested by the AI models was measured. The measured ethylene selectivity $S_{C_{2}H_{4}}^{\mathrm{measured}}$ (blue squares) is compared with the predictions of the SISSO model $S_{C_{2}H_{4}}^{\mathrm{SISSO}}$ (red squared). The measurements and predictions are made for *T*_oven_ = 150 °C. The black crosses indicate $X_{C_{2}H_{2}}^{\mathrm{measured}}$ . Reaction conditions: applied feed of C_2_H_2_:C_2_H_4_:H_2_ 1:1:5, weight hourly space velocity of 90,000 cm^3^ h^–1^ $g_{\mathrm{cat}}^{-1}$ , 10 bar pressure.

In addition to the bimetallic systems, we also used the AI models to predict the reactivity of the Pd–Ag–Cu system as examples of trimetallic materials. This system is represented as an orange triangle in Figure 4A. The SG constraints indicate that high $X_{C_{2}H_{2}}$ can be achieved for most of the compositions, with the exception of silver- and copper-rich systems. The predicted $S_{C_{2}H_{4}}^{\mathrm{SISSO}}$ values (Figure 4D,E) indicate that the selectivity increases with *t*_OS_, in particular for silver- and copper-rich compositions. The composition for which the selectivity presents its maximum value at *t*_OS_ = 400 min is approximately Pd_1_Ag_5_Cu_1_. The trimetallic Pd_1_Ag_5_Cu_1_ system was experimentally synthesized, characterized by TEM and EDX-SEM, and tested in catalysis. The measured *D*_μ_ and *w*_metal_ for Pd_1_Ag_5_Cu_1_ is equal to 5.80 nm and 0.0145, respectively. It presents an initial measured selectivity of 62% at 27 min, which slightly increases to 65% at 405 min (Figure 5C). Although the trimetallic Pd_1_Ag_5_Cu_1_ material is overall only as selective as the most selective material of the training set (Pd_1_Ag_9_), it successfully combines the beneficial characteristics of the Pd–Ag (high initial selectivity) and Pd–Cu alloys (selectivity improvement with time on stream). The SISSO model correctly predicts in a qualitative manner, that the ethylene selectivity of Pd_1_Ag_5_Cu_1_ increases with time. Besides, the selectivity of this material at long *t*_OS_, e.g., *t*_OS_ > 300 min, is predicted quantitatively by SISSO. However, the predicted selectivity values deviate significantly from the measurements at the beginning of the reaction. This suggests that different processes might govern the initial selectivity of the trimetallic system than those governing the bimetallic materials in the training data set. These results indicate the potential and limitations of our AI approach, i.e., of the offered primary features and operators, to model the reactivity and guide the design of materials.

In this study, we trained AI models based on 12 materials and confirmed that these models have a good overall predictive performance for 3 new materials. These results indicate that the models obtained by SGD and SISSO are generalizable to some extent. Thus, the cross-validation strategy for selecting SISSO model hyperparameters and the SGD objective function mitigated overfitting, which could be severe for models trained based on such a small number of materials. However, we stress that AI will only provide reliable descriptions of materials whose reactivity is governed by the same underlying physical processes that govern the reactivity of the materials in the training set. In order to accurately describe materials that are significantly different compared to the materials in the training set, whose reactivity might be also governed by different physical processes, the AI model might need to be retrained with more data. For instance, the quality of the predictions of SISSO models can be improved with more data acquired in a systematic way using active learning.^89^ By training ensembles of SISSO models with different subsets of training data or with different subsets of primary features, a statistical distribution of analytical expressions can be created. These expressions all describe the training data well. However, the predictions of the different expressions for new materials present a variance, which quantifies the uncertainty of the predictions. This uncertainty can be used to steer the acquisition of new data in unexplored regions of the materials space. Thus, larger portions of materials space can be systematically covered. Furthermore, the active-learning framework can also focus the acquisition of new data corresponding to the high-performance scenarios to improve the reliability of the SISSO description for these most important cases.

## 4 Conclusions

We developed and applied a focused AI approach to model the time-on-stream evolution of the measured reactivity of palladium-based alloys synthesized mechanochemically and applied in the selective hydrogenation of equally concentrated acetylene-ethylene streams. Out of 21 experimental and theoretical candidate descriptive parameters reflecting the reaction conditions and materials’ mesoscopic, bulk, and (sub)­surface properties, AI identified key physical parameters correlated with the reactivity. These key parameters highlight the crucial influence of surface and subsurface carbon and hydrogen chemistry on the activity and selectivity, among other underlying processes. Selected parameters in direct correlation with the time on stream furthermore suggest element-specific (sub)­surface modifications on stream leading to different selectivity behavior with increasing time on stream. The AI models suggested modifications of the bimetallic Pd–Ag systems and indicated a promising trimetallic Pd–Ag–Cu system. The proposed materials were tested experimentally. Even though they present overall comparable ethylene selectivities as the best performing catalysts in the training set, the evolution of their reactivity with time on stream, influenced by up to two different additional metal elements, was predicted with good accuracy. The *focused AI approach* also allowed to narrow down the materials space where good catalysts can be found and it suggested additional, new promising materials that should be investigated. Further investigations center currently on the mechanochemical synthesis of immiscible Pd–Ru alloys, which would be a promising and new material (with respect to the elements entering the composition) predicted by the AI models to outperform the Pd–Ag systems. In fact, mechanochemistry might enable the synthesis of immiscible systems, e.g., Pd–Pt and Pt–Au.^47^ The focused data-centric approach introduced in this paper allows to navigate across immense pools of possible catalytic materials while taking into account their time-on-stream behavior, making it a valuable tool in catalyst and process design.

## Supplementary Material





## Notes

The ESI contains additional details on the experiments, data set, and SGD and SISSO AI approaches. It also provides details on the exploitation of AI models for materials design and the SISSO model for ethane selectivity­(PDF)
The data set is provided in the data set-acetylene.xls file (XLSX)

## References

[1] Somorjai G. A., Park J. Y. (2008). Molecular factors of catalytic selectivity. Angew. Chem. Int. Ed..

[2] Thomas J. M. (2008). Heterogeneous
catalysis: Enigmas, illusions, challenges, realities, and emergent
strategies of design. J. Chem. Phys..

[3] Freund H.-J., Meijer G., Scheffler M., Schlögl R., Wolf M. (2011). CO oxidation as a prototypical reaction for heterogeneous processes. Angew. Chem. Int. Ed..

[4] Schlögl R. (2015). Heterogeneous
catalysis. Angew. Chem. Int. Ed..

[5] Mou L.-H., Han T. T., Smith P. E. S., Sharman E., Jiang J. (2023). Machine learning
descriptors for data-driven catalysis study. Adv. Sci..

[6] Zhao Z.-J., Gong J. (2022). Catalyst design via
descriptors. Nat. Nanotechnol..

[7] Takahashi K., Takahashi L., Le S. D., Kinoshita T., Nishimura S., Ohyama J. (2022). Synthesis of heterogeneous catalysts
in catalyst informatics to bridge experiment and high-throughput calculation. J. Am. Chem. Soc..

[8] Taniike T., Fujiwara A., Nakanowatari S., García-Escobar F., Takahashi K. (2024). Automatic
feature engineering for catalyst design using
small data without prior knowledge of target catalysis. Commun. Chem..

[9] Esterhuizen J. A., Goldsmith B. R., Linic S. (2022). Interpretable machine learning for
knowledge generation in heterogeneous catalysis. Nat. Catal..

[10] Medford A. J., Vojvodic A., Hummelshøj J. S., Voss J., Abild-Pedersen F., Studt F., Bligaard T., Nilsson A., Nørskov J. K. (2015). From the
sabatier principle to a predictive theory of transition-metal heterogeneous
catalysis. J. Catal..

[11] Trunschke A., Bellini G., Boniface M., Carey S. J., Dong J., Erdem E., Foppa L., Frandsen W., Geske M., Ghiringhelli L. M. (2020). Towards experimental handbooks in catalysis. Top. Catal..

[12] Foppa L., Ghiringhelli L. M., Girgsdies F., Hashagen M., Kube P., Hävecker M., Carey S. J., Tarasov A., Kraus P., Rosowski F., Schlögl R., Trunschke A., Scheffler M. (2021). Materials genes of heterogeneous catalysis from clean
experiments and artificial intelligence. MRS
Bull..

[13] Foppa L., Sutton C., Ghiringhelli L. M., De S., Löser P., Schunk S. A., Schäfer A., Scheffler M. (2022). Learning design
rules for selective oxidation catalysts from high-throughput experimentation
and artificial intelligence. ACS Catal..

[14] Foppa L., Rüther F., Geske M., Koch G., Girgsdies F., Kube P., Carey S. J., Hävecker M., Timpe O., Tarasov A. V. (2023). Data-centric heterogeneous
catalysis: identifying rules and materials genes of alkane selective
oxidation. J. Am. Chem. Soc..

[15] Bellini G., Koch G., Girgsdies F., Dong J., Carey S. J., Timpe O., Auffermann G., Scheffler M., Schlögl R., Foppa L. (2025). CO oxidation
catalyzed
by perovskites: The role of crystallographic distortions highlighted
by systematic experiments and artificial intelligence. Angew. Chem. Int. Ed..

[16] Miyazaki R., Belthle K. S., Tüysüz H., Foppa L., Scheffler M. (2024). Materials genes of CO_2_ hydrogenation on
supported cobalt catalysts: An artificial intelligence approach integrating
theoretical and experimental data. J. Am. Chem.
Soc..

[17] Sutton C., Boley M., Ghiringhelli L. M., Rupp M., Vreeken J., Scheffler M. (2020). Identifying domains of applicability of machine learning
models for materials science. Nat. Commun..

[18] Wrobel, S. An algorithm for multi-relational discovery of subgroups. European Conference On Principles Of Data Mining And Knowledge Discovery; Springer, 1997. DOI: 10.1007/3-540-63223-9_108.

[19] Atzmueller M. (2015). Subgroup discovery. WIREs Data
Min. Knowl. Discovery.

[20] Goldsmith B. R., Boley M., Vreeken J., Scheffler M., Ghiringhelli L. M. (2017). Uncovering structure-property relationships of materials
by subgroup discovery. New J. Phys..

[21] Foppa L., Scheffler M. (2025). Coherent collections
of rules describing exceptional
materials identified with a multi-objective optimization of subgroups. Digit. Discov..

[22] Ouyang R., Curtarolo S., Ahmetcik E., Scheffler M., Ghiringhelli L. M. (2018). SISSO: A compressed-sensing method for identifying
the best low-dimensional descriptor in an immensity of offered candidates. Phys. Rev. Mater..

[23] Purcell T. A. R., Scheffler M., Carbogno C., Ghiringhelli L. M. (2022). SISSO++: A C++ implementation
of the sure-independence screening
and sparsifying operator approach. J. Open Source
Softw..

[24] Trotuş I.-T., Zimmermann T., Schüth F. (2014). Catalytic reactions of acetylene:
A feedstock for the chemical industry revisited. Chem. Rev..

[25] Bowker M., DeBeer S., Dummer N. F., Hutchings G. J., Scheffler M., Schüth F., Taylor S. H., Tüysüz H. (2022). Advancing
critical chemical processes for a sustainable future: Challenges for
industry and the Max Planck–Cardiff centre on the Fundamentals
of Heterogeneous Catalysis (FUNCAT). Angew.
Chem. Int. Ed..

[26] Li Z., Öztuna E., Skorupska K., Vinogradova O. V., Jamshaid A., Steigert A., Rohner C., Dimitrakopoulou M., Prieto M. J., Kunkel C. (2024). Rationally designed
laterally-condensed-catalysts deliver robust activity and selectivity
for ethylene production in acetylene hydrogenation. Nat. Commun..

[27] Delikonstantis E., Igos E., Augustinus M., Benetto E., Stefanidis G. D. (2020). Life cycle
assessment of plasma-assisted ethylene production from rich-in-methane
gas streams. Sustain. Energy Fuels..

[28] Pässler, P. ; Hefner, W. ; Buckl, K. ; Meinass, H. ; Meiswinkel, A. ; Wernicke, H.-J. ; Ebersberg, G. ; Müller, R. ; Bässler, J. ; Behringer, H. , ;Acetylene; Wiley-VCH Verlag GmbH & Co. KGaA John Wiley & Sons, Ltd, 2007, 10.1002/14356007.a01_097.pub4.

[29] Cao X., Jang B. W.-L., Hu J., Wang L., Zhang S. (2023). Synthetic
strategies of supported Pd-based bimetallic catalysts for selective
semi-hydrogenation of acetylene: A review and perspectives. Molecules.

[30] Salciccioli M., Chen Y., Vlachos D. G. (2011). Microkinetic
modeling and reduced
rate expressions of ethylene hydrogenation and ethane hydrogenolysis
on platinum. Ind. Eng. Chem. Res..

[31] Dasgupta A., He H., Gong R., Shang S.-L., Zimmerer E. K., Meyer R. J., Liu Z.-K., Janik M. J., Rioux R. M. (2022). Atomic control of
active-site ensembles in ordered alloys to enhance hydrogenation selectivity. Nat. Chem..

[32] McCue A. J., Anderson J. A. (2015). Recent advances in selective acetylene
hydrogenation
using palladium containing catalysts. Front.
Chem. Sci. Eng..

[33] Pei G. X., Liu X. Y., Wang A., Lee A. F., Isaacs M. A., Li L., Pan X., Yang X., Wang X., Tai Z., Wilson K., Zhang T. (2015). Ag alloyed
Pd single-atom catalysts
for efficient selective hydrogenation of acetylene to ethylene in
excess ethylene. ACS Catal..

[34] Albani D., Capdevila-Cortada M., Vilé G., Mitchell S., Martin O., López N., Pérez-Ramírez J. (2017). Semihydrogenation
of acetylene on indium oxide: Proposed single-ensemble catalysis. Angew. Chem. Int. Ed..

[35] Pei G. X., Liu X. Y., Yang X., Zhang L., Wang A., Li L., Wang H., Wang X., Zhang T. (2017). Performance of Cu-alloyed
Pd single-atom catalyst for semihydrogenation of acetylene under simulated
front-end conditions. ACS Catal..

[36] Tejeda-Serrano M., Cabrero-Antonino J. R., Mainar-Ruiz V., López-Haro M., Hernández-Garrido J. C., Calvino J. J., Leyva-Pérez A., Corma A. (2017). Synthesis of supported
planar iron oxide nanoparticles and their
chemo- and stereoselectivity for hydrogenation of alkynes. ACS Catal..

[37] Ball M. R., Rivera-Dones K. R., Gilcher E. B., Ausman S. F., Hullfish C. W., Lebrón E. A., Dumesic J. A. (2020). AgPd and CuPd catalysts for selective
hydrogenation of acetylene. ACS Catal..

[38] Zhang L., Zhou M., Wang A., Zhang T. (2020). Selective hydrogenation
over supported metal catalysts: From nanoparticles to single atoms. Chem. Rev..

[39] Xie K., Xu K., Liu M., Song X., Xu S., Si H. (2023). Catalysts
for selective hydrogenation of acetylene: a review. Mater. Today Catal..

[40] Kley K. S., De Bellis J., Schüth F. (2023). Selective hydrogenation of highly
concentrated acetylene streams over mechanochemically synthesized
PdAg supported catalysts. Catal. Sci. Technol..

[41] Mauß J. M., Schüth F. (2025). On the role
of anions in solid catalysts with ionic
liquid layer (SCILL) for the selective hydrogenation of highly concentrated
acetylene streams. ChemSusChem.

[42] Schreyer H., Eckert R., Immohr S., de Bellis J., Felderhoff M., Schüth F. (2019). Milling down to nanometers: A general
process for the direct dry synthesis of supported metal catalysts. Angew. Chem. Int. Ed..

[43] De
Bellis J., Felderhoff M., Schüth F. (2021). Mechanochemical
synthesis of supported bimetallic catalysts. Chem. Mater..

[44] Amrute A. P., De Bellis J., Felderhoff M., Schüth F. (2021). Mechanochemical
synthesis of catalytic materials. Chem. Eur.
J..

[45] Ardila
Fierro K. J., Hernández J. G. (2021). Sustainability assessment of mechanochemistry
by using the twelve principles of green chemistry. ChemSusChem.

[46] Amrute A. P., Łodziana Z., Schreyer H., Weidenthaler C., Schüth F. (2019). High-surface-area
corundum by mechanochemically induced
phase transformation of boehmite. Science.

[47] De
Bellis J., Petersen H., Ternieden J., Pfänder N., Weidenthaler C., Schüth F. (2022). Direct dry
synthesis of supported bimetallic catalysts: A study on comminution
and alloying of metal nanoparticles. Angew.
Chem. Int. Ed..

[48] Trotuş, I.-T. Catalytic Conversion Of Acetylene To Butadiene And Butenes; Ruhr-Universität Bochum, 2017.

[49] Trotuş I.-T., Zimmermann T., Duyckaerts N., Geboers J., Schüth F. (2015). Butadiene
from acetylene–ethylene cross-metathesis. Chem. Commun..

[50] Agbaba Ö., Trotuş I.-T., Schmidt W., Schüth F. (2023). Light olefins
from acetylene under pressurized conditions. Ind. Eng. Chem. Res..

[51] Ouyang R., Ahmetcik E., Carbogno C., Scheffler M., Ghiringhelli L. M. (2019). Simultaneous learning of several
materials properties
from incomplete databases with multi-task SISSO. J. Phys. Mater..

[52] Mazheika A., Geske M., Müller M., Schunk S. A., Rosowski F., Kraehnert R. (2024). Data-driven
design of catalytic materials in methane
oxidation based on a site isolation concept. ACS Catal..

[53] Han Z.-K., Sarker D., Ouyang R., Mazheika A., Gao Y., Levchenko S. V. (2021). Single-atom alloy catalysts designed by first-principles
calculations and artificial intelligence. Nat.
Commun..

[54] Xu L., Wang X., Hu X., Wang Y., Zhang C., Xu W., Zhao W., Xu N., Woo D., Yao H., Li X., Jiang H., Huang M., Lee J., Zeng X. C., Han Z.-K. (2025). Artificial-intelligence-assisted
design principle for
developing high-performance single-atom catalysts. Innovation.

[55] Hammer B., Nørskov J. K. (2000). Theoretical
surface science and catalysis–calculations
and concepts. Adv. Catal..

[56] Greeley J., Mavrikakis M. (2005). Surface and
subsurface hydrogen: Adsorption properties
on transition metals and near-surface alloys. J. Phys. Chem. B.

[57] Sautet P., Cinquini F. (2010). Surface of metallic catalysts under a pressure of hydrocarbon
molecules: Metal or carbide?. ChemCatChem.

[58] Teschner D., Borsodi J., Wootsch A., Révay Z., Hävecker M., Knop-Gericke A., Jackson S. D., Schlögl R. (2008). The roles
of subsurface carbon and hydrogen in palladium-catalyzed alkyne hydrogenation. Science.

[59] Studt F., Abild-Pedersen F., Bligaard T., Sørensen R. Z., Christensen C. H., Nørskov J. K. (2008). On the role of surface modifications
of palladium catalysts in the selective hydrogenation of acetylene. Angew. Chem. Int. Ed..

[60] Yang B., Burch R., Hardacre C., Headdock G., Hu P. (2013). Influence
of surface structures, subsurface carbon and hydrogen, and surface
alloying on the activity and selectivity of acetylene hydrogenation
on Pd surfaces: A density functional theory study. J. Catal..

[61] van
Heerden C. (1953). Autothermic processes. Ind. Eng.
Chem..

[62] van
Heerden C. (1958). The character of the stationary state of exothermic
processes. Chem. Eng. Sci..

[63] Wang L., Li F., Chen Y., Chen J. (2019). Selective hydrogenation of acetylene
on SiO_2_-supported Ni-Ga alloy and intermetallic compound. J. Energy Chem..

[64] Kuhn M., Lucas M., Claus P. (2015). Long-time stability
vs deactivation
of Pd–Ag/Al_2_O_3_ egg-shell catalysts in
selective hydrogenation of acetylene. Ind. Eng.
Chem. Res..

[65] Studt F., Abild-Pedersen F., Bligaard T., Sørensen R. Z., Christensen C. H., Nørskov J. K. (2008). Identification of non-precious metal
alloy catalysts for selective hydrogenation of acetylene. Science.

[66] Chen L., Li X.-T., Ma S., Hu Y.-F., Shang C., Liu Z.-P. (2022). Highly selective
low-temperature acetylene semihydrogenation
guided by multiscale machine learning. ACS Catal..

[67] Luo Q., Wang H., Wang L., Xiao F.-S. (2022). Alloyed PdCu nanoparticles
within siliceous zeolite crystals for catalytic semihydrogenation. ACS Mater. Au.

[68] Mei D., Neurock M., Smith C. M. (2009). Hydrogenation
of acetylene–ethylene
mixtures over Pd and Pd–Ag alloys: First-principles-based kinetic
monte carlo simulations. J. Catal..

[69] Ayodele O. B., Shittu T. D. (2022). Catalysis of semihydrogenation
of acetylene to ethylene:
current trends, challenges, and outlook. Front.
Chem. Sci. Eng..

[70] Huang W., Pyrz W., Lobo R. F., Chen J. G. (2007). Selective
hydrogenation
of acetylene in the presence of ethylene on K^+^-*β*-zeolite supported Pd and PdAg catalysts. Appl. Catal., A.

[71] Vignola E., Steinmann S. N., Al Farra A., Vandegehuchte B. D., Curulla D., Sautet P. (2018). Evaluating
the risk of C–C
bond formation during selective hydrogenation of acetylene on palladium. ACS Catal..

[72] Medlin J. W., Allendorf M. D. (2003). Theoretical
study of the adsorption of acetylene on
the (111) surfaces of Pd, Pt, Ni, and Rh. J.
Phys. Chem. B.

[73] Mei D., Sheth P. A., Neurock M., Smith C. M. (2006). First-principles-based
kinetic monte carlo simulation of the selective hydrogenation of acetylene
over Pd(111). J. Catal..

[74] Aireddy D. R., Ding K. (2022). Heterolytic dissociation
of H_2_ in heterogeneous catalysis. ACS Catal..

[75] Heard C. J., Siahrostami S., Grönbeck H. (2016). Structural
and energetic trends of
ethylene hydrogenation over transition metal surfaces. J. Phys. Chem. C.

[76] Yang B., Burch R., Hardacre C., Hu P., Hughes P. (2016). Importance
of surface carbide formation on the activity and selectivity of Pd
surfaces in the selective hydrogenation of acetylene. Surf. Sci..

[77] Zhai P., Aireddy D. R., Berko M. B., Arshadi A., Zachman M. J., Cullen D. A., Xu Y., Ding K. (2025). Anomalous
role of carbon
in Pd-catalyzed selective hydrogenation. Angew.
Chem. Int. Ed..

[78] Liu Y., Fu F., McCue A., Jones W., Rao D., Feng J., He Y., Li D. (2020). Adsorbate-induced structural evolution of Pd catalyst
for selective hydrogenation of acetylene. ACS
Catal..

[79] Sheth P. A., Neurock M., Smith C. M. (2005). First-principles
analysis of the
effects of alloying Pd with Ag for the catalytic hydrogenation of
acetylene-ethylene mixtures. J. Phys. Chem.
B.

[80] Yang T., Feng Y., Ma R., Li Q., Yan H., Liu Y., He Y., Miller J. T., Li D. (2021). Improvement of selectivity
in acetylene hydrogenation with comparable activity over ordered PdCu
catalysts induced by post-treatment. ACS Appl.
Mater. Interfaces.

[81] Dong W., Ledentu V., Sautet P. H., Eichler A., Hafner J. (1998). Hydrogen adsorption
on palladium: a comparative theoretical study of different surfaces. Surf. Sci..

[82] Torres D., Cinquini F., Sautet P. (2013). Pressure and temperature
effects
on the formation of a Pd/C surface carbide: Insights into the role
of Pd/C as a selective catalytic state for the partial hydrogenation
of acetylene. J. Phys. Chem. C.

[83] Teschner D., Révay Z., Borsodi J., Hävecker M., Knop-Gericke A., Schlögl R., Milroy D., Jackson D., Torres D., Sautet P. (2008). Understanding palladium hydrogenation
catalysts: When the nature of the reactive molecule controls the nature
of the catalyst active phase. Angew. Chem. Int.
Ed..

[84] Brandt
Corstius O. E., van der Hoeven J. E.
S., Sunley G. J., de Jongh P. E. (2023). Influence of particle size in Pd-catalysed selective
hydrogenation of 1,3-butadiene. J. Catal..

[85] Totarella G., de Rijk J. W., Delannoy L., de Jongh P. E. (2022). Particle size effects
in the selective hydrogenation of alkadienes over supported Cu nanoparticles. ChemCatChem.

[86] Bloch J., Mintz M. H. (1997). Kinetics and mechanisms of metal hydrides formation–a
review. J. Alloys Compd..

[87] Liu L., Corma A. (2018). Metal catalysts for
heterogeneous catalysis: From single atoms to
nanoclusters and nanoparticles. Chem. Rev..

[88] Tripathi S. N., Bharadwaj S. R., Dharwadkar S. R. (1993). The Pd-Ru system (palladium-ruthenium). J. Phase Equilib..

[89] Nair A. S., Foppa L., Scheffler M. (2025). Materials-discovery workflow guided
by symbolic regression for identifying acid-stable oxides for electrocatalysis. npj Comput. Mater..

