# Supplementary material for: Modeling Time-On-Stream Catalyst Reactivity in the Selective Hydrogenation of Concentrated Acetylene Streams under Industrial Conditions via Experiments and AI
Source: ACS Catal. 2025 Jul 11;15(15):12652–65. doi: 10.1021/acscatal.5c02226 (PMC12322879; doi:10.1021/acscatal.5c02226)
Supplement: Supplementary file 1 [file cs5c02226_si_001.pdf]

Electronic Supplementary Information (ESI) for Modeling Time-On-Stream Catalyst Reactivity in the Selective Hydrogenation of Concentrated Acetylene Streams Under Industrial Conditions via Experiments and AI

Jonathan M. Mauß,[1] Klara S. Kley,[1] Rohini Khobragade,[1] Nguyen-Khang Tran,[1] Jacopo de Bellis,[1] Ferdi Schüth,[1] Matthias Scheffler,[2] and Lucas Foppa*[2]

[1]*Max-Planck-Institut für Kohlenforschung, Kaiser-Wilhelm-Platz 1, 45470 Mülheim an der Ruhr, Germany*
[2]*The NOMAD Laboratory at the Fritz Haber Institute of the Max Planck Society, Faradayweg 4-6, 14195 Berlin, Germany*
(Dated: July 9, 2025)

*foppa@fhi-berlin.mpg.de

I. ADDITIONAL EXPERIMENTAL DETAILS

All the syntheses steps were carried out in a planetary mill according to procedures published previously using yttria-stabilized zirconia lined ball milling equipment (5.5 wt% Y_2O_3) to avoid iron (and other potentially active metals) impurities from abrasion in the materials.[1, 2] For the synthesis of the Pd-Ag (and Pd-Au) alloys, metallic Pd and Ag (Au) powders were added to the milling jar in amounts corresponding to the target molar ratio, e.g., 1:1, 1:5, and 1:9, for the materials Pd_1Ag_1, Pd_1Ag_5, and Pd_1Ag_9 (Pd_1Au_1, Pd_1Au_5, and Pd_1Au_9), respectively. After the addition of γ-AlOOH (bohemite), the solid mixture was milled, leading to the formation of metal nanoparticles supported on HSA-α-Al_2O_3. The relative amount of metal with respect to support was kept at 5% in a weight basis (noted 5 wt%). Materials with ca. 1 wt% were obtained by diluting the 5 wt% with freshly prepared HSA-α-Al_2O_3 using the ball mill. Before characterization and catalytic tests, the synthesized materials were subjected to a thermal activation procedure consisting of a reduction step under H_2 (150 °C for 3 hours) and an annealing step under argon (Ar) atmosphere (600 °C for 10 hours). The Pd_1Ag_{12} and Pd_1Ag_{15} alloyed catalysts were prepared accordingly. For the synthesis of the Pd-Cu alloys metallic Pd and Cu powders were added to the milling jar in amounts corresponding to the target molar ratio and balled milled before the addition of γ-AlOOH in a second ball-milling step to yield metal nanoparticles supported on HAS-α-Al_2O_3. The relative amount of metal with respect to support was kept at 5% in a weight basis (noted 5 wt%). Materials with ca. 1 wt% were obtained by diluting the 5 wt% with freshly prepared HSA-α-Al_2O_3 using the ball mill. Before characterization and catalytic tests, the synthesized materials were subjected to a thermal activation procedure consisting of a reduction step (150 °C for 3 hours) and an annealing step (600 °C for 10 hours) under H_2 atmosphere. The silver-based materials (molar ratios 0:1, 1:1, 1:5 and 1:9) were reported in [3], while the copper- and gold-based materials were reported in [4]. For the synthesis of the trimetallic Pd-Ag-Cu material, metallic Pd and Cu powders were added to the milling jar in amounts corresponding to the target molar ratio and balled milled (15 min). Then, Ag was added in the target molar ratio and the solid mixture was ball-milled a second time (30 min) before the addition of γ-AlOOH in a third ball-milling step to yield metal nanoparticles supported on HSA-α-Al_2O_3. The relative amount of metal with respect to support was kept at 5 wt%. Materials with ca. 1 wt% were obtained by diluting the 5 wt% with freshly prepared HSA-α-Al_2O_3 using the ball mill. Before characterization and catalytic tests, the synthesized materials were subjected to a thermal activation procedure consisting of a reduction step under H_2 (150 °C for 3 hours, 600 °C, for 2 hours) and an annealing step under argon (Ar) atmosphere (600 °C for 8 hours). The materials were carefully analyzed via N_2 physisorption, PXRD, XPS, HR-TEM, HAADF-STEM, TEM-EDX, SEM and SEM-EDX. Further details on the synthesis procedures and characterization can be found in [3, 4] and in the supplementary file dataset-acetylene.xlsx.

For the catalytic tests, 100 mg of catalyst (pelletized and sieved to a 300-400 μm fraction) was mixed with 3.0 g silicon carbide (46 grit) as inert dilutant and placed onto a sieve inside a fixed bed flow reactor tube (8 mm i.d., stainless steel) and stabilized with a quartz wool plug on top. Hydrogen, ethylene and methane were used as purchased while acetylene was extracted and purified from acetone (alumina/zeolite A extrudates) and compressed in-house to 25 bar (two-stage compressor). Methane was only added to the product stream after the reactor and used as internal standard for the quantification of the reaction products (via peak area ratio) by gas chromatography (Agilent 7890B, three sequential columns (detector type): Rxi-5Sil MS (FID), RT-Alumina BOND/Na_2SO_4 (FID) and RT-Msieve 5A (TCD)). To avoid the condensation of possibly high-boiling acetlyene oligomers all tubing downstream of the reactor was heated to 150 °C. The gas flows were set to 100 mL min^{-1} for hydrogen, 20 mL min^{-1} for ethylene and acetylene, and 10 mL min^{-1} for methane using previously calibrated mass flow controllers. The catalysts were tested at a total weight hourly space velocity of 90 000 cm^3 h^{-1} g_{cat}^{-1} and 10 bar pressure. The reactor was heated to 50, 100 or 150 °C via an external oven equipped with a thermocouple. The temperature inside the reactor was measured with a second thermocouple immersed in the catalyst bed.

The selectivity towards ethane ($S_{C_2H_6}$) was calculated

as

$$S_{C_2H_6} = \frac{n_{C_2H_6,out}}{n_{C_2H_2,in} - n_{C_2H_2,out}}. \quad (S1)$$

In Eq. S1, $n_{C_2H_6,out}$ is molar flow rate of ethane leaving the reactor. The values of $S_{C_2H_6}$ range from 0 to 2. $S_{C_2H_6} = 2$ indicates that all acetylene and all ethylene was converted to ethane (total hydrogenation).

The selectivity towards C_4 hydrocarbons (S_{C_4}) was calculated as

$$S_{C_4} = \frac{2n_{C_4,out}}{n_{C_2H_2,in} - n_{C_2H_2,out}}. \quad (S2)$$

In Eq. S2, $n_{C_4,out}$ is molar flow rate of the C_4 products butane, 1-butene, 2-cis-butene, and 2-trans-butene at the reactor outlet, as determined by gas chromatography. The C_4 product 1,3-butadiene was not considered, as the selectivity towards this product is estimated from screening experiments to be well below 1%.

We started measuring the reactivity measured at the time-on-stream t_{OS} value of 13.5 minutes (min). Then, the measurements were performed at each 13.5 min (corresponding to the analysis time of one GC run) up to t_{OS} values of 972 min for some materials and applied temperature of oven (T_{oven}). However, the measurements were interrupted at earlier t_{OS} if the acetylene conversion reached (near) zero. This can happen due to deactivation of a catalyst that converted acetylene at low t_{OS} or due to a catalyst that was inactive since the beginning of the measurements. We focus our AI analysis on the initial stages of the reaction with $t_{OS} < 405$ min. Further details on materials' synthesis and their testing in the hydrogenation of concentrated acetylene streams are available in a previous publication.[3] The reactor setup is described in more details elsewhere.[5–8]

Due to the explosive nature of acetylene, stringent safety measures (e.g. explosion-safe cubicle, redundant check valves, remote control of valves, MFC, etc.) and specialized equipment are essential for safe experimentation. Further details on the safety structure of the reactor setup in general or the catalytic testing in the hydrogenation of concentrated acetylene streams are available in previous publications.[3–9]

II. ADDITIONAL DETAILS ON THE DATASET

In our AI analysis, we focus on the evolution of the reactivity at initial stages of the reaction. For most of the catalysts and applied temperatures, the changes in reactivity occur in the initial 400 min. Thus, we only considered the data points with $t_{OS} < 406$ min in our analysis. The interruption of measurements when the acetylene conversion is observed to be close to zero for extended times (vide supra) results in an unbalanced dataset, since information on inactive or deactivated materials is not present in the dataset for large t_{OS}. In order to circumvent this issue, we extended the dataset for larger t_{OS} and reaction conditions for the materials Ag, Au, Cu (all T_{oven}) and for the materials Pd_1Ag_9, Pd_1Au_9 and Pd_1Ag_5 (at $T_{oven} = 50°C$), assuming that the conversion and all selectivity values remain zero. The so-obtained reactivity profiles are shown in Fig. S10 to S20. Additionally, we excluded the data points associated selectivity values significantly outside the physical ranges. We excluded 33 data points that meet the following conditions: $S_{C_2H_4} > 1.1, S_{C_2H_4} < -1.1, S_{C_2H_6} < -0.1, S_{C_2H_6} > -2.1$. The final dataset used in the AI approach contains 1,076 data points.

The full list of candidate descriptive parameters (primary features) applied in the AI analysis is shown in Table SI, along with their units and the references from which they were obtained.

The composition averages of candidate descriptive parameters are taken as

$$\overline{\phi} = \sum x_i \phi_i. \quad (S3)$$

In Eq. S3, ϕ is a property associated to one metal element and x_i are the nominal molar fractions of each metal in the materials' composition, e.g., $x_{Pd} = 1/10$ and $x_{Ag} = 9/10$ for the Pd_1Ag_9 material. The dataset utilized in the AI analysis, containing the measured reactivity and the candidate descriptive parameters for all materials, is provided in supplementary file dataset-acetylene.xlsx.

We note that the oxide support can influence the reactivity of the metal catalysts in acetylene hydrogenation.[10–12] Even though all materials considered in this work are based on the HSA-α-Al_2O_3 support, the effect of metal-support interaction can be captured by the candidate descriptive parameters indirectly, e.g., via the consideration of the specific surface area, which reflects the metal dispersion, or via the mean and standard deviation of the particle size distribution, which reflect the extent of metal-oxide interactions.

III. ADDITIONAL DETAILS ON THE SGD APPROACH

The SGD approach[13, 14] identifies partitions of the data space associated with outstanding distributions of a given target of interest, here the acetylene conversion. SGD starts by systematically creating statements about the candidate descriptive parameters that are only satisfied by a portion of the dataset. Such statements are, for instance, inequalities constraining the values of the descriptive parameters to some minimum or maximum thresholds ν to be determined during the analysis. The construction of these statements follows different approaches depending on the type of features: categorical, ordinal, and metric. For metric features, i.e., when the feature values from a continuous ordered scale, inequalities of the type $\phi \geq \nu$ and $\phi \leq \nu$ are constructed, where ϕ are candidate descriptive parameters. k-means clustering is applied to identify a computationally feasible

set of ν. Then, SGD uses a search algorithm to identify conjunctions of statements that result in subselections of data (SGs) that maximize an objective function. This objective function specifies how outstanding the SGs are with respect to the entire dataset based on their sizes and on the distributions of the target values in the SG and in the entire dataset. Here, we use the objective function

$$Q(SG, \widetilde{P}) = \frac{s(SG)}{s(\widetilde{P})} * \frac{X_\sigma(SG) - X_\sigma(\widetilde{P})}{X_\sigma(\widetilde{P})}, \quad \text{(S4)}$$

where $s(SG)$ and $s(\widetilde{P})$ are the sizes of the SG and of the entire dataset $\widetilde{P}$, respectively, and $X_\sigma(SG)$ and $X_\sigma(P)$ are the standard deviation of the distribution of the target in the SG and in the entire dataset, respectively. The first term $\frac{s(SG)}{s(\widetilde{P})}$ prevents the identification of too small and stastistically irrelevant SGs. This term also ensures that the identified SG descriptions are not too specific. Thus, SGD provides generalizable descriptions and mitigates the risk of overfitting, which is significant for datasets containing a small number of materials. Indeed, in previous works we have shown how the descriptions identified by SGD are remarkably generalizable, even when trained using a moderate number of materials or data points.[15–17]

The so-called *standard-deviation-reduction* utility function,

$$u(SG, \widetilde{P}) = \frac{X_\sigma(SG) - X_\sigma(\widetilde{P})}{X_\sigma(\widetilde{P})}, \quad \text{(S5)}$$

favors the identification of SGs associated with narrow distributions of target values. This utility function does not request that the SGs present specific target values, for instance higher or lower with respect to the average values in the entire dataset. The utility function of S5 only favors the identification of SGs with narrow distributions of target values, in our case narrow distribution of $X_{C_2H_2}$. However, we verified that the SG that maximizes the quality function correspond to $X_{C_2H_2}$ values close to 1.00. Other objective functions can be used in SGD depending on the characteristics of the target-values distribution that are interesting for a given problem.[18] The outcome of the SGD analysis is the description of the SG that maximizes the objective function. This description constrains the values of only few, key parameters, out of the many initially offered ones and might be referred to as *SG rules*. Importantly, the SG description focuses on the behavior of the data points that belong to the SG. In this sense, the SG rules are local, not global. We used the SGD approach[13, 14] as implemented in the realkd version 0.7.2. The propositions were created based on 10 different thresholds per candidate descriptive parameter. A Monte-Carlo-based SG search algorithm[19, 20] was used with 50,000 seeds for initialization. This algorithm randomly generates conjunctions of the previously generated statements with probability proportional to $\frac{s(SG)}{s(\widetilde{P})}$. Then, an opportunistic pruning algorithm refines these conjunctions by removing statements that result in the increase of $Q(SG, \widetilde{P})$ values. The iterative removal of statements leads to the maximization of the objective function. Additional details on the SGD approach are described in references[15, 16, 18].

In our dataset, the majority of situations associated with noticeable activity correspond to $X_{C_2H_2}$ values close to 1.0. This is shown in Fig. S1A, which displays an histogram reflecting the distribution of all $X_{C_2H_2}$ values in the dataset. These values correspond to all 12 catalysts measured at 3 temperatures and at all time-on-stream values. The histogram shows that the values of $X_{C_2H_2}$ are either close to zero, or close to one, with very few situations in between (see inset of Fig. S1A, which zooms in on low number of counts). Removing data points with $X_{C_2H_2} = 0$ and $S = 0$ from the SISSO modeling of selectivities prevents that the uninteresting yet numerous situations associated to zero activity dominate the selection of models for selectivity, as the root-mean-squared loss (objective) function of SISSO reflects the average performance across the entire training data. Thus, the loss function would be strongly affected by the data points with $X_{C_2H_2} = 0$ and $S = 0$.

The SG discussed in the main text and defined by Eq. 3 misses some of the situations resulting in high conversion, as indicated by the black bar of the histogram close to the value of 1.00 in Fig. S1A. These data points are associated to the materials Pd_1Ag_1, Pd_1Ag_5, Pd_1Au_1, Pd_1Cu_1, Pd_1Cu_5, and Pd_1Cu_9, which fully convert acetylene not only at T_{oven} of 100 or 150 °C, but also at $T_{oven} = 50$°C. However, lifting the constraint $T_{oven} \geq 75$°C in the rules of Eq. 3 would result in a SG containing $X_{C_2H_2} = 0$, as the materials Pd_1Ag_9, Pd_1Au_5, and Pd_1Au_9 do not convert acetylene at $T_{oven} = 50$°C. A SG containing data points associated with zero conversion would not be appropriate for the second step of the AI approach (SISSO modelling of selectivity), as the selectivity of different materials can only be fairly compared at similar conversion levels.

We note that the constraints describing the SG that maximizes the quality function, shown in Eq. 3 of the main text, are not unique because some of the candidate descriptive parameters are correlated. The analysis of linear correlations between candidate descriptive parameters is shown in Fig. S2. For instance, the SG discussed in the main text can also be described by the following rules:

$$\sigma^{SG}_{X'} \equiv T_{oven} \geq 75\ ^\circ\mathrm{C} \wedge \overline{E^{sub}_{d,C}} \leq 0.33\ \mathrm{eV} \wedge \overline{E^{sub}_{b,C}} \geq 4.68\ \mathrm{eV}, \quad \text{(S6)}$$

$$\sigma^{SG}_{X''} \equiv T_{oven} \geq 100\ ^\circ\mathrm{C} \wedge \overline{EN} \geq 1.93 \wedge \overline{d_{closest}} \leq 2.8754\ \text{Å}, \quad \text{(S7)}$$

and

$$\sigma^{\mathrm{SG}}_{X'''} \equiv T_{\mathrm{oven}} \geq 100\ ^{\circ}\mathrm{C} \wedge \overline{E^{\mathrm{sub}}_{\mathrm{b,C}}} \geq 4.67\ \mathrm{eV} \wedge \overline{EN} \geq 1.912. \tag{S8}$$

Similarly to Eq. 3 of the main text, Eq. S6 constrains the values of the parameters T_{oven} and $\overline{E^{\mathrm{sub}}_{\mathrm{b,C}}}$ to minimum values. However, while Eq. 3 presents a third constraint on the minimum value for the parameter $\overline{d_{\mathrm{closest}}}$, Eq. S6 has an additional constraint on a maximum value for the parameter $\overline{E^{\mathrm{sub}}_{\mathrm{d,C}}}$. Indeed, this reflect the fact that $\overline{d_{\mathrm{closest}}}$ and $\overline{E^{\mathrm{sub}}_{\mathrm{d,C}}}$ are strongly correlated with a negative Pearson correlation coefficient (Fig. S2, in dark blue).

IV. ADDITIONAL DETAILS ON THE SISSO APPROACH

The SISSO approach[21] identifies (nonlinear) analytical expressions that describe a given data set. Starting from the candidate descriptive parameters, also called primary features, SISSO builds an immense pool of analytical expressions (e.g., containing billions of expressions) by iteratively applying mathematical operators such as addition, subtraction, exponential, and more to the primary features and to previously generated expressions. Then, by compressed sensing[22, 23] SISSO identifies the few expressions that, combined by weighting coefficients, best correlate with a given target of interest, here the selectivity. Typically, only few key descriptive parameters, out of all initially offered candidates, appear in the models identified by SISSO. The predictive performance and optimal complexity of the SISSO models were assessed by a nested five-fold cross-validation scheme. By determining SISSO model hyperparameters and thus the model complexity via cross validation, we prevent overfitting. Additional details on the SISSO approach are described in references[24–26].

We used the SISSO++[27] implementation of the SISSO approach. We consider the following mathematical operators, where ϕ_i and ϕ_j are two arbitrary features: ϕ_i, $|\phi_i|$, $\exp(\phi_i)$, $\exp(-\phi_i)$ $\ln(\phi_i)$, ϕ_i^{-1}, ϕ_i^2, ϕ_i^3, ϕ_i^6, $\phi_i^{1/2}$, $\phi_i^{1/3}$, $\phi_i + \phi_j$, $\phi_i - \phi_j$, $\phi_i * \phi_j$, $\frac{\phi_i}{\phi_j}$, $|\phi_i - \phi_j|$. The units of the primary features are respected so that terms such as $\ln(\overline{\Delta W^{\mathrm{surf}}_{\mathrm{H}}})$ and $\overline{\Delta W^{\mathrm{surf}}_{\mathrm{H}}} + D_\mu$ are not allowed. Additionally, the coefficients of the SISSO model c_i have units. Thus, the unit of each analytical expression times the unit of the associated coefficient is consistent with the unit of the target being modeled. Finally, we used a nonlinear optimization during the generation of expressions to include scale and bias terms for the mathematical operations logarithm and exponential.[26] Such parametrization turned out to be important to describe the time dependency in the exponential of Eq. 4, for instance. We use a number of residuals equal to 5 and a SIS-selected subspace size of 50 per dimension and apply multi-task SISSO approach[28] in order to model the temperature dependency of the selectivity. In this approach, different weighting coefficients are fitted for the two applied T_{oven} present in the dataset.

A nested five-fold cross-validation scheme was used in order to assess the optimal complexity of the SISSO models and their predictive performance. This procedure is explained in detail elsewhere.[25] In the outer loop of the cross-validation scheme, the dataset containing 539 data points is randomly split into five folds with equal size. Each fold is then used as *test set* once, while the remaining four folds are used to train a model. In the inner loop of the cross-validation scheme, a new 5-fold split is done, with each fold being used as *validation set* once, while the remaining four folds are used to train a model. Within SISSO, the model complexity is controlled by the rung q used to construct the pool of expressions and by the descriptor dimension D. Thus, q and D can be considered as hyperparameters in the SISSO approach. Here we consider descriptors with $D = 1$ up to $D = 5$ and $q = 1, 2$. Fig. S3, S4, and S5 show the validation errors (evaluated on the validation sets) for the three targets we modeled with SISSO, $S_{\mathrm{C_2H_4}}, S_{\mathrm{C_2H_6}}, S_{\mathrm{C_4}}$, respectively. In general, the root-mean-squared cross-validation errors (RMSEs) for $q = 2$ are lower than those for $q = 1$. Additionally, the validation errors initially decrease with increasing D, and then stabilize or increase for higher D. The variability of RMSE according to the split, indicated by the error bars in Fig. S3, S4, and S5, in general increases with increasing D. Base on these results, we take $q = 2$ and $D = 3$ as the optimal complexity for the three targets. The distribution of test errors (evaluated on the test sets) obtained with such model complexity (Fig. S6) indicates a good predictive performance of the obtained models. The mean error values, for instance, indicated by the crosses in Fig. S6, are below 25% of the standard deviation of the target distribution over the entire training dataset for the three considered targets. A detailed analysis of test errors of the SISSO model for ethylene selectivity shows that the highest errors are associated to the Pd-Au and Pd-Cu materials (Fig. S7, left panel). Additionally, higher errors are found in the case of low selectivity values. Thus, the model presents an overall better description for the highly selective scenarios and the Pd-Ag materials. For the analysis of key parameters (Eq. 4, 5, and S9), we trained SISSO models using the entire dataset at the optimal complexity identified. This corresponds to $q = 2, D = 3$ for the three targets.

Different analytical expressions identified by the SISSO approach provide models with similar errors as the model with the lowest error. These expressions might contain different mathematical operators or different descriptive parameters. While the descriptor function might not have physical meaning, the parameters entering the function do have a physical meaning. These parameters correlate with the relevant underlying physical processes. We also note that the set of parameters required to describe a certain target of interest might not be unique, e.g., due to the fact that primary features are correlated (see Fig. S2). The analysis of the analytical expressions associated to

the 50 best models identified by SISSO for $S_{C_2H_4}$ (see Tables II and III) illustrates this phenomenon. All the 50 models contain the exponential term of Eq. 4, which depend on the parameters t_{OS} and $\overline{E^{sub}_{b,H}}$. However, the two other expressions of these 50 models present different mathematical operators or parameters, even though the parameters $\overline{\Delta W^{surf}_{H}}$ and D_μ are present in the first and second terms, respectively, of all 50 models.

The AI analysis is available at `https://github.com/lfoppa/Focused-AI-SGD-SISSO-acetylene-hydrogenation`.

V. ADDITIONAL DETAILS ON THE EXPLOITATION OF THE AI MODELS TO DESIGN BIMETALLIC AND TRIMETALLIC SYSTEMS

To make the predictions shown in Fig. 4B-E, all parameters appearing in Eq. 4 need to be specified. However, the experimental parameters w_{metal} and D_μ are unknown for materials that were not yet synthesized and characterized. In order to make the predictions, we made the assumption that w_{metal} and D_μ for the new, hypothetical materials are equal to the mean values of these parameters in the training set: 0.012 and 5.67 nm, respectively. Thus, the predictions of Fig. 4B-E might deviate from the actual values if the systems exhibit w_{metal} and D_μ values significantly different from the mean values of the training set. Even though we are unable to verify whether such assumption is reasonable for all the hypothetical materials in Fig. 4, the predictions of Fig. 4B-E give an overview of trends and promising compositions.

The SGD and SISSO models obtained here do not take into account the thermodynamic stability nor the synthesizability of the materials. However, Ru-Pd,[29] Rh-Pd,[30] and Pt-Pd[31] systems are not expected to be miscible at any composition according to the phase diagrams. Fe-Pd[32] (e.g., Fe_1Pd_1, Fe_1Pd_3) Co-Pd[33] (e.g., Co_1Pd_1) and Ni-Pd[34] alloys, in contrast, are thermodynamically stable.

VI. SISSO MODEL FOR ETHANE SELECTIVITY

In addition to the model for ethylene and C_4 selectivities, discussed in the main text, we trained models for ethane selectivity. The best models identified for $S_{C_2H_6}$ is

$$\begin{aligned} S^{SISSO}_{C_2H_6} =& c_0^{C_2H_6} \\ &+ c_1^{C_2H_6} \frac{s_{BET}(\overline{\delta^{sub}_{C}})^6}{t_{OS}} \\ &+ c_2^{C_2H_6} \frac{(D_\sigma + D_\mu)\overline{E^{sub}_{d,C}}}{\overline{EN}} \\ &+ c_3^{C_2H_6} exp[1.23 * 10^{-3} \frac{eV}{min}(\frac{t_{OS} + 678min}{\overline{E^{sub}_{b,H}} + 1.23eV})] \end{aligned} \tag{S9}$$

.

[1] Amol P. Amrute, Jacopo De Bellis, Michael Felderhoff, and Ferdi Schüth. Mechanochemical synthesis of catalytic materials. *Chemistry – A European Journal*, 27(23):6819–6847, 2021.

[2] Jacopo De Bellis, Hilke Petersen, Jan Ternieden, Norbert Pfänder, Claudia Weidenthaler, and Ferdi Schüth. Direct dry synthesis of supported bimetallic catalysts: A study on comminution and alloying of metal nanoparticles. *Angewandte Chemie International Edition*, 61(40):e202208016, 2022.

[3] Klara Sophia Kley, Jacopo De Bellis, and Ferdi Schüth. Selective hydrogenation of highly concentrated acetylene streams over mechanochemically synthesized pdag supported catalysts. *Catalysis Science and Technology*, 13:119–131, 2023.

[4] K. K. Kley. *PhD thesis: Catalytic Hydrogenation of Acetylene and Synthesis of Oxymethylene Ether in the Gas Phase.* PhD thesis, Ruhr-Universität Bochum, 2022.

[5] I.-T. Trotuş. *PhD thesis: Catalytic Conversion of Acetylene to Butadiene and Butenes.* PhD thesis, Ruhr-Universität Bochum, 2016.

[6] Ioan-Teodor Trotuş, Tobias Zimmermann, Nicolas Duyckaerts, Jan Geboers, and Ferdi Schüth. Butadiene from acetylene–ethylene cross-metathesis. *Chemical Communications*, 51:7124–7127, 2015.

[7] Özgül Agbaba, Ioan-Teodor Trotuş, Wolfgang Schmidt, and Ferdi Schüth. Light olefins from acetylene under pressurized conditions. *Industrial & Engineering Chemistry Research*, 62(4):1819–1825, Feb 2023.

[8] Jonathan M. Mauß and Ferdi Schüth. On the role of anions in solid catalysts with ionic liquid layer (scill) for the selective hydrogenation of highly concentrated acetylene streams. *ChemSusChem*, 18(2):e202401593, 2025.

[9] Jake O. Williams, Ella Kitching, Rhea-Shree Patel, Jonathan M. Mauß, Klara S. Kley, Rohini Khobragade, Jacopo de Bellis, David J. Morgan, Thomas Slater, Ferdi Schüth, Stuart H. Taylor, Nicholas F. Dummer, Michael Bender, and Graham J. Hutchings. The influence of reaction conditions on selective acetylene hydrogenation over sol immobilization prepared agpd/al2o3 catalysts. *ChemCatChem*, 17(8):e202401794, 2025.

[10] Yufei He, Jiaxuan Fan, Junting Feng, Chiying Luo, Pengfei Yang, and Dianqing Li. Pd nanoparticles on hydrotalcite as an efficient catalyst for partial hydrogenation of acetylene: Effect of support acidic and basic properties. *Journal of Catalysis*, 331:118–127, 2015.

[11] Samane Komeili, Maryam Takht Ravanchi, and Abbas Taeb. The influence of alumina phases on the perfor-

Table S I. Candidate descriptive parameters (primary features) applied in the AI analysis. These experimental (Exp.) and theoretical parameters characterize the materials, reaction conditions, and correlate with possible underlying processes. [a]The materials Pd_1Ag_1, Pd_1Ag_5, and Pd_1Ag_9 are reported in [3], while the synthesis and characterisation of the gold- and copper-based materials are reported in [4]. [b]Properties of the face-center-cubic (fcc) crystal structure for nickel, copper, rhodium, palladium, silver, iridium, platinum and gold, to the hexagonal-closed-packed (hcp) crystal structure for cobalt and ruthenium, and for the body-center-cubic (bcc) crystal structure for iron. [c]Properties of the (111), (0001), and (110) surfaces for the metals with fcc, hcp, and bcc crystal structures, respectively. The properties of most stable surface adsorption sites are considered.

Type	Name	Symbol	Unit	Method	Ref.
-	Time On Stream	t_{OS}	min	Exp.	[3, 4][a]
Reaction	Temperature of Oven	T_{oven}	° C	Exp.	[3, 4][a]
Entire Material	Total Metal Loading (Weight Fraction)	w_{metal}	-	Exp.	[3, 4][a]
Entire Material	Mean Particle Diameter	D_μ	Å	Exp.	[3, 4][a]
Entire Material	Standard Deviation of Particle-Diameter Distribution	D_σ	Å	Exp.	[3, 4][a]
Entire Material	Specific Surface Area	s_{BET}	m^2/g	Exp.	[3, 4][a]
Free-atom	Composition-Averaged Ionization Potential	$\overline{IP}$	eV	Exp.	[35]
Free-atom	Composition-Averaged Electron Affinity	$\overline{EA}$	eV	Exp.	[36]
Free-atom	Composition-Averaged Pauling Electronegativity	$\overline{EN}$	-	Exp.	[36]
Bulk[b]	Composition-Averaged Closest Interatomic Distance	$\overline{d_{\mathrm{closest}}}$	Å	Exp.	[37]
Bulk[b]	Composition-Averaged Cohesive Energy	$\overline{E_{\mathrm{coh}}}$	eV/atom	Exp.	[37]
Bulk[b]	Composition-Averaged Bulk Modulus	$\overline{B_0}$	GPa	Exp.	[37]
Clean Surface[c]	Composition-Averaged Energy of the *d*-Band Center	$\overline{\epsilon_d}$	eV	DFT-PW91	[38]
Surface with C[c]	Composition-Averaged Critical Surface Carbon Chemical Potential	$\overline{\mu_{\mathrm{C}}^{\mathrm{surf}}}$	eV	DFT-PW91	[39]
Surface with C[c]	Composition-Averaged Critical Subsurface Carbon Chemical Potential	$\overline{\mu_{\mathrm{C}}^{\mathrm{sub}}}$	eV	DFT-PW91	[39]
Surface with C[c]	Composition-Averaged Distance Expansion due to Subsurface Carbon	$\overline{\delta_{\mathrm{C}}^{\mathrm{sub}}}$	Å	DFT-PW91	[39]
Surface with C[c]	Composition-Averaged Surface Deformation Energy due to Subsurface Carbon	$\overline{E_{\mathrm{d,C}}^{\mathrm{sub}}}$	eV	DFT-PW91	[39]
Surface with C[c]	Composition-Averaged Subsurface Carbon Binding Energy	$\overline{E_{\mathrm{b,C}}^{\mathrm{sub}}}$	eV	DFT-PW91	[39]
Surface with H[c]	Composition-Averaged Surface Binding Energy	$\overline{E_{\mathrm{b,H}}^{\mathrm{surf}}}$	eV	DFT-PW91	[38]
Surface with H[c]	Composition-Averaged Subsurface Binding Energy	$\overline{E_{\mathrm{b,H}}^{\mathrm{sub}}}$	eV	DFT-PW91	[38]
Surface with H[c]	Composition-Averaged Work-Function Change due to Hydrogen Adsorption	$\overline{\Delta W_{\mathrm{H}}^{\mathrm{surf}}}$	eV	DFT-PW91	[38]

mance of the pd–ag/al2o3 catalyst in tail-end selective hydrogenation of acetylene. *Applied Catalysis A: General*, 502:287–296, 2015.

[12] Alan J McCue and James A Anderson. Recent advances in selective acetylene hydrogenation using palladium containing catalysts. *Frontiers of Chemical Science and Engineering*, 9:142–153, 2015.

[13] Stefan Wrobel. An algorithm for multi-relational discovery of subgroups. In *European Conference on Principles of Data Mining and Knowledge Discovery*, 1997.

[14] Martin Atzmueller. Subgroup discovery. *WIREs Data Mining and Knowledge Discovery*, 5(1):35–49, 2015.

[15] Lucas Foppa, Christopher Sutton, Luca M. Ghiringhelli, Sandip De, Patricia Löser, Stephan A. Schunk, Ansgar Schäfer, and Matthias Scheffler. Learning design rules for selective oxidation catalysts from high-throughput experimentation and artificial intelligence. *ACS Catalysis*, 12:2223, 2022.

[16] Lucas Foppa and Luca M. Ghiringhelli. Identifying outstanding transition-metal-alloy heterogeneous catalysts for the oxygen reduction and evolution reactions via subgroup discovery. *Topics in Catalysis*, 65:196, Jan 2022.

[17] Lucas Foppa and Matthias Scheffler. Coherent collections of rules describing exceptional materials identified with a multi-objective optimization of subgroups. *Digital Discovery*, page DOI: 10.1039/D5DD00174A, 2025.

[18] Bryan R Goldsmith, Mario Boley, Jilles Vreeken, Matthias Scheffler, and Luca M Ghiringhelli. Uncovering structure-property relationships of materials by subgroup discovery. *New Journal of Physics*, 19(1):013031, jan 2017.

[19] Mario Boley, Claudio Lucchese, Daniel Paurat, and Thomas Gärtner. Direct local pattern sampling by efficient two-step random procedures. In *Proceedings of the 17th ACM SIGKDD International Conference on Knowledge Discovery and Data Mining*, KDD '11, page 582–590, New York, NY, USA, 2011. Association for Computing Machinery.

[20] Mario Boley, Sandy Moens, and Thomas Gärtner. Linear space direct pattern sampling using coupling from the past. In *Proceedings of the 18th ACM SIGKDD International Conference on Knowledge Discovery and Data Mining*, KDD '12, page 69–77, New York, NY, USA, 2012. Association for Computing Machinery.

Table S II. Expressions associated to the 50 best SISSO models for ethylene selectivity ($S_{C_2H_4}^{SISSO}$) according to the training root mean squared error (RMSE). d_1, d_2, and d_3 correspond to the three expressions of the models, or three descriptor components.

Train RMSE	d_1	d_2	d_3
0.113	$((t_{OS}/\overline{\Delta W_{H}^{surf}})/(\lvert\overline{\epsilon_d} - \overline{E_{b,H}^{surf}}\rvert))$	$((D_\mu * w_{metal}) * \overline{E_{d,C}^{sub}})$	$exp(8.42 * 10^{-4}(\frac{t_{OS}+365.6}{\overline{E_{b,H}^{sub}}+1.38}))$
0.114	$((t_{OS}/\overline{\Delta W_{H}^{surf}})/(\lvert\overline{\epsilon_d} - \overline{E_{b,H}^{surf}}\rvert))$	$((\overline{d_{closest}}/\overline{E_{d,C}^{sub}})/(D_\mu * w_{metal}))$	$exp(8.42 * 10^{-4}(\frac{t_{OS}+365.6}{\overline{E_{b,H}^{sub}}+1.38}))$
0.115	$((t_{OS}/\overline{\Delta W_{H}^{surf}})/(\overline{\epsilon_d} - \overline{E_{b,H}^{surf}}))$	$((D_\mu * w_{metal}) * \overline{E_{d,C}^{sub}})$	$exp(8.42 * 10^{-4}(\frac{t_{OS}+365.6}{\overline{E_{b,H}^{sub}}+1.38}))$
0.116	$((t_{OS}/\overline{\Delta W_{H}^{surf}})/(\lvert\overline{\epsilon_d} - \overline{E_{b,H}^{surf}}\rvert))$	$((D_\sigma + D_\mu) * (\overline{E_{d,C}^{sub}} * w_{metal}))$	$exp(8.42 * 10^{-4}(\frac{t_{OS}+365.6}{\overline{E_{b,H}^{sub}}+1.38}))$
0.116	$((t_{OS}/\overline{\Delta W_{H}^{surf}})/(\overline{\epsilon_d} - \overline{E_{b,H}^{surf}}))$	$((\overline{d_{closest}}/\overline{E_{d,C}^{sub}})/(D_\mu * w_{metal}))$	$exp(8.42 * 10^{-4}(\frac{t_{OS}+365.6}{\overline{E_{b,H}^{sub}}+1.38}))$
0.116	$((t_{OS}/\overline{\Delta W_{H}^{surf}})/(\overline{\epsilon_d} - \overline{E_{b,H}^{surf}}))$	$((D_\mu/\overline{E_{coh}}) * (\overline{E_{d,C}^{sub}} * w_{metal}))$	$exp(8.42 * 10^{-4}(\frac{t_{OS}+365.6}{\overline{E_{b,H}^{sub}}+1.38}))$
0.117	$((t_{OS}/\overline{\Delta W_{H}^{surf}})/(\lvert\overline{\epsilon_d} - \overline{E_{b,H}^{surf}}\rvert))$	$((D_\mu/\overline{d_{closest}}) * (\overline{E_{d,C}^{sub}} * w_{metal}))$	$exp(8.42 * 10^{-4}(\frac{t_{OS}+365.6}{\overline{E_{b,H}^{sub}}+1.38}))$
0.117	$((t_{OS}/\overline{\Delta W_{H}^{surf}})/(\overline{\epsilon_d} - \overline{E_{b,H}^{surf}}))$	$((D_\mu/\overline{d_{closest}}) * (\overline{E_{d,C}^{sub}} * w_{metal}))$	$exp(8.42 * 10^{-4}(\frac{t_{OS}+365.6}{\overline{E_{b,H}^{sub}}+1.38}))$
0.117	$((t_{OS}/\overline{\Delta W_{H}^{surf}})/(\lvert\overline{\epsilon_d} - \overline{E_{b,H}^{surf}}\rvert))$	$((\overline{E_{coh}}/w_{metal})/(\overline{E_{d,C}^{sub}} * D_\mu))$	$exp(8.42 * 10^{-4}(\frac{t_{OS}+365.6}{\overline{E_{b,H}^{sub}}+1.38}))$
0.117	$((t_{OS}/\overline{\Delta W_{H}^{surf}})/(\overline{\epsilon_d} - \overline{E_{b,H}^{surf}}))$	$((D_\mu * w_{metal}) * (\overline{E_{d,C}^{sub}}/\overline{IP}))$	$exp(8.42 * 10^{-4}(\frac{t_{OS}+365.6}{\overline{E_{b,H}^{sub}}+1.38}))$
0.117	$((t_{OS}/\overline{\Delta W_{H}^{surf}})/(\lvert\overline{\epsilon_d} - \overline{E_{b,H}^{surf}}\rvert))$	$((D_\mu/\overline{E_{coh}}) * (\overline{E_{d,C}^{sub}} * w_{metal}))$	$exp(8.42 * 10^{-4}(\frac{t_{OS}+365.6}{\overline{E_{b,H}^{sub}}+1.38}))$
0.118	$((\overline{\Delta W_{H}^{surf}} * t_{OS})/(\lvert\overline{\epsilon_d} - \overline{E_{b,H}^{surf}}\rvert))$	$((\overline{d_{closest}}/\overline{E_{d,C}^{sub}})/(D_\mu * w_{metal}))$	$exp(8.42 * 10^{-4}(\frac{t_{OS}+365.6}{\overline{E_{b,H}^{sub}}+1.38}))$
0.118	$((\overline{\Delta W_{H}^{surf}} * t_{OS})/(\lvert\overline{\epsilon_d} - \overline{E_{b,H}^{surf}}\rvert))$	$((D_\mu * w_{metal}) * \overline{E_{d,C}^{sub}})$	$exp(8.42 * 10^{-4}(\frac{t_{OS}+365.6}{\overline{E_{b,H}^{sub}}+1.38}))$
0.118	$((t_{OS}/\overline{\Delta W_{H}^{surf}})/(\overline{\epsilon_d} - \overline{E_{b,H}^{surf}}))$	$((\overline{E_{coh}}/w_{metal})/(\overline{E_{d,C}^{sub}} * D_\mu))$	$exp(8.42 * 10^{-4}(\frac{t_{OS}+365.6}{\overline{E_{b,H}^{sub}}+1.38}))$
0.118	$((t_{OS}/\overline{\Delta W_{H}^{surf}})/(\lvert\overline{\epsilon_d} - \overline{E_{b,H}^{surf}}\rvert))$	$((D_\mu * w_{metal}) * (\overline{E_{d,C}^{sub}}/\overline{IP}))$	$exp(8.42 * 10^{-4}(\frac{t_{OS}+365.6}{\overline{E_{b,H}^{sub}}+1.38}))$
0.120	$((t_{OS}/\overline{\Delta W_{H}^{surf}})/(\lvert\overline{\epsilon_d} - \overline{E_{b,H}^{surf}}\rvert))$	$((\overline{\Delta W_{H}^{surf}} * D_\sigma) + (\overline{E_{d,C}^{sub}} * D_\mu))$	$exp(8.42 * 10^{-4}(\frac{t_{OS}+365.6}{\overline{E_{b,H}^{sub}}+1.38}))$
0.120	$((t_{OS}/\overline{\Delta W_{H}^{surf}})/(\overline{\epsilon_d} - \overline{E_{b,H}^{surf}}))$	$((\overline{IP}/D_\mu)/(\overline{E_{d,C}^{sub}} * w_{metal}))$	$exp(8.42 * 10^{-4}(\frac{t_{OS}+365.6}{\overline{E_{b,H}^{sub}}+1.38}))$
0.120	$((t_{OS}/\overline{\Delta W_{H}^{surf}})/(\lvert\overline{\epsilon_d} - \overline{E_{b,H}^{surf}}\rvert))$	$((\overline{IP}/D_\mu)/(\overline{E_{d,C}^{sub}} * w_{metal}))$	$exp(8.42 * 10^{-4}(\frac{t_{OS}+365.6}{\overline{E_{b,H}^{sub}}+1.38}))$
0.120	$((t_{OS}/\overline{\Delta W_{H}^{surf}})/(\lvert\overline{\epsilon_d} - \overline{E_{b,H}^{surf}}\rvert))$	$((\overline{E_{d,C}^{sub}} * D_\mu)/(\overline{IP}^2))$	$exp(8.42 * 10^{-4}(\frac{t_{OS}+365.6}{\overline{E_{b,H}^{sub}}+1.38}))$
0.120	$((t_{OS}/\overline{\Delta W_{H}^{surf}})/(\lvert\overline{\epsilon_d} - \overline{E_{b,H}^{surf}}\rvert))$	$((D_\mu/\overline{B_0}) * (\overline{E_{d,C}^{sub}} * \overline{\mu_{C}^{sub}}))$	$exp(8.42 * 10^{-4}(\frac{t_{OS}+365.6}{\overline{E_{b,H}^{sub}}+1.38}))$
0.120	$((t_{OS}/\overline{\Delta W_{H}^{surf}})/(\lvert\overline{\epsilon_d} - \overline{E_{b,H}^{surf}}\rvert))$	$((\overline{E_{d,C}^{sub}} * D_\mu)/(\overline{E_{coh}} * \overline{EN}))$	$exp(8.42 * 10^{-4}(\frac{t_{OS}+365.6}{\overline{E_{b,H}^{sub}}+1.38}))$
0.120	$((t_{OS}/\overline{\Delta W_{H}^{surf}})/(\lvert\overline{\epsilon_d} - \overline{E_{b,H}^{surf}}\rvert))$	$((D_\mu/\overline{\mu_{C}^{surf}})/(\overline{d_{closest}}^6))$	$exp(8.42 * 10^{-4}(\frac{t_{OS}+365.6}{\overline{E_{b,H}^{sub}}+1.38}))$
0.120	$((t_{OS}/\overline{\Delta W_{H}^{surf}})/(\overline{\epsilon_d} - \overline{E_{b,H}^{surf}}))$	$((D_\sigma + D_\mu) * (\overline{E_{d,C}^{sub}} * w_{metal}))$	$exp(8.42 * 10^{-4}(\frac{t_{OS}+365.6}{\overline{E_{b,H}^{sub}}+1.38}))$
0.120	$((\overline{\Delta W_{H}^{surf}} * t_{OS})/(\overline{\epsilon_d} - \overline{E_{b,H}^{surf}}))$	$((\overline{d_{closest}}/\overline{E_{d,C}^{sub}})/(D_\mu * w_{metal}))$	$exp(8.42 * 10^{-4}(\frac{t_{OS}+365.6}{\overline{E_{b,H}^{sub}}+1.38}))$
0.120	$((\overline{\Delta W_{H}^{surf}} * t_{OS})/(\overline{\epsilon_d} - \overline{E_{b,H}^{surf}}))$	$((D_\mu * w_{metal}) * \overline{E_{d,C}^{sub}})$	$exp(8.42 * 10^{-4}(\frac{t_{OS}+365.6}{\overline{E_{b,H}^{sub}}+1.38}))$
0.120	$((\overline{\Delta W_{H}^{surf}} * t_{OS})/(\lvert\overline{\epsilon_d} - \overline{E_{b,H}^{surf}}\rvert))$	$((D_\mu/\overline{d_{closest}}) * (\overline{E_{d,C}^{sub}} * w_{metal}))$	$exp(8.42 * 10^{-4}(\frac{t_{OS}+365.6}{\overline{E_{b,H}^{sub}}+1.38}))$
0.121	$((t_{OS}/\overline{\Delta W_{H}^{surf}})/(\lvert\overline{\epsilon_d} - \overline{E_{b,H}^{surf}}\rvert))$	$((D_\mu/\overline{E_{b,H}^{sub}})/(\overline{d_{closest}}^6))$	$exp(8.42 * 10^{-4}(\frac{t_{OS}+365.6}{\overline{E_{b,H}^{sub}}+1.38}))$
0.121	$((\overline{\Delta W_{H}^{surf}} * t_{OS})/(\lvert\overline{\epsilon_d} - \overline{E_{b,H}^{surf}}\rvert))$	$((D_\sigma + D_\mu) * (\overline{E_{d,C}^{sub}} * w_{metal}))$	$exp(8.42 * 10^{-4}(\frac{t_{OS}+365.6}{\overline{E_{b,H}^{sub}}+1.38}))$
0.121	$((t_{OS}/\overline{\Delta W_{H}^{surf}})/(\lvert\overline{\epsilon_d} - \overline{E_{b,H}^{surf}}\rvert))$	$((D_\mu/\overline{B_0}) * (\overline{E_{d,C}^{sub}} * \overline{E_{b,C}^{sub}}))$	$exp(8.42 * 10^{-4}(\frac{t_{OS}+365.6}{\overline{E_{b,H}^{sub}}+1.38}))$
0.121	$((\overline{\Delta W_{H}^{surf}} * t_{OS})/(\lvert\overline{\epsilon_d} - \overline{E_{b,H}^{surf}}\rvert))$	$((\overline{E_{coh}}/w_{metal})/(\overline{E_{d,C}^{sub}} * D_\mu))$	$exp(8.42 * 10^{-4}(\frac{t_{OS}+365.6}{\overline{E_{b,H}^{sub}}+1.38}))$
0.121	$((\overline{\Delta W_{H}^{surf}} * t_{OS})/(\overline{\epsilon_d} - \overline{E_{b,H}^{surf}}))$	$((D_\mu/\overline{d_{closest}}) * (\overline{E_{d,C}^{sub}} * w_{metal}))$	$exp(8.42 * 10^{-4}(\frac{t_{OS}+365.6}{\overline{E_{b,H}^{sub}}+1.38}))$
0.121	$((t_{OS}/\overline{\Delta W_{H}^{surf}})/(\lvert\overline{\epsilon_d} - \overline{E_{b,H}^{surf}}\rvert))$	$((\overline{E_{coh}} * \overline{EN})/(\overline{E_{d,C}^{sub}} * D_\mu))$	$exp(8.42 * 10^{-4}(\frac{t_{OS}+365.6}{\overline{E_{b,H}^{sub}}+1.38}))$
0.122	$((t_{OS}/\overline{\Delta W_{H}^{surf}})/(\overline{\epsilon_d} - \overline{E_{b,H}^{surf}}))$	$((w_{metal}/\overline{EN}) * (\overline{E_{d,C}^{sub}} * D_\mu))$	$exp(8.42 * 10^{-4}(\frac{t_{OS}+365.6}{\overline{E_{b,H}^{sub}}+1.38}))$
0.122	$((t_{OS}/\overline{\Delta W_{H}^{surf}})/(\overline{\epsilon_d} - \overline{E_{b,H}^{surf}}))$	$((\overline{\Delta W_{H}^{surf}} * D_\sigma) + (\overline{E_{d,C}^{sub}} * D_\mu))$	$exp(8.42 * 10^{-4}(\frac{t_{OS}+365.6}{\overline{E_{b,H}^{sub}}+1.38}))$
0.122	$((\overline{\Delta W_{H}^{surf}} * t_{OS})/(\lvert\overline{\epsilon_d} - \overline{E_{b,H}^{surf}}\rvert))$	$((D_\mu/\overline{E_{coh}}) * (\overline{E_{d,C}^{sub}} * w_{metal}))$	$exp(8.42 * 10^{-4}(\frac{t_{OS}+365.6}{\overline{E_{b,H}^{sub}}+1.38}))$

Table S III. Cont. Expressions associated to the 50 best SISSO models for ethylene selectivity ($S^{SISSO}_{C_2H_4}$) according to the training root mean squared error (RMSE). d_1, d_2, and d_3 correspond to the three expressions of the models, or three descriptor components.

Train RMSE	d_1	d_2	d_3
0.122	$((\overline{\Delta W^{surf}_{H}} * t_{OS})/(\overline{\epsilon_d} - \overline{E^{surf}_{b,H}}))$	$((D_\mu/\overline{E_{coh}}) * (\overline{E^{sub}_{d,C}} * w_{metal}))$	$exp(8.42 * 10^{-4}(\frac{\overline{t_{OS}}+365.6}{\overline{E^{sub}_{b,H}}+1.38}))$
0.122	$((\overline{\Delta W^{surf}_{H}} * t_{OS})/(\overline{\epsilon_d} - \overline{E^{surf}_{b,H}}))$	$((D_\mu * w_{metal}) * (\overline{E^{sub}_{d,C}}/\overline{IP}))$	$exp(8.42 * 10^{-4}(\frac{\overline{t_{OS}}+365.6}{\overline{E^{sub}_{b,H}}+1.38}))$
0.122	$((t_{OS}/\overline{\Delta W^{surf}_{H}})/(\lvert\overline{\epsilon_d} - \overline{E^{surf}_{b,H}}\rvert))$	$((\overline{d_{closest}}^6) * (\overline{\mu^{surf}_{C}}/D_\mu))$	$exp(8.42 * 10^{-4}(\frac{\overline{t_{OS}}+365.6}{\overline{E^{sub}_{b,H}}+1.38}))$
0.122	$((t_{OS}/\overline{\Delta W^{surf}_{H}})/(\overline{\epsilon_d} - \overline{E^{surf}_{b,H}}))$	$((D_\mu/\overline{\mu^{surf}_{C}})/(\overline{d_{closest}}^6))$	$exp(8.42 * 10^{-4}(\frac{\overline{t_{OS}}+365.6}{\overline{E^{sub}_{b,H}}+1.38}))$
0.122	$((\overline{\Delta W^{surf}_{H}} * t_{OS})/(\lvert\overline{\epsilon_d} - \overline{E^{surf}_{b,H}}\rvert))$	$((D_\mu * w_{metal}) * (\overline{E^{sub}_{d,C}}/\overline{IP}))$	$exp(8.42 * 10^{-4}(\frac{\overline{t_{OS}}+365.6}{\overline{E^{sub}_{b,H}}+1.38}))$
0.122	$((\overline{E^{sub}_{d,C}} * t_{OS})/(\overline{\Delta W^{surf}_{H}}^3))$	$((D_\sigma + D_\mu) * (\overline{E^{sub}_{d,C}} * w_{metal}))$	$exp(8.42 * 10^{-4}(\frac{\overline{t_{OS}}+365.6}{\overline{E^{sub}_{b,H}}+1.38}))$
0.123	$((\overline{\delta^{sub}_{C}} * t_{OS})/(\overline{\Delta W^{surf}_{H}}^3))$	$((D_\sigma + D_\mu) * (\overline{E^{sub}_{d,C}} * w_{metal}))$	$exp(8.42 * 10^{-4}(\frac{\overline{t_{OS}}+365.6}{\overline{E^{sub}_{b,H}}+1.38}))$
0.123	$((t_{OS}/D_\mu)/(\overline{\Delta W^{surf}_{H}}^3))$	$((D_\sigma + D_\mu) * (\overline{E^{sub}_{d,C}} * w_{metal}))$	$exp(8.42 * 10^{-4}(\frac{\overline{t_{OS}}+365.6}{\overline{E^{sub}_{b,H}}+1.38}))$
0.123	$((t_{OS}/\overline{\Delta W^{surf}_{H}})/(\lvert\overline{\epsilon_d} - \overline{E^{surf}_{b,H}}\rvert))$	$((\overline{E^{sub}_{d,C}} * D_\mu)/(\overline{EN} * \overline{IP}))$	$exp(8.42 * 10^{-4}(\frac{\overline{t_{OS}}+365.6}{\overline{E^{sub}_{b,H}}+1.38}))$
0.123	$((t_{OS}/\overline{\Delta W^{surf}_{H}})/(\overline{\epsilon_d} - \overline{E^{surf}_{b,H}}))$	$((D_\mu/\overline{B_0}) * (\overline{E^{sub}_{d,C}} * \overline{\mu^{sub}_{C}}))$	$exp(8.42 * 10^{-4}(\frac{\overline{t_{OS}}+365.6}{\overline{E^{sub}_{b,H}}+1.38}))$
0.123	$((t_{OS}/\overline{\Delta W^{surf}_{H}})/(\overline{\epsilon_d} - \overline{E^{surf}_{b,H}}))$	$((\overline{E^{sub}_{d,C}} * D_\mu)/(\overline{E_{coh}} * \overline{EN}))$	$exp(8.42 * 10^{-4}(\frac{\overline{t_{OS}}+365.6}{\overline{E^{sub}_{b,H}}+1.38}))$
0.123	$((t_{OS}/\overline{\Delta W^{surf}_{H}}) * (\overline{E^{sub}_{d,C}}^6))$	$((\overline{d_{closest}}/\overline{E^{sub}_{d,C}})/(D_\mu * w_{metal}))$	$exp(8.42 * 10^{-4}(\frac{\overline{t_{OS}}+365.6}{\overline{E^{sub}_{b,H}}+1.38}))$
0.123	$((t_{OS}/\overline{\Delta W^{surf}_{H}}) * (\overline{\epsilon_d} - \overline{\mu^{surf}_{C}}))$	$((D_\sigma + D_\mu) * (\overline{E^{sub}_{d,C}} * w_{metal}))$	$exp(8.42 * 10^{-4}(\frac{\overline{t_{OS}}+365.6}{\overline{E^{sub}_{b,H}}+1.38}))$
0.123	$((\overline{\Delta W^{surf}_{H}} * t_{OS})/(\overline{\epsilon_d} - \overline{E^{surf}_{b,H}}))$	$((\overline{E_{coh}}/w_{metal})/(\overline{E^{sub}_{d,C}} * D_\mu))$	$exp(8.42 * 10^{-4}(\frac{\overline{t_{OS}}+365.6}{\overline{E^{sub}_{b,H}}+1.38}))$

[21] Runhai Ouyang, Stefano Curtarolo, Emre Ahmetcik, Matthias Scheffler, and Luca M. Ghiringhelli. SISSO: A compressed-sensing method for identifying the best low-dimensional descriptor in an immensity of offered candidates. *Physical Review Materials*, 2(8):083802, 2018.

[22] E. J. Candes and M. B. Wakin. An introduction to compressive sampling. *IEEE Signal Processing Magazine*, 25(2):21–30, 2008.

[23] Lance J. Nelson, Gus L. W. Hart, Fei Zhou, and Vidvuds Ozoliņš. Compressive sensing as a paradigm for building physics models. *Physical Review B*, 87(3):035125, 2013.

[24] Lucas Foppa, Luca M. Ghiringhelli, Frank Girgsdies, Maike Hashagen, Pierre Kube, Michael Hävecker, Spencer J. Carey, Andrey Tarasov, Peter Kraus, Frank Rosowski, Robert Schlögl, Annette Trunschke, and Matthias Scheffler. Materials genes of heterogeneous catalysis from clean experiments and artificial intelligence. *MRS Bulletin*, 46:1016–1026, Nov 2021.

[25] Lucas Foppa, Thomas A. R. Purcell, Sergey V. Levchenko, Matthias Scheffler, and Luca M. Ghiringhelli. Hierarchical symbolic regression for identifying key physical parameters correlated with bulk properties of perovskites. *Physical Review Letters*, 129:055301, Jul 2022.

[26] Thomas A. R. Purcell, Matthias Scheffler, and Luca M. Ghiringhelli. Recent advances in the SISSO method and their implementation in the SISSO++ code. *The Journal of Chemical Physics*, 159(11):114110, 09 2023.

[27] Thomas A. R. Purcell, Matthias Scheffler, Christian Carbogno, and Luca M Ghiringhelli. Sisso++: A c++ implementation of the sure-independence screening and sparsifying operator approach. *Journal of Open Source Software*, 7(71):3960, 2022.

[28] Runhai Ouyang, Emre Ahmetcik, Christian Carbogno, Matthias Scheffler, and Luca M Ghiringhelli. Simultaneous learning of several materials properties from incomplete databases with multi-task sisso. *Journal of Physics: Materials*, 2(2):024002, mar 2019.

[29] SN Tripathi, SR Bharadwaj, and SR Dharwadkar. The pd-ru system (palladium-ruthenium). *Journal of phase equilibria*, 14:638–642, 1993.

[30] SN Tripathi and SR Bharadwqj. The pd-rh (palladium-rhodium) system. *Journal of phase equilibria*, 15:208–212, 1994.

[31] H Okamoto. Pd-pt (palladium-platinum). *Journal of phase equilibria*, 12(5):617–618, 1991.

[32] Ortrud Kubaschewski von Goldbeck and Ortrud Kubaschewski von Goldbeck. Fe—pd iron—palladium. *IRON—Binary Phase Diagrams*, pages 88–91, 1982.

[33] K Ishida and T Nishizawa. The co-pd (cobalt-palladium) system. *Journal of phase equilibria*, 12:83–87, 1991.

[34] A Nash and P Nash. The ni- pd (nickel-palladium) system. *Bulletin of Alloy Phase Diagrams*, 5(5):446–450, 1984.

[35] S. Lias. Ionization energy evaluation. In *Linstrom, P.J. and Mallard, W.G., Eds., NIST Chemistry WebBook, NIST Standard Reference Database Number 69*, Gaithersburg, USA, 2005. National Institute of Standards and Technology.

[36] WebElements, howpublished = `www.webelements.com`, note = Accessed: 2024-10-10.

[37] Patanachai Janthon, Sijie (Andy) Luo, Sergey M. Kozlov, Francesc Viñes, Jumras Limtrakul, Donald G. Truhlar, and Francesc Illas. Bulk properties of transition met-

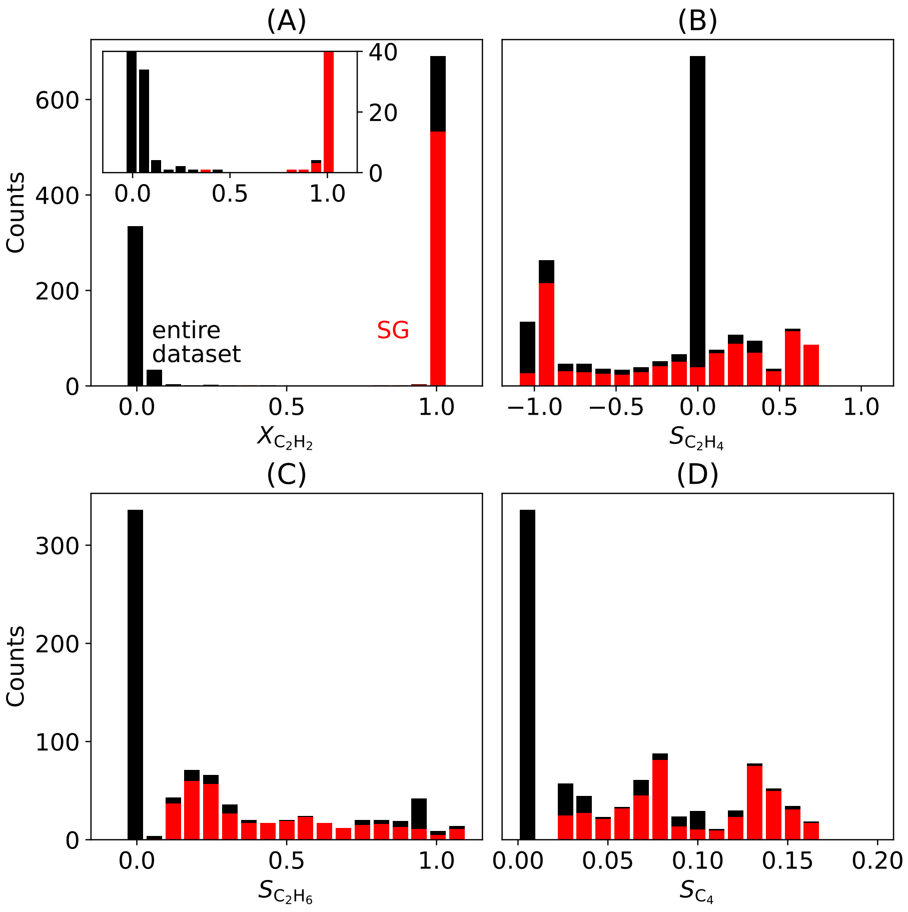

FIG. S1. (A): Distribution of acetylene-conversion values ($X_{C_2H_2}$, Eq. 1 in the main text) in the entire dataset (in black) and in the SG identified with high $X_{C_2H_2}$ (in red).(B): Distribution of ethylene-selectivity values ($S_{C_2H_4}$, Eq. 2 in the main text) in the entire dataset and in the SG identified with high $X_{C_2H_2}$. (C): Distribution of ethane-selectivity values ($S_{C_2H_6}$, Eq. S1) in the entire dataset and in the SG identified with high $X_{C_2H_2}$. (D): Distribution of C$_4$-selectivity values (S_{C_4}, Eq. S2) in the entire dataset and in the SG identified with high $X_{C_2H_2}$.

als: A challenge for the design of universal density functionals. *Journal of Chemical Theory and Computation*, 10(9):3832–3839, 2014. PMID: 26588528.

[38] Jeff Greeley and Manos Mavrikakis. Surface and subsurface hydrogen: Adsorption properties on transition metals and near-surface alloys. *The Journal of Physical Chemistry B*, 109:3460–3471, 2005.

[39] Philippe Sautet and Fabrizio Cinquini. Surface of metallic catalysts under a pressure of hydrocarbon molecules: Metal or carbide? *ChemCatChem*, 2(6):636–639, 2010.

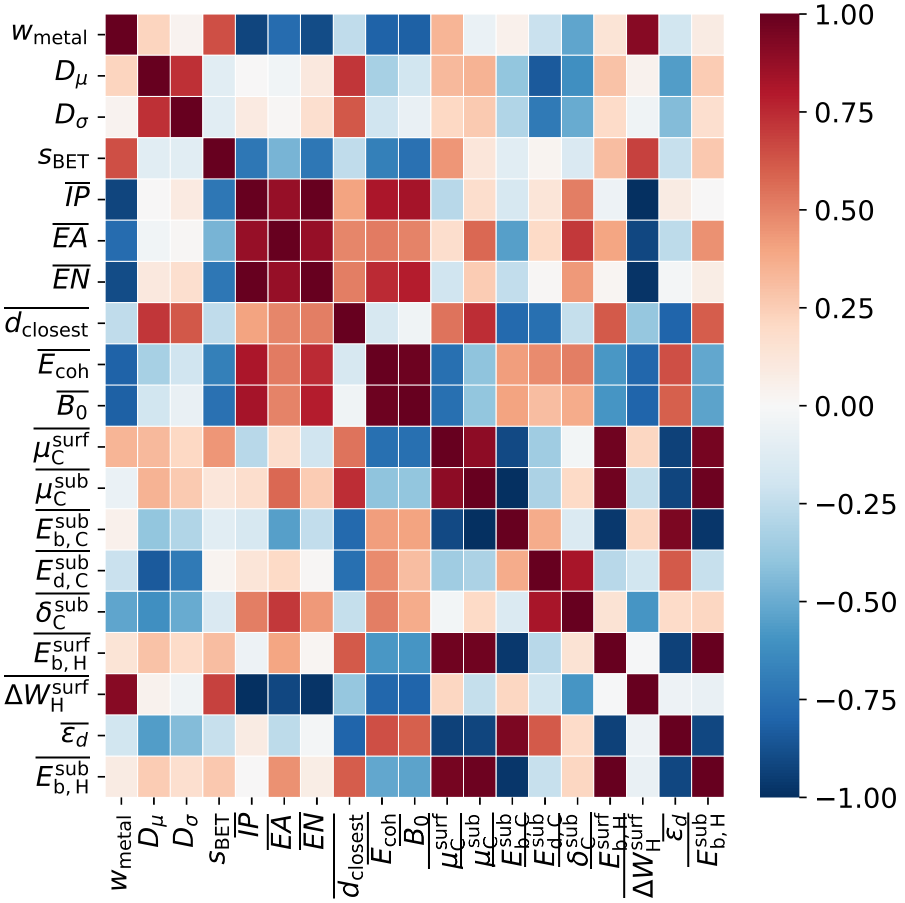

FIG. S2. Pearson correlation analysis between the candidate descriptive parameters (or primary features). The correlation scores were evaluated using the dataset of 539 data points (training dataset for the SISSO models).

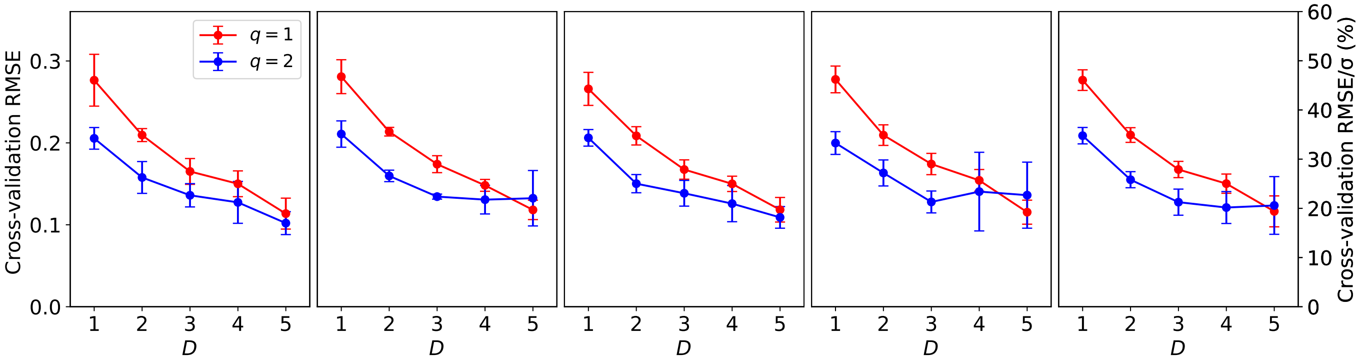

FIG. S3. Cross-validation analysis to assess the optimal complexity of the SISSO models for the ethylene selectivity ($S_{C_2H_4}$). Each panel correspond to one different outer-loop split. The validation root-mean-squared errors (RMSE) and error bars correspond to the average and standard deviation of five RMSE values obtained in the inner-loop splits of the nested cross-validation scheme. The y-axis on the right shows the RMSE as a percentage of the standard deviation of the target $S_{C_2H_4}$ over the entire dataset.

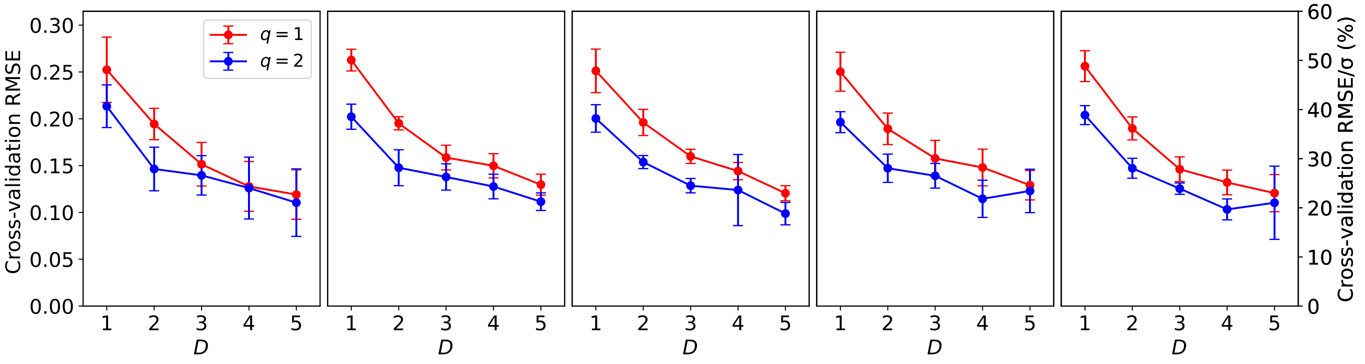

FIG. S4. Cross-validation analysis to assess the optimal complexity of the SISSO models for the ethane selectivity ($S_{C_2H_6}$). Each panel correspond to one different outer-loop split. The validation root-mean-squared errors (RMSE) and error bars correspond to the average and standard deviation of five RMSE values obtained in the inner-loop splits of the nested cross-validation scheme. The y-axis on the right shows the RMSE as a percentage of the standard deviation of the target $S_{C_2H_6}$ over the entire dataset.

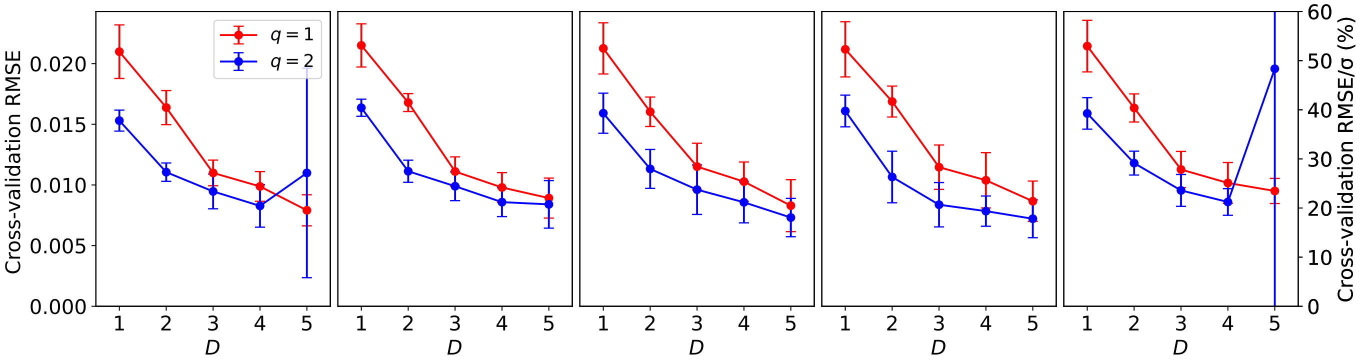

FIG. S5. Cross-validation analysis to assess the optimal complexity of the SISSO models for the C_4-products selectivity (S_{C_4}). Each panel correspond to one different outer-loop split. The validation root-mean-squared errors (RMSE) and error bars correspond to the average and standard deviation of five RMSE values obtained in the inner-loop splits of the nested cross-validation scheme. The y-axis on the right shows the RMSE as a percentage of the standard deviation of the target S_{C_4} over the entire dataset.

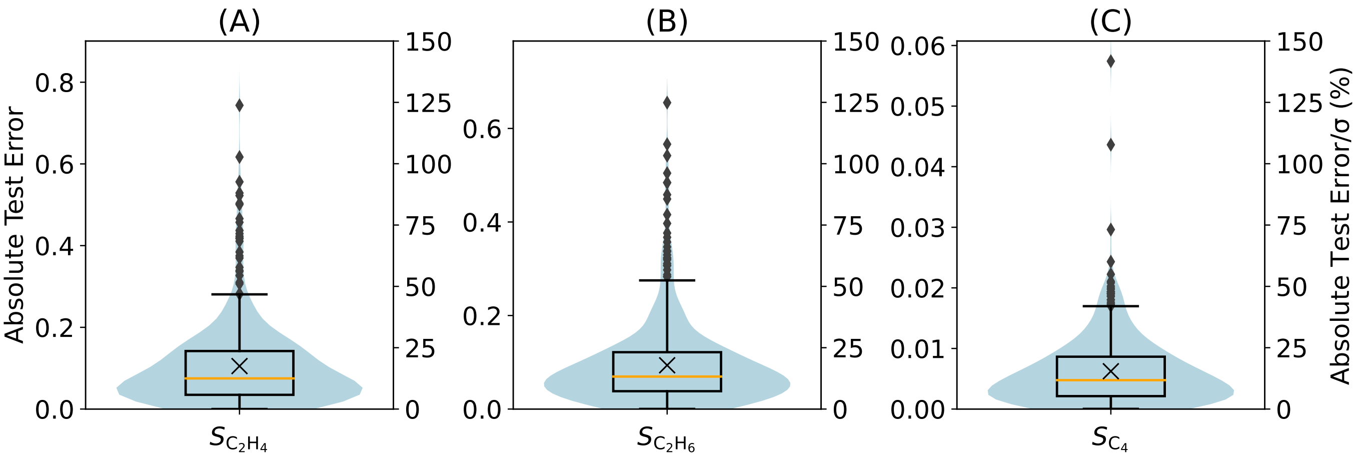

FIG. S6. Cross-validation analysis to assess the predictive performance of the SISSO models for the selectivity towards ethylene (A), ethane (B), and C_4 (C), respectively. Distribution of absolute test (prediction) errors evaluated in the outer-loop test sets of the nested cross-validation scheme. The SISSO models with complexity $q = 2$ and $D = 3$ were used to evaluate these errors. The width of the violins reflect the number of systems with a given error. The edges of the box indicate the 25 and 50%-iles of the distribution. The whiskers indicate the 0 and 90%-iles of the distribution. The cross indicates the mean error and the orange line indicates the median. The The y-axis on the right shows the error as a percentage of the standard deviation of each modelled target over the entire dataset. The distributions of errors correspond to 539 data points, i.e., the size of the entire dataset used to train the SISSO models.

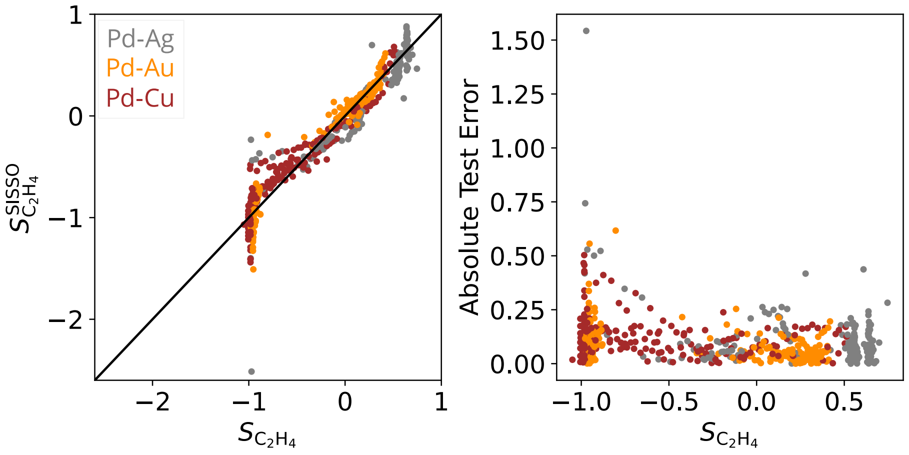

FIG. S7. Detailed analysis of test errors of SISSO models for the selectivity towards ethylene. The errors associated with Pd-Ag, Pd-Au, and Pd-Cu alloys are displayed in gray, orange, and brown, respectively.

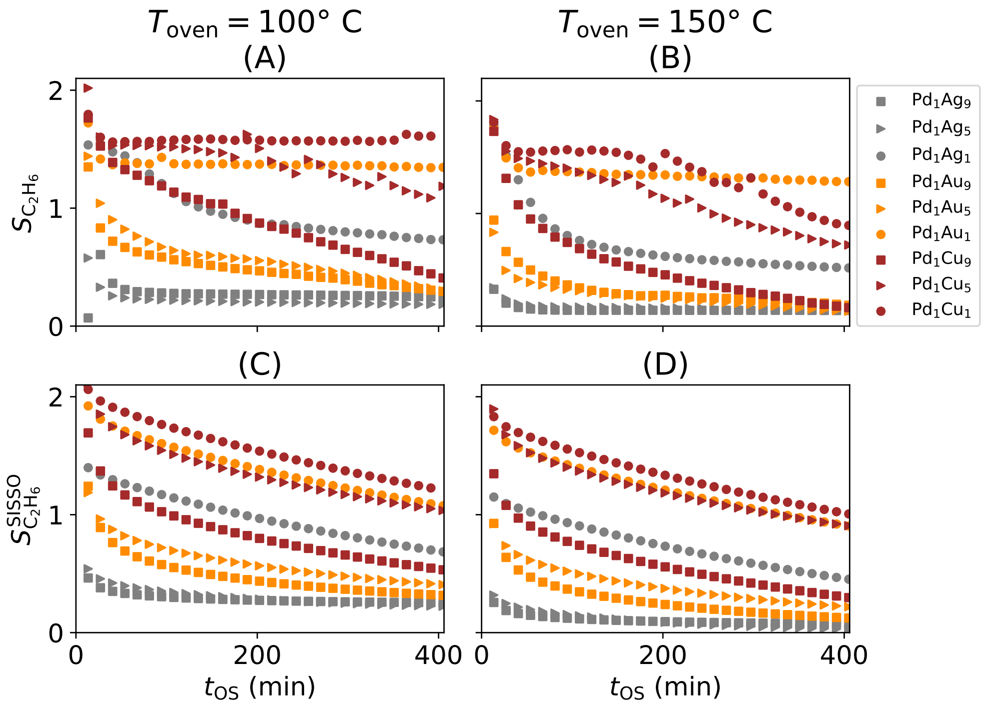

FIG. S8. The SISSO approach is used for modelling the selectivity towards ethane ($S_{C_2H_6}$, Eq. S1) as a function of materials' parameters and reaction conditions such as time on stream (t_{OS}) and temperature of oven (T_{oven}), see Eq. S9. (A) and (B): Ethane selectivity profiles measured at T_{oven} 100 and 150 °C, respectively. (C) and (D): Fits of the models identified by SISSO for the ethane selectivity ($S^{SISSO}_{C_2H_6}$) at T_{oven} 100 and 150 °C, respectively.

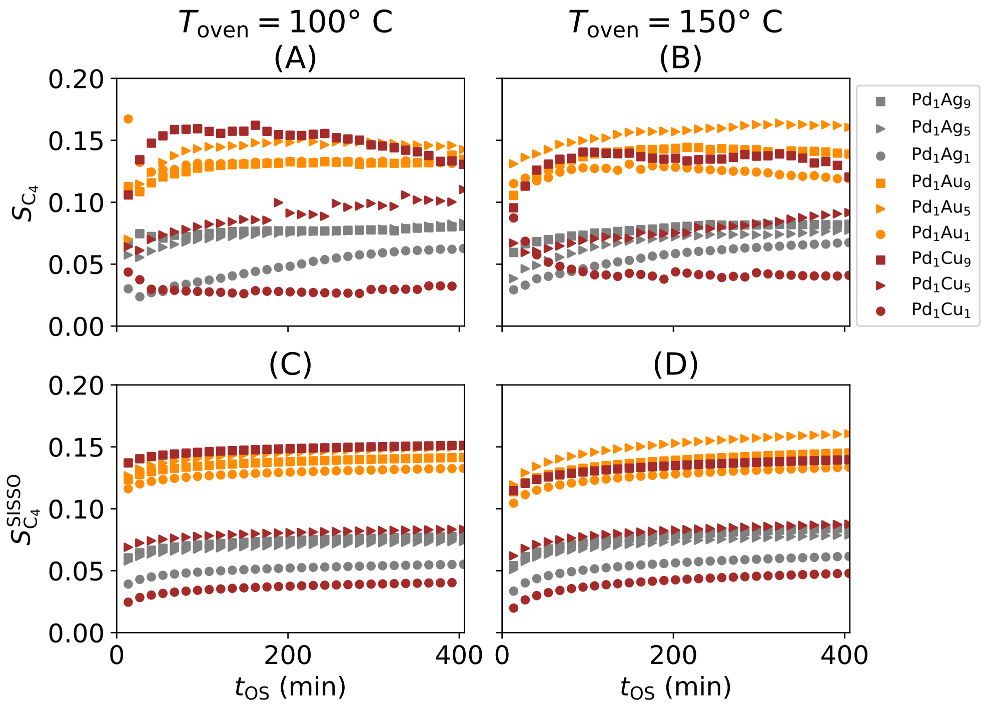

FIG. S9. The SISSO approach is used for modelling the selectivity towards C_4 (S_{C_4}, Eq. S2) as a function of materials' parameters and reaction conditions such as time on stream (t_{OS}) and temperature of oven (T_{oven}), see Eq. 5 in the main text. (A) and (B): C_4 selectivity profiles measured at T_{oven} 100 and 150 °C, respectively. (C) and (D): Fits of the models identified by SISSO for the C_4 selectivity ($S_{C_4}^{\text{SISSO}}$) at T_{oven} 100 and 150 °C, respectively.

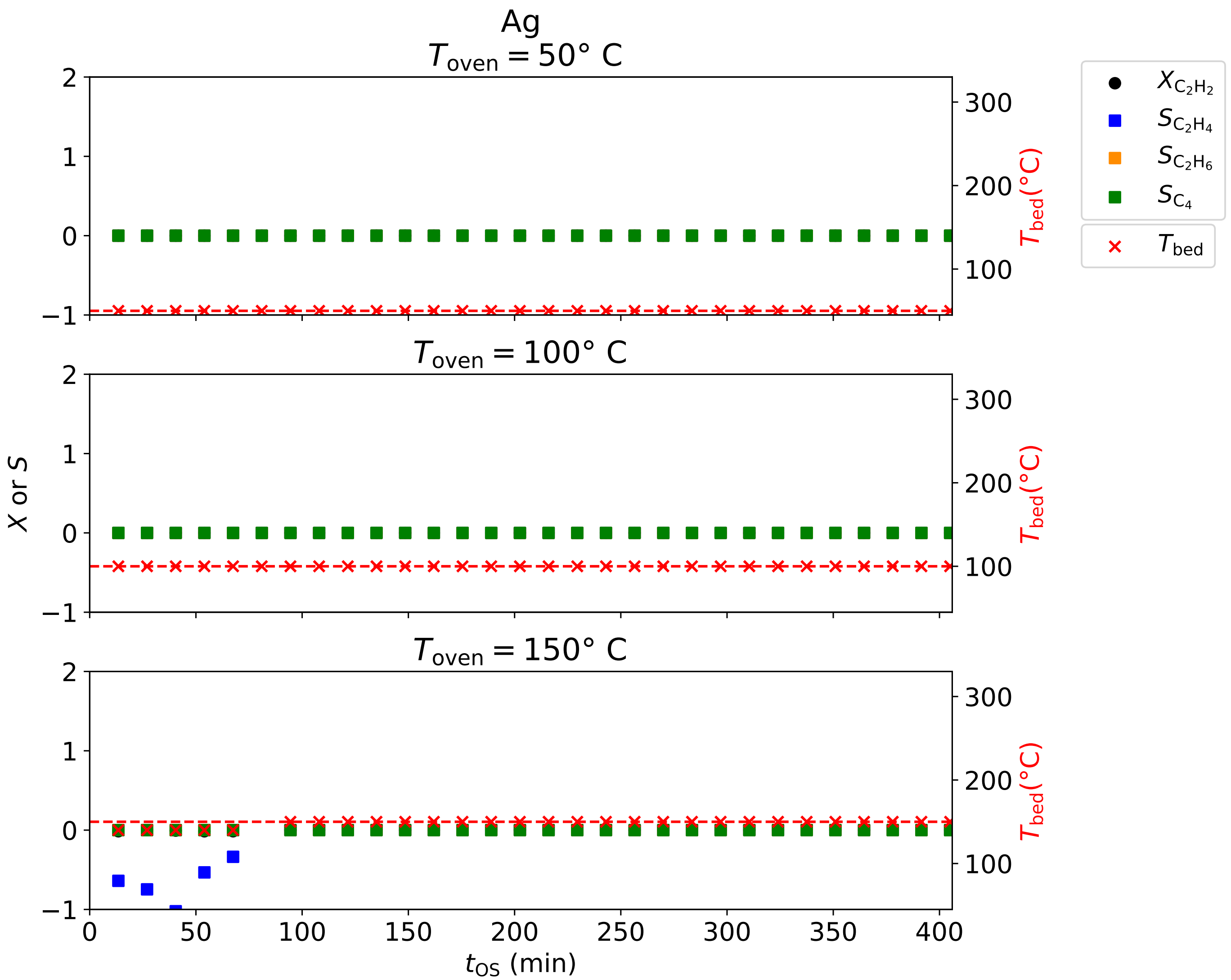

FIG. S10. Measured catalytic performance of ca. 1.0 wt % Ag/HS-α-Al_2O_3 in the selective hydrogenation of concentrated acetylene streams (see details in experimental section). The red horizontal dashed lines indicate the set reactor oven temperature (T_{oven}). The temperature measured inside the catalyst bed (T_{bed}) during the reaction is indicated by the red crosses.

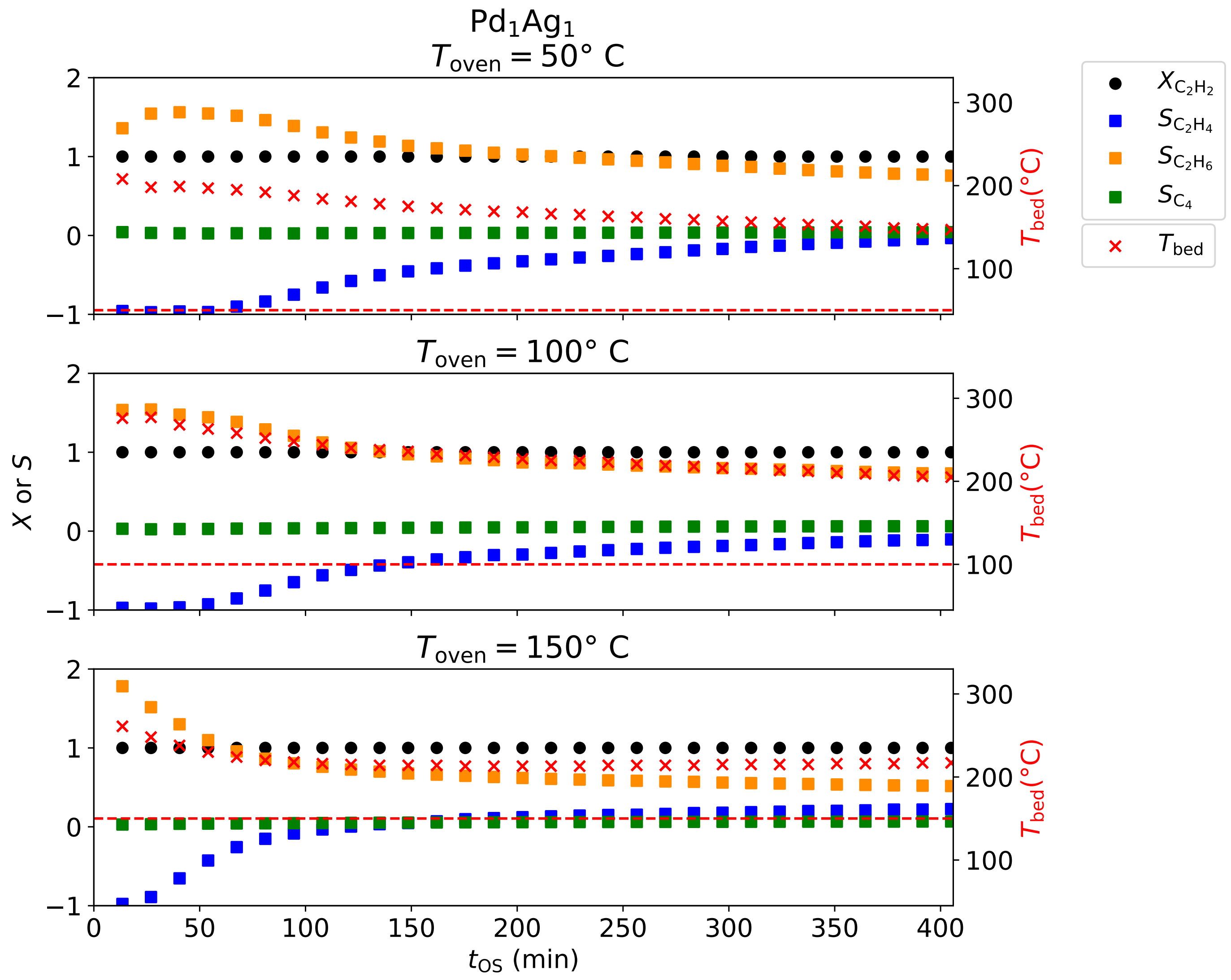

FIG. S11. Measured catalytic performance of ca. 1.0 wt % PdAg/HS-α-Al_2O_3 in the selective hydrogenation of concentrated acetylene streams (see details in experimental section). The red horizontal dashed lines indicate the set reactor oven temperature (T_{oven}). The temperature measured inside the catalyst bed (T_{bed}) during the reaction is indicated by the red crosses.

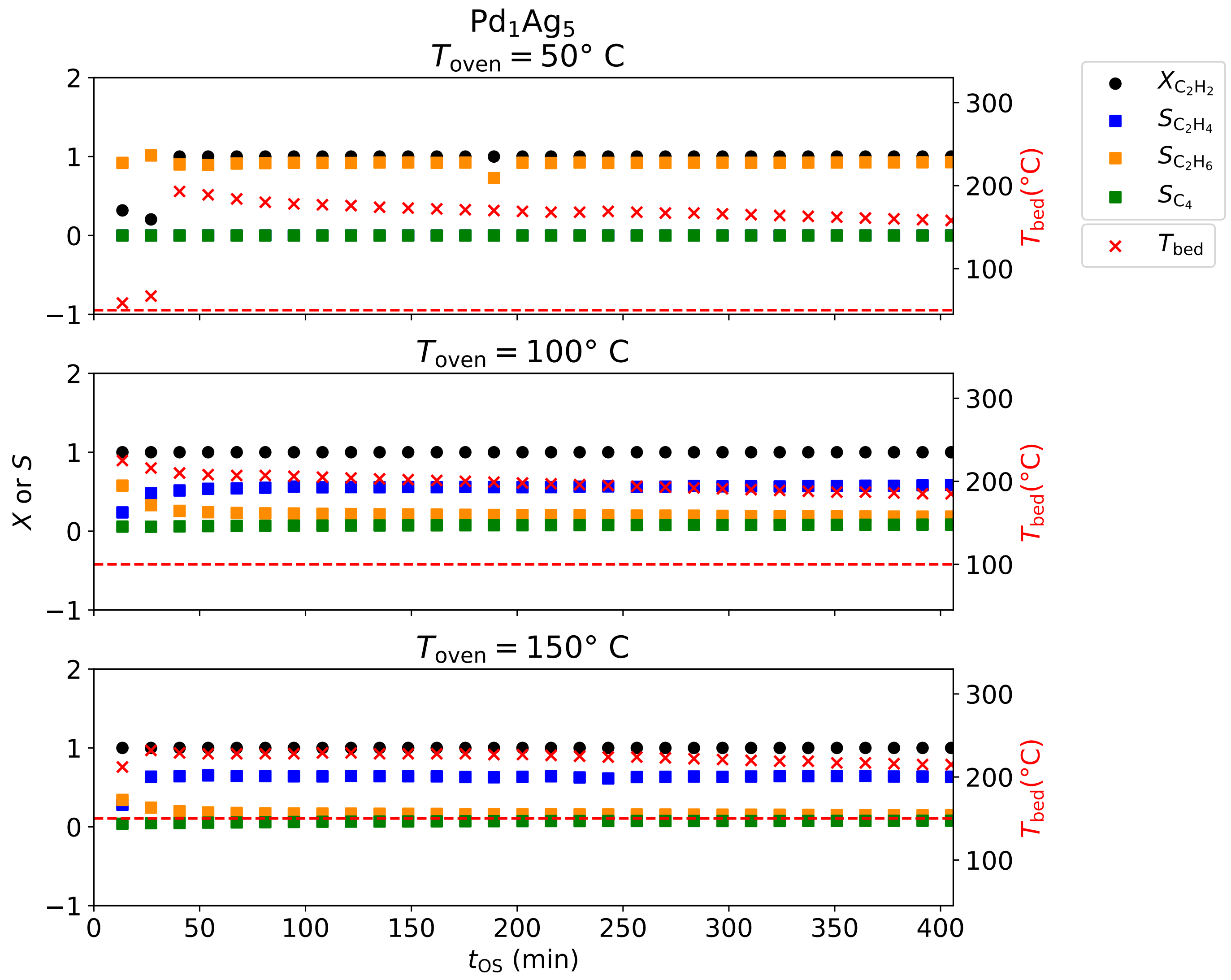

FIG. S12. Measured catalytic performance of ca. 1.0 wt % $PdAg_5$/HS-α-Al_2O_3 in the selective hydrogenation of concentrated acetylene streams (see details in experimental section). The red horizontal dashed lines indicate the set reactor oven temperature (T_{oven}). The temperature measured inside the catalyst bed (T_{bed}) during the reaction is indicated by the red crosses.

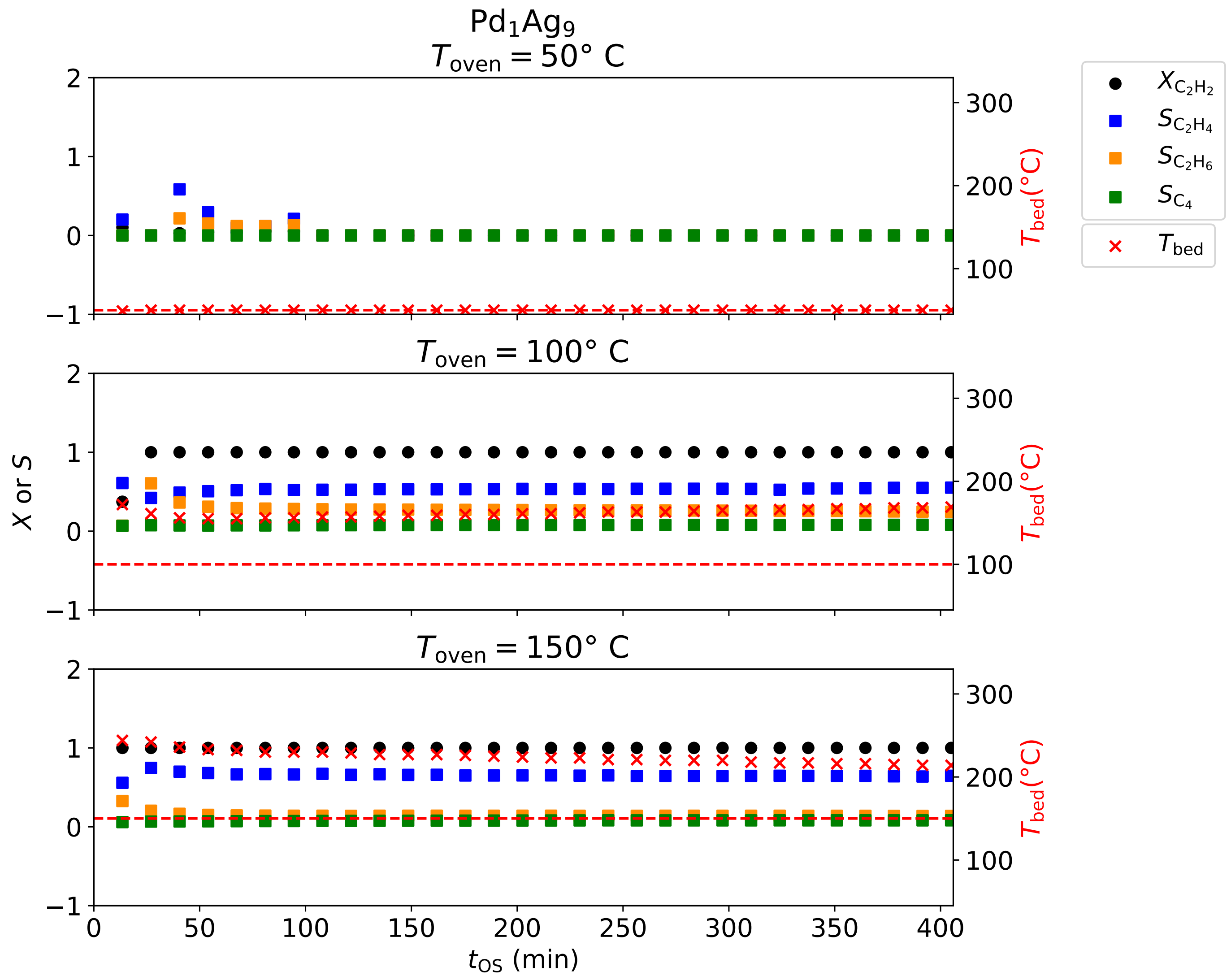

FIG. S13. Measured catalytic performance of ca. 1.0 wt % $PdAg_9$/HS-α-Al_2O_3 in the selective hydrogenation of concentrated acetylene streams (see details in experimental section). The red horizontal dashed lines indicate the set reactor oven temperature (T_{oven}). The temperature measured inside the catalyst bed (T_{bed}) during the reaction is indicated by the red crosses.

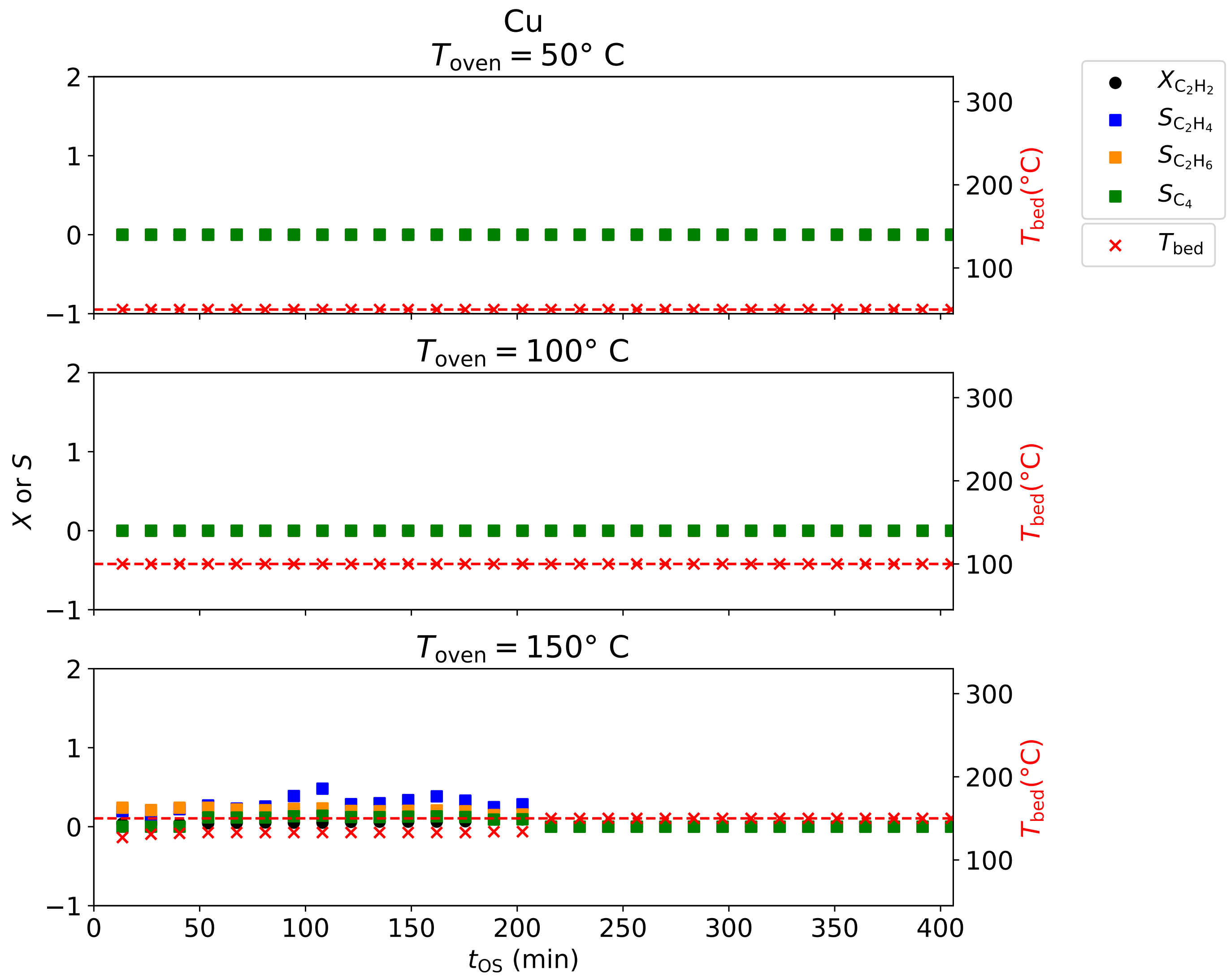

FIG. S14. Measured catalytic performance of ca. 1.0 wt % Cu/HS-α-Al_2O_3 in the selective hydrogenation of concentrated acetylene streams (see details in experimental section). The red horizontal dashed lines indicate the set reactor oven temperature (T_{oven}). The temperature measured inside the catalyst bed (T_{bed}) during the reaction is indicated by the red crosses.

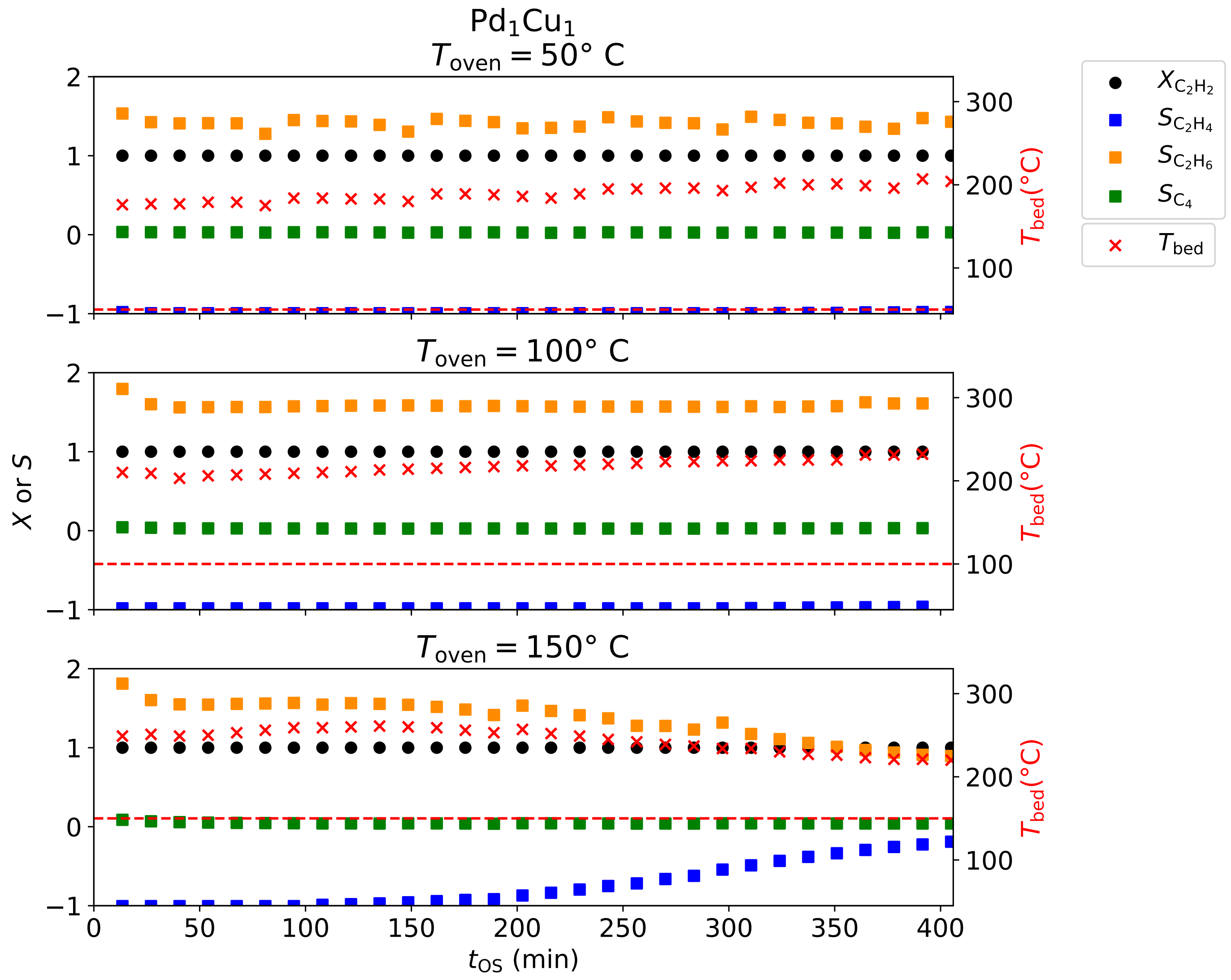

FIG. S15. Measured catalytic performance of ca. 1.0 wt % PdCu/HS-α-Al_2O_3 in the selective hydrogenation of concentrated acetylene streams (see details in experimental section). The red horizontal dashed lines indicate the set reactor oven temperature (T_{oven}). The temperature measured inside the catalyst bed (T_{bed}) during the reaction is indicated by the red crosses.

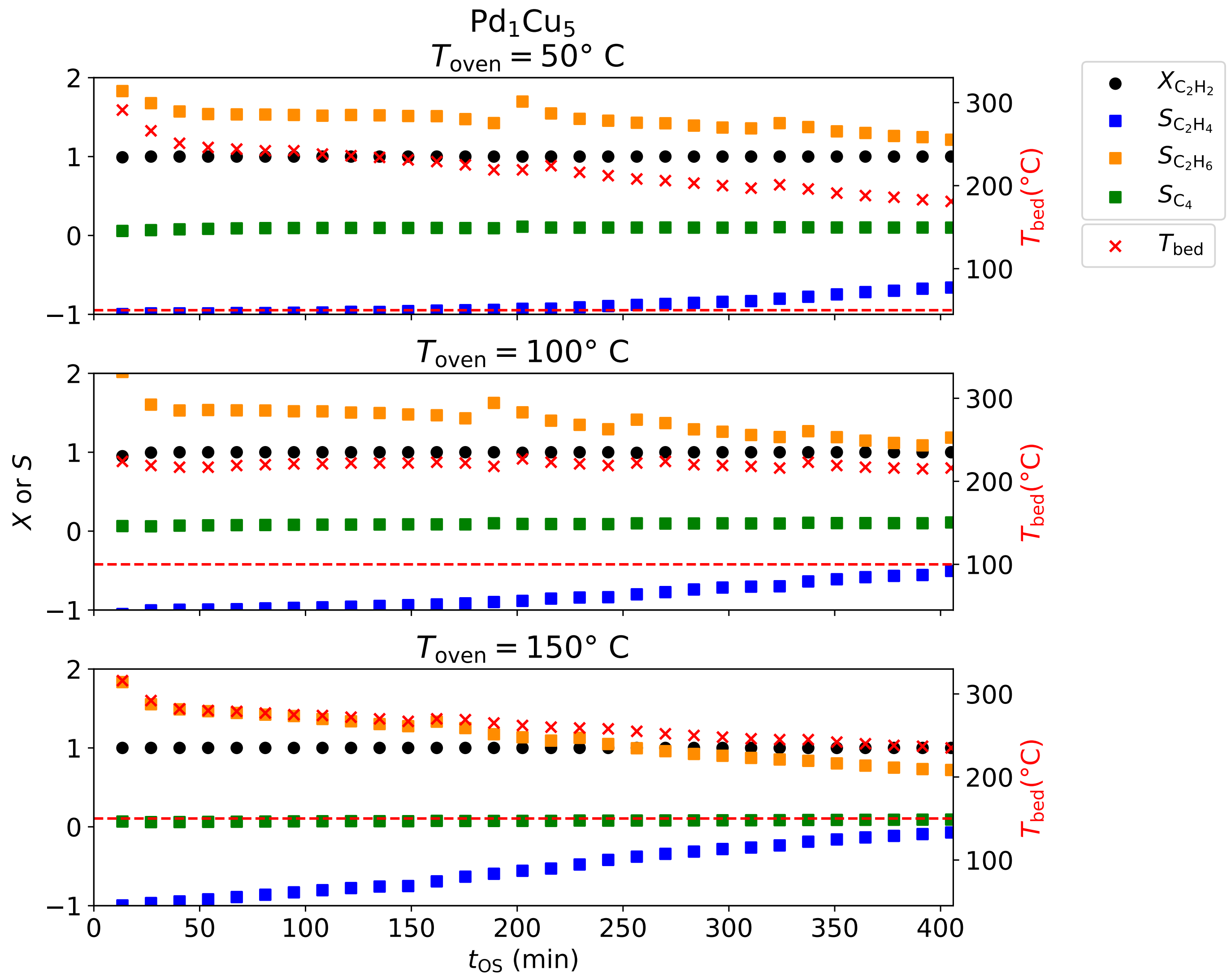

FIG. S16. Measured catalytic performance of ca. 1.0 wt % $PdCu_5$/HS-α-Al_2O_3 in the selective hydrogenation of concentrated acetylene streams (see details in experimental section). The red horizontal dashed lines indicate the set reactor oven temperature (T_{oven}). The temperature measured inside the catalyst bed (T_{bed}) during the reaction is indicated by the red crosses.

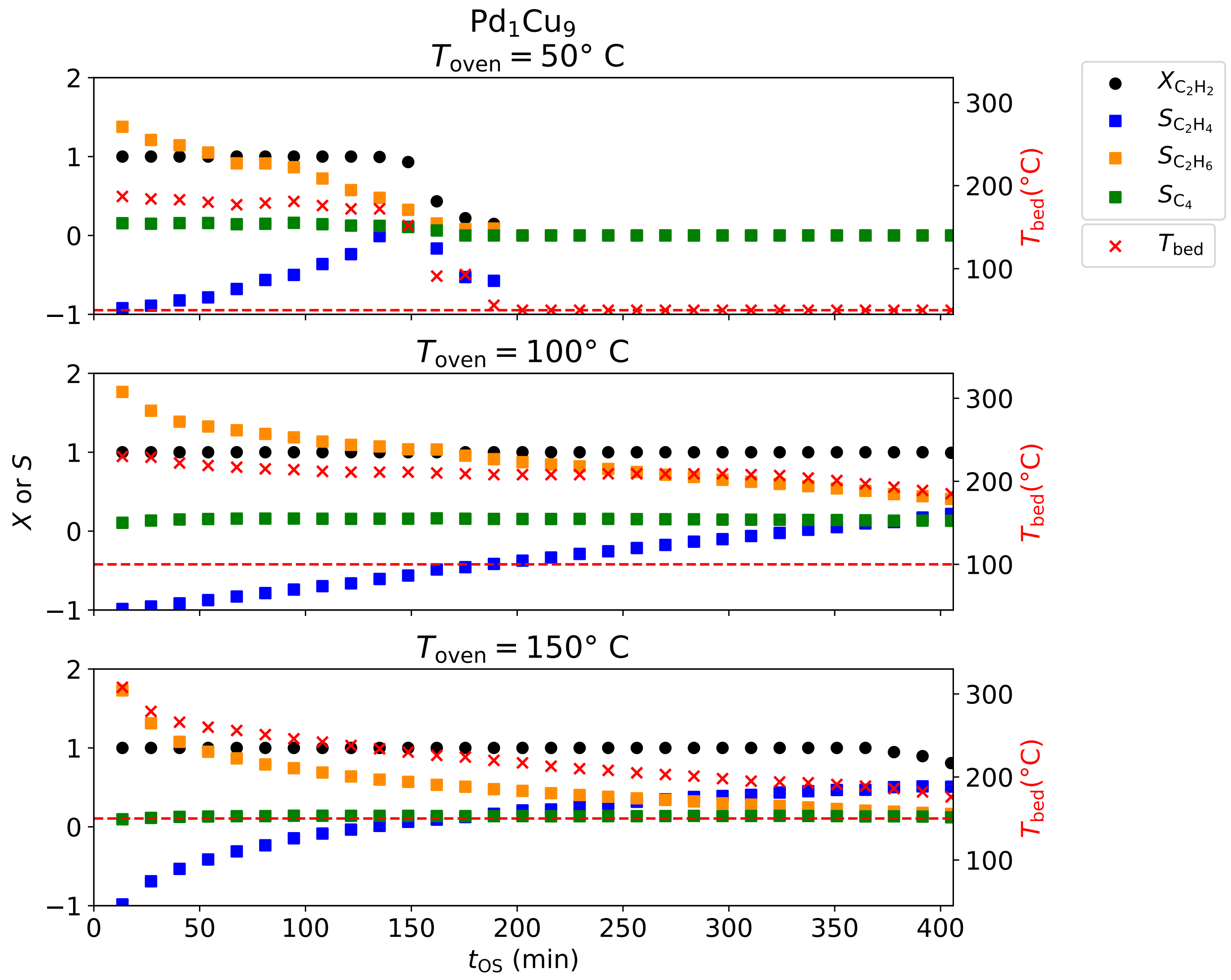

FIG. S17. Measured catalytic performance of ca. 1.0 wt % $PdCu_9$/HS-α-Al_2O_3 in the selective hydrogenation of concentrated acetylene streams (see details in experimental section). The red horizontal dashed lines indicate the set reactor oven temperature (T_{oven}). The temperature measured inside the catalyst bed (T_{bed}) during the reaction is indicated by the red crosses.

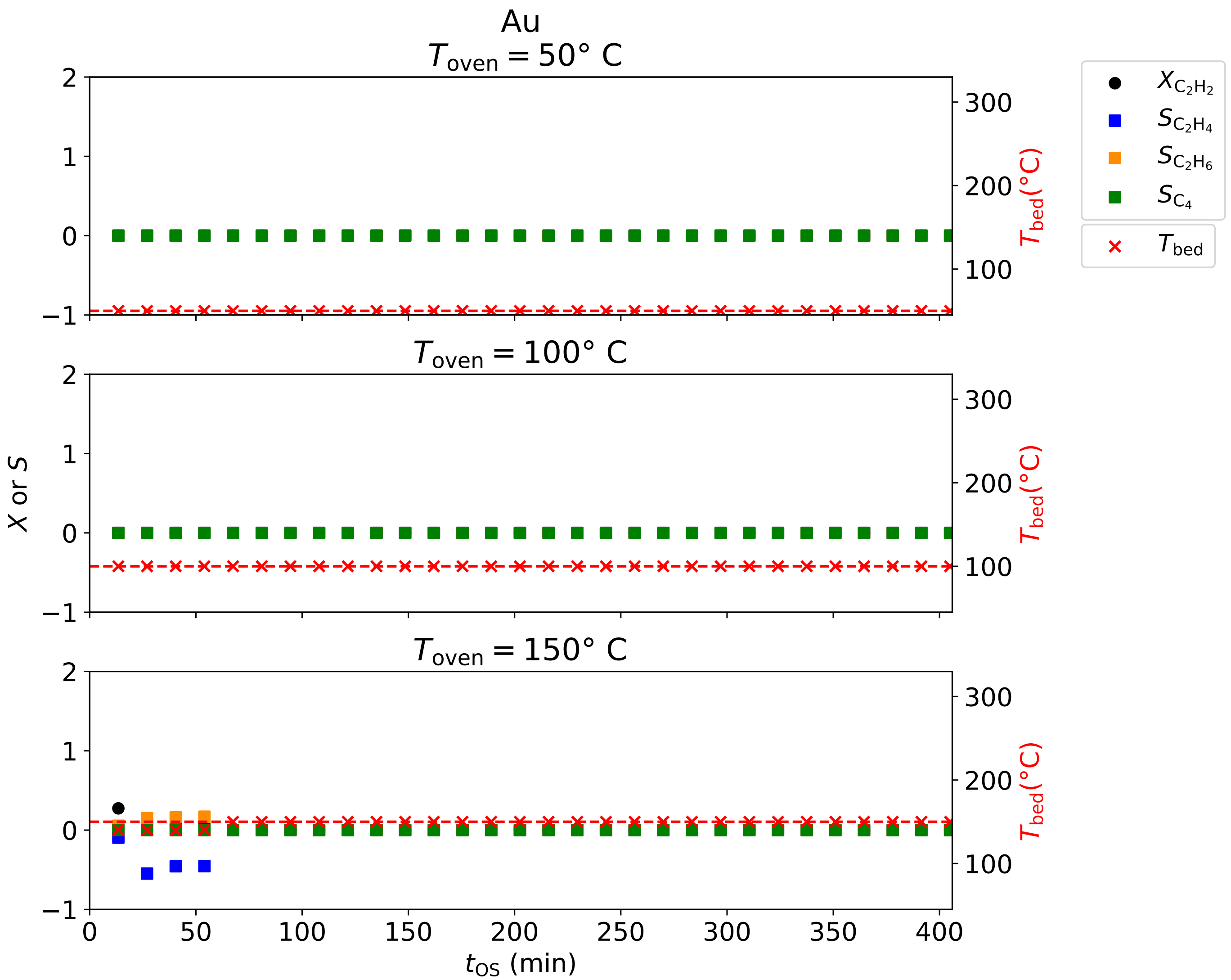

FIG. S18. Measured catalytic performance of ca. 1.0 wt % Au/HS-α-Al_2O_3 in the selective hydrogenation of concentrated acetylene streams (see details in experimental section). The red horizontal dashed lines indicate the set reactor oven temperature (T_{oven}). The temperature measured inside the catalyst bed (T_{bed}) during the reaction is indicated by the red crosses.

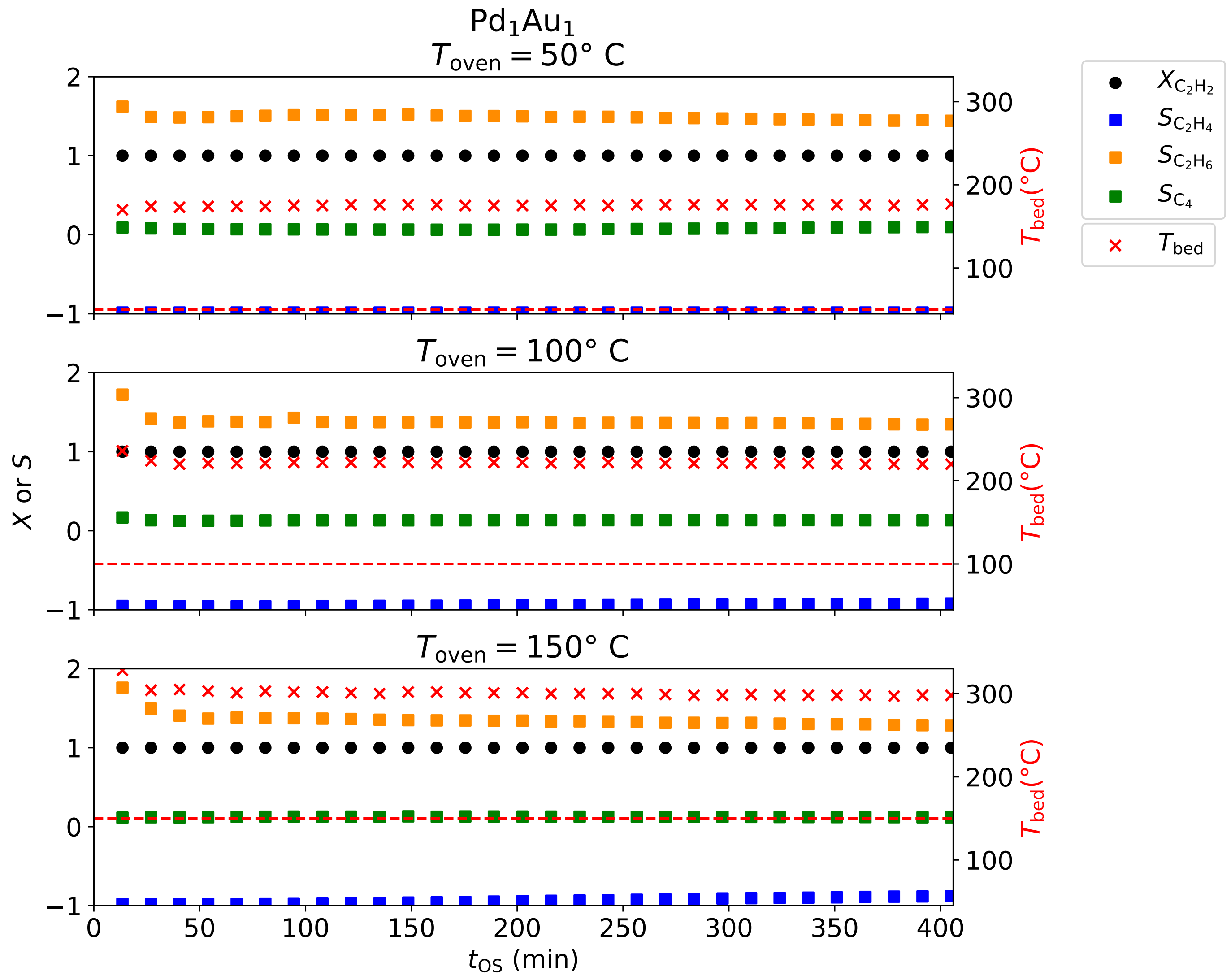

FIG. S19. Measured catalytic performance of ca. 1.0 wt % PdAu/HS-α-Al_2O_3 in the selective hydrogenation of concentrated acetylene streams (see details in experimental section). The red horizontal dashed lines indicate the set reactor oven temperature (T_{oven}). The temperature measured inside the catalyst bed (T_{bed}) during the reaction is indicated by the red crosses.

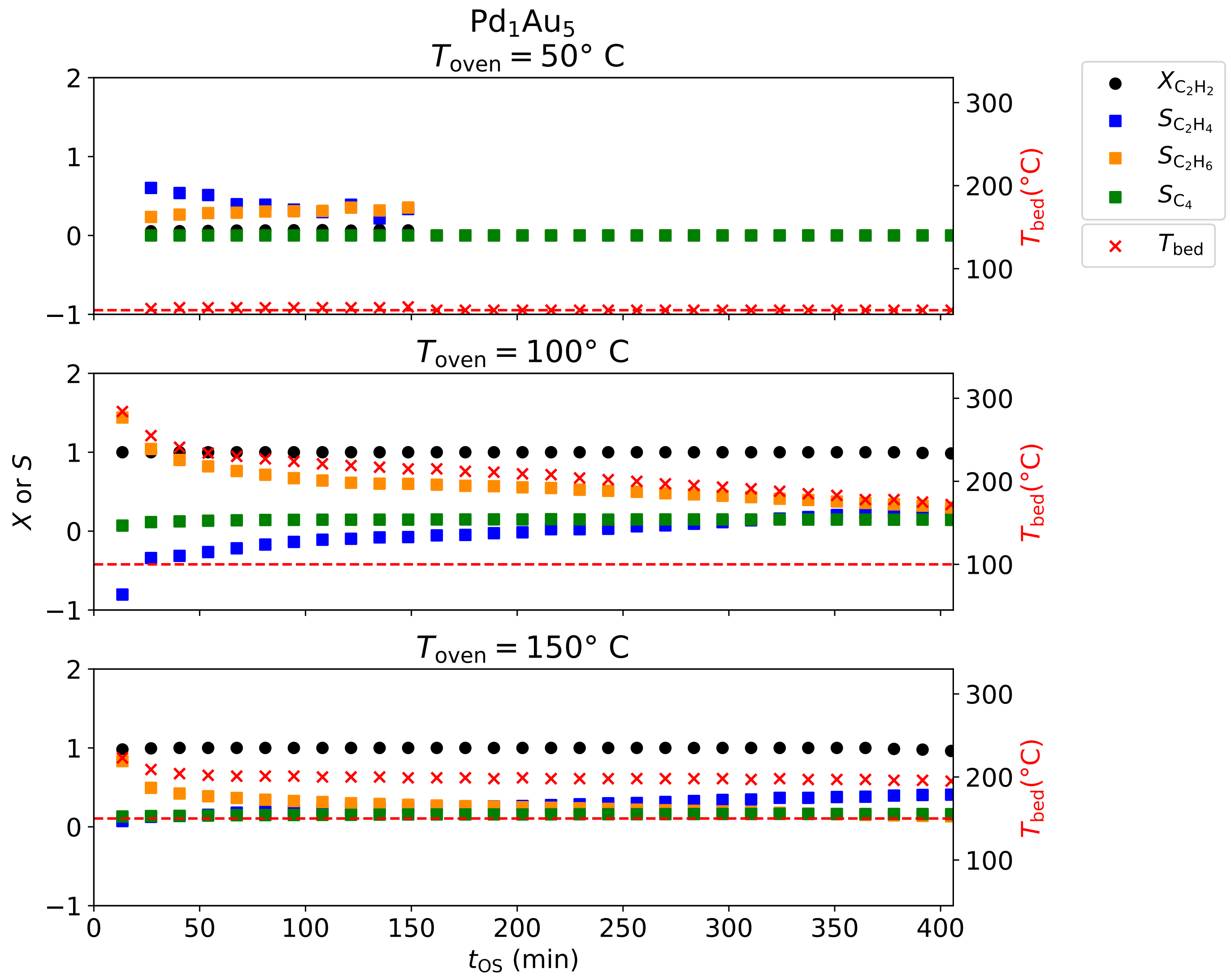

FIG. S20. Measured catalytic performance of ca. 1.0 wt % PdAu$_5$/HS-α-Al_2O_3 in the selective hydrogenation of concentrated acetylene streams (see details in experimental section). The red horizontal dashed lines indicate the set reactor oven temperature (T_{oven}). The temperature measured inside the catalyst bed (T_{bed}) during the reaction is indicated by the red crosses.

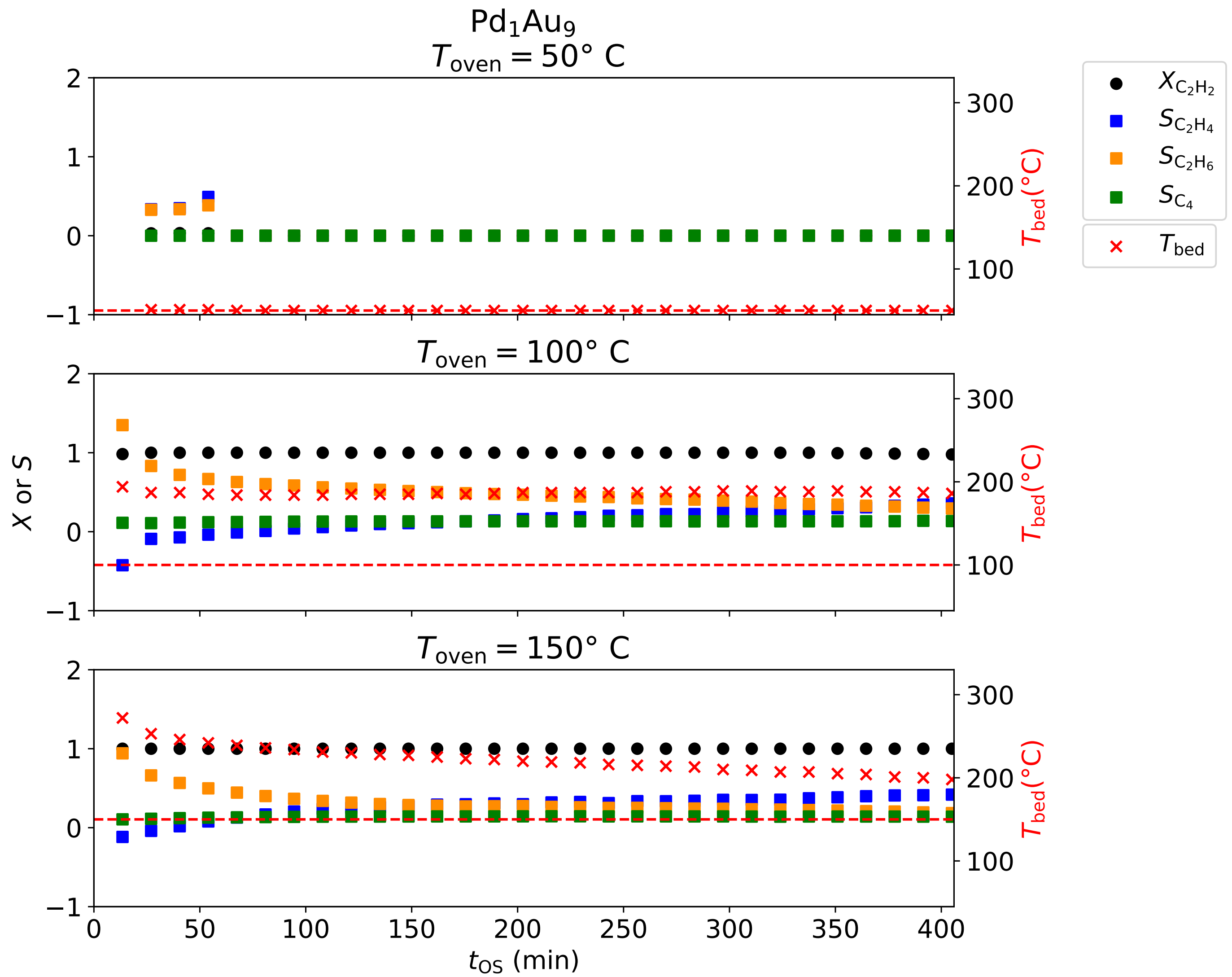

FIG. S21. Measured catalytic performance of ca. 1.0 wt % $PdAu_9/HS\text{-}\alpha\text{-}Al_2O_3$ in the selective hydrogenation of concentrated acetylene streams (see details in experimental section). The red horizontal dashed lines indicate the set reactor oven temperature (T_{oven}). The temperature measured inside the catalyst bed (T_{bed}) during the reaction is indicated by the red crosses.
